# A medium density genetic map and QTL for behavioral and production traits in Japanese quail

**DOI:** 10.1186/s12864-014-1210-9

**Published:** 2015-01-22

**Authors:** Julien Recoquillay, Frédérique Pitel, Cécile Arnould, Sophie Leroux, Patrice Dehais, Carole Moréno, Ludovic Calandreau, Aline Bertin, David Gourichon, Olivier Bouchez, Alain Vignal, Maria Ines Fariello, Francis Minvielle, Catherine Beaumont, Christine Leterrier, Elisabeth Le Bihan-Duval

**Affiliations:** INRA, UR83 Recherches Avicoles, F-37380 Nouzilly, France; UMR INRA/Génétique Physiologie et Systèmes d’Elevage, INRA, F-31326 Castanet-Tolosan, France; INPT ENSAT / Génétique Physiologie et Systèmes d’Elevage, INRA, F-31326 Castanet-Tolosan, France; INPT ENVT Génétique Physiologie et Systèmes d’Elevage, INRA, F-31326 Castanet-Tolosan, France; INRA, UMR85 Physiologie de la Reproduction et des Comportements, F-37380, Nouzilly, France; CNRS, UMR7247, F-37380 Nouzilly, France; Université François Rabelais de Tours, F-37000 Tours, France; IFCE, F-37380 Nouzilly, France; INRA, Sigenae UR875 Biométrie et Intelligence Artificielle, F-31326 Castanet-Tolosan, France; UE1295 Pôle d’Expérimentation Avicole de Tours, F-37380 Nouzilly, France; INRA, GeT-PlaGe Genotoul, F-31326 Castanet-Tolosan, France; Institut Pasteur, Montevideo, Uruguay; INRA, UMR1313 GABI Génétique Animale et Biologie Intégrative, F-78530 Jouy-en-Josas, France

## Abstract

**Background:**

Behavioral traits such as sociability, emotional reactivity and aggressiveness are major factors in animal adaptation to breeding conditions. In order to investigate the genetic control of these traits as well as their relationships with production traits, a study was undertaken on a large second generation cross (F2) between two lines of Japanese Quail divergently selected on their social reinstatement behavior. All the birds were measured for several social behaviors (social reinstatement, response to social isolation, sexual motivation, aggression), behaviors measuring the emotional reactivity of the birds (reaction to an unknown object, tonic immobility reaction), and production traits (body weight and egg production).

**Results:**

We report the results of the first genome-wide QTL detection based on a medium density SNP panel obtained from whole genome sequencing of a pool of individuals from each divergent line. A genetic map was constructed using 2145 markers among which 1479 could be positioned on 28 different linkage groups. The sex-averaged linkage map spanned a total of 3057 cM with an average marker spacing of 2.1 cM. With the exception of a few regions, the marker order was the same in Japanese Quail and the chicken, which confirmed a well conserved synteny between the two species. The linkage analyses performed using QTLMAP software revealed a total of 45 QTLs related either to behavioral (23) or production (22) traits. The most numerous QTLs (15) concerned social motivation traits. Interestingly, our results pinpointed putative pleiotropic regions which controlled emotional reactivity and body-weight of birds (on CJA5 and CJA8) or their social motivation and the onset of egg laying (on CJA19).

**Conclusion:**

This study identified several QTL regions for social and emotional behaviors in the Quail. Further research will be needed to refine the QTL and confirm or refute the role of candidate genes, which were suggested by bioinformatics analysis. It can be hoped that the identification of genes and polymorphisms related to behavioral traits in the quail will have further applications for other poultry species (especially the chicken) and will contribute to solving animal welfare issues in poultry production.

**Electronic supplementary material:**

The online version of this article (doi:10.1186/s12864-014-1210-9) contains supplementary material, which is available to authorized users.

## Background

Groups of animals are often large in modern poultry breeding conditions, resulting in repeated group disruption and encouraging the expression of aggressive behaviors [1]. Studies on the social dynamics induced by breeding in large groups have shown that the size of the group is not the only factor behind the appearance of aggressive behaviors. The space available per individual and therefore the distance between individuals also have a role [2-5]. Bird sociability is another aspect to consider in adaptation to the group. Studies on two lines of Japanese Quail divergently selected for their social reinstatement behavior [6], showed that quail with a higher level of social reinstatement (HSR) were quicker to rejoin conspecifics and formed shorter distances between individuals than those with a lower level of social reinstatement behavior (LSR) at a young age, before becoming similar at adult age [7]. Moreover, the HSR line seemed to be more tolerant of group disruption (social isolation followed by reintroduction of familiar or unfamiliar conspecifics in groups) than the LSR line [8]. These results highlight the importance of social motivation as a factor contributing to the ability of a bird to adapt to modern breeding conditions.

In a previous study [9], we used a second generation (F2) cross between two lines of quail divergently selected for their social reinstatement [6] to estimate the genetic parameters of social motivation and its relationships with other important behaviors (such as aggression, sexual motivation, emotional reactivity) and production traits. Our results showed a significant genetic contribution to the phenotypic variability of behavioral traits. This has also been demonstrated at the molecular level, as QTLs were detected for the duration of tonic immobility in the chicken [10] and in Japanese Quail [11,12], for emotional reactivity in open-field [13] and for the propensity to receive or perform feather pecking [14,15] in the chicken. Possible genetic links between emotional reactivity and production traits were also evidenced in an F2 cross between the Red Junglefowl and the White Leghorn, where the two most significant growth QTL (*Growth1* and *Growth2* loci) were co-located with tonic immobility or response to a novel object QTL [10,16]. It was later shown that chickens with alternative homozygous genotypes at the *Growth1* locus differed in several emotional and social reactions [17].

The aim of the present study was to investigate further the genetic control of social and emotional behaviors, and their relationships with production traits, by genome-wide QTL detection in the F2 cross between two lines of quail divergently selected for social reinstatement, i.e. the distance run on a treadmill to rejoin conspecifics [6]. As no whole-genome marker panel is publicly available in Japanese Quail, and as only a few low density genetic maps have been constructed with AFLP markers [18], microsatellites [19] or both types of marker [20], informative markers were also developed through high-throughput sequencing of the divergent lines to construct the first genetic map of the whole genome based on SNP markers.

## Methods

### Animals

Two divergent lines bred and reared at INRA experimental unit 1295 (UE PEAT, F-37380 Nouzilly, France) were used in the experiment. These lines with either high or low social reinstatement behavior (HSR or LSR, respectively) have been divergently selected by the treadmill test (detailed below) on their propensity to rejoin a group of conspecifics when 10 days old, while maintaining a constant duration of tonic immobility across generations [6]. They differ consistently in their social motivation under various experimental conditions and in several other aspects of social behavior such as sexual motivation and aggressive behavior [21-25]. A reciprocal cross of these two lines was undertaken from the 49^th^ generation, using four HSR males and four LSR females to produce the HSR x LSR (H/L) cross and four LSR males and four HSR females to produce the LSR x HSR (L/H) cross. From this F1 generation, three H/L males were each mated with two H/L females and two L/H females, and three L/H males were mated with two H/L females and two L/H females. Two females from the F1 generation died during the experiment and were replaced by individuals with a similar genetic background, resulting in 26 F1 females. A total of 731 F2 quail chicks (360 males and 371 females) were used for QTL detection analyses, with an average of 32 chicks produced by F1 females and 120 by F1 males in 6 successive batches. Animal care and experimental procedures were in accordance with French and European regulations. The Experimental Unit where birds were kept is registered by the ministry of Agriculture under the license number B-37-175-1 for animal experimentation. All behavioral tests were approved by the ethics committee in Animal Experimentation of Val de Loire (permit number 2011-07-10).

### Testing procedures

#### *Behavioral trait*s

The detailed procedure for the behavioral tests used in this study can be found in the previous study by Recoquillay et al. [9].

### Social motivation (DistIso and DistSR)

Social motivation was measured by two behavioral tests. The first, undertaken in a familiar arena with water and food when the chicks were 1 to 3 days old, estimated social motivation by the distance the chick walked at the periphery of the arena. This variable was referred as DistIso: the higher its value, the more sociable the chick was considered to be, as the distance walked was evaluated as a reinstatement behavior because the chick was looking for its conspecifics [22]. The second test, the treadmill test, was performed when the quail were 6 to 8 days old. The procedure was similar to that used for the selection of the two divergent lines by Mills and Faure [6]. Each quail was placed on the center of the treadmill with the possibility of reaching two extremities, either a cage with conspecifics or a dead end. Social motivation was estimated by the distance run on the treadmill in order to rejoin the conspecifics. This variable, recorded as DistSR, is higher in more socially motivated birds.

### Emotional reactivity (TI and HeadNO)

Emotional reactivity was measured by two tests. The first, the tonic immobility test, was performed when the quail were 9 to 10 days old. During this test, tonic immobility was induced by restraining the animal on its back: the longer the time needed for the bird to redress itself (referred to as TI), the more fearful the bird. The second test measured birds’ emotional reactivity to a novel object. This test was performed when the chicks were 37 to 38 days old. During this test, an unknown object was presented to an individual in front of its cage in such a manner that the animals in other cages could not see it. The number of scans during which the quail had its head through the wire in the front of the cage (touching the object or not) was recorded and referred to as HeadNO. The more often the bird passed its head through the wire, trying to reach the novel object, the less fearful it was considered to be [26,27].

### Sexual motivation and aggressiveness (GentleP, AgrP and Mount)

Aggressiveness and sexual motivation were evaluated in the same arena at 55 to 56 days and 62 days respectively, in all male quail. In each test, the bird was confronted with either a mirror (aggressiveness test) or a stuffed female (sexual motivation). For the aggressiveness test, two behavioral variables were noted: the number of aggressive pecks at the mirror, referred as AgrP, and the number of gentle pecks, referred as GentleP. The first variable measured the aggressiveness and the second the exploratory social tendency of the bird [28]. Sexual motivation was measured by the number of mounts the male performed on the stuffed female and was referred to as Mount: the higher this variable, the more sexually active the bird.

#### Production traits

Birds were weighed at 17 days of age (W17) and after the last behavioral test, i.e. at 65 days of age (W65). The age when the first egg was laid (AFEgg) and the number of eggs laid (NEgg) until week 24 were recorded as well as the mean egg weight (WEgg) calculated from eggs laid during weeks 12 and 13.

### Choice of markers and genotyping

To maximize the informativity of our SNP panel, markers were developed directly from the genomic sequences of the HRS and LSR lines. Samples from ten F0 individuals from each line were pooled and sequenced (paired-ends, 100 bp) on two lanes of a HiSeq 2000 sequencer (Illumina) according to the manufacturer’s instructions (TruSeq kits). In the absence of an available genome sequence for the Japanese Quail, the readings of the two divergent lines were mapped to the chicken genome assembly (galGal3, GallusWU2.58), and the bwasw option of the bwa software was used to improve the heterologous alignment [29]. Readings were filtered for single mapping, absence of N, and identity level over 70% before SNP detection. The SNP markers selected had to be biallelic, and positioned outside repeated regions and could not originate from an insertion/deletion event. A quality filter was applied (mapping quality > 30, read depth > 10), and SNPs resulting from transversion or found close to another SNP (in the 120 flanking base pairs) were excluded. Only SNPs with an allele frequency of 0 in one pool and 1 in the other pool were then selected, corresponding to line-specific SNPs in the 20 individuals sequenced. After Illumina scoring, final selection was made in order to optimize the SNP distribution on the genome with the chicken genetic map as the basis. Genotyping was performed with the iSelect method from Illumina. Genotype calling was carried out with the GenomeStudio software (GenomeStudio V2010.1, Illumina). Genotypes were filtered for call rate (>0.85), call frequency (>0.50) and cluster dispersion. Markers with an unexpected deviation from Hardy-Weinberg equilibrium (p < 10^−6^) were removed with PLINK [30].

### Map construction

The genetic map was constructed using CriMap version 2.504 software [31]. First the PREPARE function was used to verify the genotyping data for incompatibilities with Mendelian inheritance, and errors were rechecked and data were excluded when necessary. The TWOPOINT function was used with a LOD score threshold of 50.0 in order to create the linkage groups. Markers belonging to the same linkage groups were then analyzed with two iterations using the BUILD and FLIPS functions. In the first step, the BUILD option was used with a LOD score threshold model of 6.0, and the order of loci generated was examined with the FLIPS and CHROMPIC option. In the second step, the BUILD option was used taking a LOD score of 3.0 on the previously generated order as a basis in order to add the loci excluded during the first analysis. The new order was again examined with the FLIPS and CHROMPIC functions. Map distances, expressed in cM, were calculated from the Kosambi distance.

### QTL detection

QTLs were detected by QTLMAP software (version 0.9.6) developed for outbred populations and applied to a mixture of half and full sib families [32]. The methodology used was the linkage analysis (LA) with a heteroskedastic model assuming non-equal residual (within sire) variances. No assumptions were made about the fixation of the QTL alleles in the founder populations and different QTL effects were estimated separately for each family analyzed. In the present study, we performed two complementary analyses. The first, modeling only sire QTLs, allowed precise estimation of the QTL effect (because of the high number of offspring per sire) but on a limited number of parents. The second, including also dam QTLs estimated from at least 20 offspring, was expected to detect more QTLs by increasing the potential number of heterozygous parents. In addition to the fixed effects of the batch and sex, a polygenic and a QTL effects were estimated for each parent. The likelihood ratio test between the hypothesis of the existence of a QTL (H_1_) and the hypothesis of the absence of a QTL (H_0_) was calculated on a given chromosome at each position every 0.5 cM. The rejection threshold of H_0_ and the chromosome-wide level of significance of H_1_ were estimated by 10,000 simulations under the H_0_ hypothesis with a polygenic model using heritability coefficients estimated by Recoquillay et al. [9]. Genome-wide level of significance (P_*genome-wide*_) was derived from the chromosome-wide probability (P_*chromosome-wide*_) by the following Bonferroni correction [33], where *r* is the ratio between the length of the chromosome tested and the total length of the genome analyzed:

$$ {P}_{genome- wise}=1-{\left(1,-,{P}_{chromosome- wide}\right)}^{1/r} $$

QTL were declared to be genome-wide significant when *P*_*genome-wide*_ < 0.05, and chromosome-wide significant when *P*_*chromosome-wide*_ ≤ 0.05. The QTL confidence interval (95%) was estimated by the LOD drop-off method (1 LOD was used). Substitution effects of the QTLs were calculated for each sire or dam and their significance tested with a *t*-test. The effect of the QTL was estimated as the average of the significant parent’s effects (P < 0.05) and expressed in phenotypic standard deviation.

### QTL comparisons between chicken and quail

Each of our SNPs was attributed to a specific coordinate in the chicken genome through the first bwa alignment against the previous version of the genome (galGal3) and updated by blast analysis [34] of the 1000 bp flanking sequence of the SNP against galGal4 [35]. The Chicken QTL Database [36,37] was used in order to assess the genetic environment in the chicken genome at the coordinates of the markers flanking the likeliest position of the QTL and at the coordinates of the markers flanking the confidence interval of the QTL. The results of these comparisons are shown in Additional file 1: Table S1.

## Results

### Sequencing and SNP detection

A total of 19.2 and 23.3 billion bases were obtained for the HSR and LSR lines, respectively. Forty-two percent of the readings mapped to the chicken genome, leading to an average coverage of 50.7% (Additional file 2: Figure S1). From positions covered by more than 10 reads, we observed a total of 7,178,545 SNPs between both lines, with a mean depth of 31.6. The SNP selection pipeline gave, from these positions, a set of 4774 putative SNPs, to which we added 1226 quail SNPs from other programs ([38]; Bed’hom, personal communication). The final set comprised 6000 markers, equally distributed along the chicken genetic map (Additional file 3: Figure S2) with the notable exception of GGA16, W and of linkage group E64, for which the assembly is still incomplete. Due mostly to an inaccurate definition of flanking sequences in a subset of markers for the Illumina BeadChip ordering, we obtained reliable genotypes only for 2145 SNPs (Additional file 4: Table S2).

### Linkage map

Of 2145 SNPs, 1479 were positioned on a total of 28 linkage groups. No map could be constructed for chromosomes 16, E64, W and Z. The sex-averaged linkage map spanned a total of 3057 cM and the average marker spacing was 2.1 cM. The span and the average marker spacing for each linkage group are shown in Table 1.

**Table 1:** **Length and average genetic distance between markers for each linkage group**

**Linkage group**	**Number of markers**	**Map length (cM)**	**Average distance between markers (cM)**
CJA1	231	382	1.7
CJA2	140	346	2.5
CJA3	148	313	2.1
CJA4	119	186	1.6
CJA5	88	164	1.9
CJA6	53	86	1.6
CJA7	50	107	2.1
CJA8	59	89	1.5
CJA9	50	80	1.6
CJA10	41	54	1.3
CJA11	35	72	2.1
CJA12	42	91	2.2
CJA13	39	74	1.9
CJA14	34	65	1.9
CJA15	35	100	2.9
CJA16	-	-	-
CJA17	39	72	1.8
CJA18	34	101	3.0
CJA19	27	54	2.0
CJA20	26	69	2.7
CJA21	27	60	2.2
CJA22	17	46	2.7
CJA23	24	63	2.6
CJA24	23	60	2.6
CJA25	13	60	4.6
CJA26	30	70	2.3
CJA27	17	73	4.3
CJA28	25	81	3.2
CJE22	13	39	3.0
CJE64	-	-	-
CJW	-	-	-
CJZ	-	-	-

### Comparison with chicken

As the markers used in the construction of the quail genetic map were also physically positioned on the chicken genomic map (NCBI, Annotation Release 102), comparisons could be made between the two maps. The marker order was mostly conserved between the two species, although chromosomal rearrangements were observed (as illustrated in Additional file 5: Figure S3). Sets of markers were in an inverted order on CJA1 (from 25 cM to 42 cM and from 133 cM to 147 cM), CJA2 (from 141 cM to 235 cM), CJA3 (from 13 cM to 26 cM), CJA6 (from 5 cM to 17 cM), CJA7 (from 58 cM to 79 cM and from 96 to 109 cM), CJA8 (From 14 cM to 20 cM), CJA9 (from 0 cM to 1 cM), CJA11 (From 0 cM to 1 cM), CJA13 (from 23 to 56 cM), CJA18 (from 30 cM to 97 cM), CJA20 (from 0 to 0.5 cM and from 31 cM to 38 cM) and CJA25 (from 28 to 30 cM). Displaced markers were occasionally observed, positioning them to very different loci than those found on the chicken map. Such events were noted on CJA3 (0 to 2 cM), CJA5 (29 cM to 38 cM), CJA7 (0 cM to 19 cM) and CJA22 (0 to 2 cM).

### QTL detection

Descriptive statistics of the traits analyzed are provided in Table 2. QTLs detected either by the “Sire” model or the “Sire plus Dam” model are shown in Table 3 and in Additional file 6: Figure S4. Eleven genome-wide significant QTLs, and 34 chromosome-wide significant QTLs were identified for behavioral traits (23 QTLs) or production traits (22 QTLs). The average substitution effects (expressed as a percentage of standard deviation) were moderate to high, and ranged between 0.19 (HeadNO on CJA8) and 0.49 (HeadNO CJA1) for behavioral traits and between 0.21 (W17 on CJA1) and 0.53 (WEgg on CJA3) for production traits.

**Table 2:** **Descriptive statistics of traits studied in QTL detection**

	**DistSR (AU)**	**DistIso (cm)**	**TI (s)**	**HeadNO**	**Mount**	**AgrP**	**GentleP**	**W17 (g)**	**W65 (g)**	**AFEgg (day)**	**NEgg**	**WEgg (g)**
Number of Birds	731	720	728	726	335	353	353	730	711	355	347	350
Minimum	0	5.4	0	0	0	0	0	74	165	48	73	9.7
Maximum	2126	3,961	135	12	23	12	11	131	325	72	174	15.3
Mean	487.5	1,168	31.8	1.7	6.7	4.8	1.2	100.2	226.1	56.4	143.1	12.6
Standard deviation	495.6	705.7	20.9	2.9	6.5	4.9	2.1	8.2	29.6	5.0	18.4	1.0

DistSR: Distance travelled on the treadmill in the social reinstatement behavior test; DistIso: Distance travelled at periphery in the social isolation test; TI: Time spent immobile in the tonic immobility test; HeadNO: Number of scans when the quail passed its head through the wire at the front of the cage in the novel object test; Mount: Number of mounts in the sexual motivation test; AgrP: Number of aggressive pecks in the aggressive behavior test; GentleP: Number of gentle pecks in the aggressive behavior test; W17: Body weight at 17 days; W65: Body weight at 65 days; AFEgg: Age at first egg; NEgg: Number of eggs laid; WEgg: Mean egg weight.

**Table 3:** **QTL for social, emotional and production traits in the F2 population**

**Chromosome**	**Traits**	**Position (cM)**	**Nearest markers**	**Confidence interval (cM)**	**Flanking markers**	**Model**	**Level of significance** ^**(1)**^	**QTL effect** ^**(2)**^	**Sires** ^**(3)**^	**Dams**
CJA1	HeadNO	132	snp_soc_3409-snp_soc_3085	131-133	snp_soc_3409-snp_soc_3085	S + D	P < 0.01^†^	0.49[0.15; 1.71]	2	5
	WEgg	193	snp_soc_3301-snp_soc_3547	179-203	snp_soc_3733-snp_soc_4023	S	P < 0.01	0.28[0.20; 0.49]	5	-
	AgrP	236	snp_soc_3475-snp_soc_2139	224-245	snp_soc_4684-snp_soc_2782	S	P = 0.025	0.40[0.28; 0.69]	3	-
	DistSR	281	snp_soc_4666-snp_soc_2881	278-285	snp_soc_4127-snp_soc_4345	S + D	P = 0.037	0.31[0.18; 0.48]	3	9
	W65	317	snp_soc_3213-snp_soc_4360	316-319	snp_soc_3481-snp_soc_3247	S + D	P < 0.01^†^	0.51[0.17; 1.512]	3	9
	W17	319	snp_soc_4529-snp_soc_3247	316-328	snp_soc_3481-snp_FSIEL0M01BAIJK_30	S	P < 0.01^†^	0.21[0.10; 0.46]	5	-
	W17	325	snp_F3SDQUK02BW99J_142-snp_soc_4532	323-325	snp_soc_1488-snp_FJXLD7H02JJLTX-0_66	S + D	P < 0.01^†^	0.39[0.15; 0.79]	5	9
CJA2	AgrP	141	snp_soc_0368-snp_soc_0890	131-153	snp_FSIEL0M02D0BGS_129-snp_soc_2344	S	P = 0.039	0.28[0.16; 0.39]	5	-
	DistSR	149	snp_soc_1978-snp_soc_3824	146-155	snp_soc_4483-snp_soc_4405	S + D	P = 0.043	0.36[0.16; 0.65]	3	6
	DistSR	155	snp_soc_4405-snp_soc_3819	152-159	snp_soc_1978-snp_soc_2372	S	P = 0.024	0.22[0.10; 0.36]	4	-
	DistIso	319	snp_soc_3054-snp_soc_0012	302-337	snp_soc_4310-snp_soc_2553	S	P = 0.038	0.21[0.18; 0.26]	4	-
CJA3	WEgg	156	snp_soc_4141-snp_soc_3447	154-158	snp_soc_4141-snp_soc_3447	S + D	P < 0.01^†^	0.53[0.18; 1.49]	5	3
	W65	156	snp_soc_4141-snp_soc_3447	148-161	snp_soc_0755-snp_soc_0929	S	P < 0.01^†^	0.21[0.12; 0.32]	6	-
	NEgg	225	snp_soc_4257-snp_soc_2884	215-229	snp_soc_4257-snp_soc_3740	S	P = 0.025	0.31[0.15; 0.52]	5	-
	AFEgg	302	snp_soc_3800-snp_soc_4350	294-313	snp_soc_4338-snp_soc_3680	S	P = 0.026	0.39[0.27; 0.48]	4	-
CJA4	DistIso	105	snp_soc_2929-snp_soc_3811	100-118	snp_soc_0843-snp_soc_3328	S + D	P < 0.01^†^	0.26[0.16; 0.70]	4	8
CJA5	W17	54	snp_soc_0024-snp_FSIEL0M02D2UBC_47	45-75	snp_soc_1561-snp_soc_2224	S	P < 0.01^†^	0.24[0.10; 0.43]	4	-
	TI	59	snp_soc_3635-snp_soc_2699	38-68	snp_soc_2028-snp_soc_0641	S	P = 0.050	0.25[0.16; 0.34]	3	-
	W65	88	snp_soc_3485-snp_FSIEL0M02DEXEG_146	83-99	snp_soc_0380-snp_soc_3299	S + D	P < 0.01^†^	0.30[0.12; 0.60]	4	9
	W17	90	snp_FSIEL0M02DEXEG_146-snp_ble_0738	87-101	snp_soc_3485-snp_ble_0738	S + D	P < 0.01^†^	0.38[0.11; 0.80]	4	11
	W65	97	snp_soc_3485-snp_FSIEL0M02DEXEG_146	56-115	snp_FSIEL0M02D2UBC_47-snp_FSIEL0M02D7AG8_195	S	P < 0.01^†^	0.26[0.17; 0.38]	4	-
CJA7	DistIso	96	snp_soc_2405-snp_soc_1537	92-99	snp_soc_2405-snp_soc_1826	S + D	P < 0.01	0.29[0.11; 0.56]	4	12
CJA8	W17	41	snp_soc_2116-snp_soc_2490	37-44	snp_soc_0876-snp_soc_1323	S + D	P = 0.021	0.30[0.15; 0.74]	3	10
	W17	56	snp_soc_2933-snp_soc_2335	53-60	snp_soc_2074-snp_soc_1161	S	P = 0.026	0.23[0.19; 0.26]	4	-
	HeadNO	65	snp_soc_1161-snp_FSIEL0M02DE4EL_249	53-69	snp_soc_2074-snp_soc_1987	S	P = 0.024	0.19[0.13; 0.29]	5	-
CJA9	DistSR	42	snp_soc_1270-snp_soc_0268	39-53	snp_soc_0028-snp_soc_0558	S + D	P < 0.01	0.43[0.19; 1.33]	3	7
	DistSR	55	snp_soc_2491-snp_soc_0558	37-60	snp_soc_0028-snp_soc_2117	S	P = 0.016	0.28[0.11; 0.49]	2	-
CJA10	DistIso	11	snp_soc_2706-snp_soc_0126	8-13	snp_FSIEL0M02C42PC_418-snp_FSIEL0M03HDV3S_67	S + D	P = 0.035	0.36[0.10; 0.81]	2	9
	W65	21	snp_soc_0359-snp_soc_2673	17-22	snp_soc_2915-snp_soc_1219	S + D	P = 0.034	0.42[0.13; 1.25]	3	8
	W17	30	snp_soc_0939-snp_FQU5R7M02IBV7P-0	27-31	snp_soc_0968-snp_FQU5R7M02IBV7P-0_110	S + D	P < 0.01	0.37[0.18; 0.65]	1	9
CJA11	TI	1	snp_soc_0708-snp_soc_1139	0-6	snp_soc_0157-snp_soc_2036	S + D	P < 0.01	0.35[0.18; 0.58]	4	11
	TI	5	snp_soc_0003-snp_soc_1569	0-26	snp_soc_0157-snp_soc_1166	S	P = 0.018	0.21[0.15; 0.38]	5	-
	DistSR	47	snp_soc_1642-snp_soc_1220	34-49	snp_soc_1436-snp_FSIEL0M01BMPFH_253	S	P = 0.023	0.21[0.13; 0.34]	4	-
CJA13	DistSR	4	snp_soc_0855-snp_FSIEL0M02D43ZD_185	0-15	snp_soc_1438-snp_soc_1000	S	P = 0.031	0.21[0.14; 0.28]	4	-
CJA15	DistIso	63	snp_soc_1598-snp_ble_0416	60-65	snp_soc_2236-snp_ble_0416	S	P = 0.033	0.31[0.18; 0.52]	3	-
CJA18	NEgg	3	snp_soc_0250-snp_soc_0714	0-22	snp_soc_2064-snp_soc_0279	S + D	P = 0.025	0.45[0.19; 1.41]	4	-
	W65	18	snp_soc_2042-snp_soc_1172	13-26	snp_soc_2085-snp_soc_1907	S + D	P < 0.01	0.33[0.19; 0.50]	3	9
	W65	42	snp_soc_0424-snp_soc_0163	20-50	snp_soc_1172-snp_soc_0772	S	P < 0.01	0.23[0.12; 0.38]	4	-
	WEgg	61	snp_soc_0010-snp_soc_0395	57-75	snp_soc_1648-snp_soc_1792	S	P < 0.01	0.29[0.15; 0.51]	5	-
	WEgg	62	snp_soc_0395-snp_soc_0042	58-73	snp_soc_0010-snp_FZVM8I102DNHYX_129	S + D	P < 0.01	0.34[0.13; 0.79]	5	3
CJA19	AFEgg	4	snp_soc_0193-snp_soc_0715	0-10	snp_soc_1697-snp_soc_0483	S + D	P = 0.029	0.36[0.24; 0.51]	5	4
	DistIso	8	snp_soc_0715-snp_soc_0483	6-16	snp_soc_0193-snp_soc_1227	S + D	P = 0.020	0.29[0.14; 0.50]	4	9
	DistIso	14	snp_soc_0396-snp_soc_1227	0-20	snp_soc_1697-snp_soc_1227	S	P = 0.039	0.23[0.10; 0.29]	4	-
CJA23	HeadNO	57	snp_soc_1394-snp_ble_033	45-63	snp_soc_1286-snp_FSIEL0M02EPU6F_133	S	P < 0.01	0.20[0.14; 0.30]	5	-
CJA26	DistSR	21	snp_FQU5R7M02I6ZE8-0_225-snp_soc_1180	19-27	snp_soc_1867-snp_soc_0288	S	P = 0.025	0.30[0.15; 0.46]	3	-

^(1)^Level of significance of the QTL at the chromosome level; ^†^indicates that the QTL is genome-wide significant; ^(2)^The effect of the QTL was estimated as the average of the significant parent’s effects (P < 0.05) and expressed in phenotypic standard deviation. The minimum and the maximum effects are given between square brackets; ^(3)^Number of significant parents.

Fourteen QTLs appeared to be common between the Sire and the Sire-Dam models, i.e. related to the same trait, with close likeliest positions and overlapping confidence intervals. This was the case for W17 on CJA1 with genome-wide significant QTLs detected at 319 cM (Sire) and 325 cM (Sire-Dam) and for W65 on CJA5 with genome-wide significant QTL at 97 cM (Sire) and 88 cM (Sire-Dam). Other significant chromosome-wide QTLs were detected by the two models for DistSR on CJA2 (at 155 and 149 cM, for the Sire and Sire-Dam models, respectively) and on CJA9 (at 55 and 42 cM, respectively), for DistIso on CJA19 (at 14 and 8 cM, respectively), for TI on CJA11 (at 5 and 1 cM, respectively) and for WEgg on CJA18 (at 61 and 62 cM, respectively).

Other QTLs were specifically detected by only one of the two models. The Sire model led to one genome-wide significant QTL for W17 on CJA5. Several additional chromosome-wide significant QTLs were detected by this model for behavioral traits such as DistSR on CJA11 (47 cM), CJA13 (4 cM) and CJA26 (21 cM), DistIso on CJA2 (319 cM) and CJA15 (63 cM), TI on CJA5 (59 cM), HeadNO on CJA23 (57 cM), and AgrP on CJA1 (236 cM) and CJA2 (141 cM). Chromosome-wide significant QTLs were specifically detected by the Sire model for production traits such as W17 on CJA8 (56 cM), W65 on CJA18 (42 cM), AFEgg on CJA3 (302 cM), NEgg on CJA3 (225 cM), and WEgg on CJA1 (193 cM). The Sire-Dam model identified five supplementary genome-wide significant QTLs for HeadNO (132 cM) and W65 (317 cM) on CJA1, WEgg (156 cM) on CJA3, DistIso (105 cM) on CJA4, and W17 (90 cM) on CJA5. Chromosome-wide significant QTLs were identified for behavioral traits such as DistSR on CJA1 (281 cM), and DistIso on CJA7 (96 cM) and CJA10 (11 cM). Chromosome-wide significant QTLs were also detected for production traits such as W17 on CJA8 (41 cM) and CJA10 (30 cM), W65 on CJA10 (21 cM) and CJA18 (18 cM), AFEgg on CJA19 (4 cM) and NEgg on CJA18 (3 cM).

## Discussion

Quail show social behaviors close to those observed in the chicken. Moreover, Japanese Quail and chickens have the same number of chromosomes despite showing several morphological differences [39]. As such, the Japanese Quail can be considered as a reliable model for the chicken in the field of genetics [40]. However, a previous study by Inoue-Murayama et al. [41] showed that genetic markers used in the genetic mapping for the chicken were ineffective in Japanese Quail, which highlighted the need to develop quail-specific markers. Consequently several maps were developed using AFLP markers [18], microsatellite markers [19] and both [20]. Comparisons between the information from the quail genetic map and from the assembled chicken sequence showed that the macrochromosomes of the two species had a highly conserved synteny [42]. Similarly, cytogenetic comparisons by Shibusawa et al. [43], showed conservation of the chromosomal organization between the two species. Nevertheless, Kayang et al. [42] observed rearrangements on CJA1, CJA2 and CJA5 and Shibusawa et al. [43] on CJA1, CJA2, CJA4 and CJA8, although the inverted regions on CJA1 and CJA2 were not the same in the two studies. FISH analyses performed by Kayang et al. [42] confirmed the results of Shibusawa et al. [43] on CJA1 and CJA4. Moreover, a study by Sasazaki et al. [44] comparing the positions of orthologous genes between the two species showed a chromosomal rearrangement on CJA2 (in a similar position to that of Shibusawa et al. [43]) and on CJA5 (in a similar position to that of Kayang et al. [42]). It also showed new rearrangements on CJA3 and CJA7. Interestingly, our results were consistent with those of Shibusawa et al. [43] for CJA1, CJA2 and CJA8 and of Sasazaki et al. [44] for CJA3. We also confirmed the inverted region on CJA5 reported by Kayang et al. [42] and Sasazaki et al. [44]. Surprisingly, we did not observe the inverted region described by Shibusawa et al. [43] and Kayang et al. [42] on CJA4. This might have originated from a lack of information from the markers present in the region, preventing identification of the chromosomal rearrangement. We also observed an inverted region on CJA6 never previously reported. Findings by Shibusawa et al. [43] seem to indicate a lack of cytogenetic markers in the rearranged region, which could explain the impossibility of confirming the existence of chromosomal rearrangement on CJA6 by cytogenetic means. In conclusion, the map constructed for this study should accurately represent the genetic organization of the quail genome. With the exception of a few regions, the marker order was conserved between the Japanese Quail and the chicken, which confirmed conserved synteny and supported the relevance of the Japanese Quail as a model for the chicken.

In order to confirm the similarity between QTLs observed in the quail and the chicken, we used the chicken QTL Database [36,37]. We first undertook these comparisons on widely studied traits such as body weight. In the present study, we detected several QTLs for body weight at a juvenile age (W17) and at a sexually mature age (W65). Interestingly, QTLs controlling these two traits co-localized on CJA1 (316 to 328 cM) and CJA5 (56 to 115 cM), suggesting the existence of regions controlling the overall growth of the animal. As shown in Additional file 1: Table S1, growth-related QTLs were discovered in the homologous regions of the chicken by several studies at a juvenile age but more rarely at a mature age. Similarly, body weight QTLs in the chicken were found in the homologous regions of W65 QTL on CJA3 (148–165 cM) and W17 QTL on CJA8 (37–60 cM). These results showed that several QTLs were consistent for similar traits between the chicken and the quail, although we do not have any indication that they are governed by common genes. By contrast, egg trait-related QTLs were rarely identified in the chicken. Only one QTL for WEgg on CJA3 (52.6 to 56.7 Mb) showed coordinates overlapping with an egg weight QTL detected on GGA3 (49.0 to 81.4 Mb) by Tuiskula-Haavisto et al. [45]. One hypothesis is that the genetic determinism of egg production traits is not as conserved between the quail and the chicken as the determinism of body weight; our population may also lack the genetic variability in the QTL regions identified in the chicken. Another explanation may be that our experimental scheme was less powerful for the egg production traits, which were measured in only half of the birds. Interestingly, our results indicated the existence of putative pleiotropic loci affecting both weight and egg traits. This was the case on CJA3 where a WEgg QTL co-localized with a W65 QTL. Physiologically, it has been shown that the average egg weight roughly represents 8% of the female bodyweight in the quail and 3% in the chicken [46]. This candidate region could underlie the genetic correlation already observed in the quail between these two traits [47-50]. The W65 QTL and NEgg QTL identified on CJA18 suggested another candidate region which might partly explain the unfavorable genetic correlation in the quail between these two traits reported by Silva et al. [48].

As only a few studies have focused on the detection of behavioral QTL in either the quail [11,12] or the chicken [10,13-15,51-53], comparison with previous studies is less comprehensive than for production traits. Interestingly, the AgrP QTL on CJA1 corresponded to the homologous region (around 109 Mb to 127 Mb) in the chicken harboring a QTL controlling the number of feather pecks an individual received [14]. The effect of this locus was recently confirmed by Biscarini et al. [51] who showed a significant effect of SNP rs1530785 on the number of pecks received on the dorsal and ventral parts of the body. This behavioral QTL was shown to contain the gene coding for the isoenzyme MAOA (Mono-Amine Oxidase A), while two studies showed increased aggressive behavior in *MAOA* knockout mice [54,55]. Indeed, MAOA regulates the concentration of several substrates such as serotonin, dopamine and the norepinephrine [56]. One of these substrates, serotonin, has been shown to affect several behavioral processes such as cooperation between individuals, hierarchy establishment and aggressiveness (see [57] for review). Furthermore, the study by van Hierden et al. [58] demonstrated that lines of chicken divergently selected for their feather pecking propensity showed a different serotonin turnover with a slower degradation of serotonin in the HFP (High Feather Pecking) than in the LFP lines (Low Feather Pecking). Moreover, Wysocki et al. [59] studied *MAOA* expression in brain tissue of HFP and LFP lines and suggested that it could possibly be downgraded in HFP. However, the observation on micro-arrays could not be confirmed by qPCR. Further research is needed to investigate the variability of this gene in the HSR and LSR lines, which also differed in terms of aggressiveness [60,61]. Another QTL for AgrP (46.6 Mb) was observed on CJA2 which co-localized with a QTL for the number of severe feather pecks given by a chick (46.0 to 51.3 Mb) identified by Buitenhuis et al. [15]. When considering the extent of our confidence interval, it seems that we identified a more refined region than that described by Buitenhuis et al. [15]. In addition, the chromosomal rearrangement we observed in the quail brought the AgrP-QTL and DistSR-QTL observed on CJA2 closer, suggesting a possible pleiotropic effect of this region. The coordinates of the DistSR QTL (79.2 to 79.4 Mb and 81.1 to 81.4 Mb for the Sire and Sire-Dam models, respectively) co-localized with a suggestive QTL for the number of Gentle Feather Pecks given by an individual (67 to 85 Mb) identified by Buitenhuis et al. [15]. Previous reports on the HSR and LSR lines showed that, in cases of group disruption, HSR birds manifested increased explorative behavior of unfamiliar conspecifics such as gentle peckings [62], which reinforces this QTL co-localization.

Interestingly, the TI-QTL on CJA11 showed coordinates (3.3 to 3.7 Mb and 0.5 to 2.8 Mb for the Sire and Sire-Dam models, respectively) corresponding to a QTL controlling the frequency of defecation in an Open-Field (0.5 to 5.0 Mb) identified by Buitenhuis et al. [13] in the chicken. These co-localizations suggest an effect of this region on some aspects of emotional reactivity of an animal as the duration of tonic immobility after restraint and the frequency of defecation in Open-Field are both behavioral responses expressed during stressful events [63,64]. Our results also highlighted putative pleiotropic regions controlling emotional reactivity and body-weight. This was the case for CJA5 for a region (spanning 38 to 75 cM) which harbored a genome-wide significant QTL for W17 and a chromosome-wide significant QTL for TI. It was also the case for CJA8 where QTLs for HeadNO and W17 overlapped. A genetic link between a bird’s growth and its emotional reactivity or sociability has already been evidenced by studying the *Growth 1* region in the chicken [10,17] and several candidate genes affecting the sociability or the emotional reactivity in this region have been suggested (*AVRP1, AVRP2, NRCAM*) by Wiren et al. [65]. A pleiotropic role of the *Growth 2* region [16] was also suggested as it co-localized with a QTL controlling the reaction to a novel object [10]. We also identified a putative pleiotropic QTL for AFEgg and DistIso on CJA19. Moreover, it is interesting to note that DistSR QTL on CJA13 had overlapping coordinates (1.6 to 1.8 Mb) with an AFEgg QTL (0.6 to 16.0 Mb) identified by Podisi et al. [66] on GGA13, although the latter is very wide. An unfavorable correlation between growth and fearfulness has already been reported in poultry, as in this F2 experimental design [9,67]. At the same time, favorable genetic relationships between sociability or emotional reactivity and egg traits (age at first egg and number of eggs) have been reported [9]. As the confidence intervals of our QTLs remain large, further research will be needed to confirm the pleiotropic effects of the QTL regions we identified, and to look for candidate genes underlying the relationship between production and behavior. Identification of these molecular factors should help to understand whether production can be improved without impairing emotional and social behavior, which remains a vexing question for poultry production.

With a total of 15 QTLs obtained by the two models, the most numerous regions involved the social motivation traits (i.e. DistSR and DistIso), which highlighted the interest of divergent lines selected on social motivation for QTL detection. This study is the first to date to identify social motivation QTL across the whole genome, as previous studies focused either on a selected QTL region [65] or considered composite traits obtained by Principal Component Analysis [53]. The eight regions we identified for DistSR did not co-localize with the seven found for DistIso. This finding is consistent with the low phenotypic and genetic correlations we observed between DistSR and DistIso with the same design [9] and suggests that these two traits are under different genetic control and measure different components of social motivation, probably depending on whether the animals are in visual and auditory contact with conspecifics or not. Although the confidence intervals of the social motivation QTLs were relatively wide and the identification of candidate genes rather speculative at this stage, the use of Ingenuity Pathway Analysis [68] revealed two interesting genes related to behavior establishment. The first (located in the DistSR QTL on CJA2) is the dopa-decarboxylase gene (*DDC*) which encodes the aromatic L-amino acid decarboxylase (AADC). This enzyme allows conversion of L-DOPA to dopamine and L-5 hydroxytryptophan to serotonin. Genetic variations in the *DDC* gene have been reported to be associated with the defective attention and disorders such as Attention Deficit Hyperactivity [69] in humans. The second (located in the DistSR QTL on CJA9) is the serotonin receptor 2B gene (*HTR2B*). Studies in the field of personality traits were recently conducted on *HTR2B. HTR2B* knockout mice showed increased impulsive behavior and novelty seeking [70]. Moreover, an association between *HTR2B* polymorphism and a personality trait of fun seeking has been found in humans [71].

## Conclusions

This study identified several QTL regions for social and emotional behaviors in the quail. Further research will be needed to refine these regions and confirm or refute the role of candidate genes, such as the neurotransmitters genes. Our genetic map confirmed a well conserved synteny while the linkage analysis suggested some putative common QTLs between the quail and the chicken. It is to be hoped that the discovery of genes and polymorphisms related to behavioral traits in the quail will have further applications for the chicken and will contribute to solving the animal welfare issues encountered in poultry production.

## Additional files

Additional file 1: Table S1. QTL physical comparisons between the quail and the chicken. Results from comparison between QTLs identified in the current analysis in the quail and those previously observed in the chicken at the corresponding physical coordinates assuming conserved synteny. Additional file 2: Figure S1. Chicken genome coverage (%). Coverage by the SNP markers of the chicken genome. Additional file 3: Figure S2. Chicken genetic map coverage in SNP/cM for each chromosome. Density of the SNP markers for each chromosome in the chicken genetic map. Additional file 4: Table S2. List of the SNPs genotyped on the experimental design. Names and accession numbers of the 2145 SNP markers used for building the genetic map. All the SNPs are available in dbSNP (http://www.ncbi.nlm.nih.gov/SNP/). Additional file 5: Figure S3. Comparison between the quail genetic map and the chicken physical map. Comparison between organization of the SNP markers in the genetic map of the quail and in the physical map of the chicken. Additional file 6: Figure S4. Quail genetic map with positioned QTL. Graphic representation of the quail genetic map constructed with SNP markers. Longer chromosomes are shown in different parts; number is given in brackets. QTL identified were positioned on the map in colored boxes, with pink for social motivation-related QTL, red for emotional reactivity QTL, blue for aggressiveness QTL, and green for production QTL. Genome-wide significant QTL are represented in filled boxes and chromosome-wide significant QTL in shaded boxes.
