# Supplementary material for: A medium density genetic map and QTL for behavioral and production traits in Japanese quail
Source: BMC Genomics. 2015 Jan 22;16(1):10. doi: 10.1186/s12864-014-1210-9 (PMC4307178; doi:10.1186/s12864-014-1210-9)
Supplement: Additional file 1: Table S1. — QTL physical comparisons between the quail and the chicken. Results from comparison between QTLs identified in the current analysis in the quail and those previously observed in the chicken at the corresponding physical coordinates assuming conserved synteny. [file 12864_2014_1210_MOESM1_ESM.pdf]

Chromosome	Traits	Position in the Quail map (cM)	Model	Reference giving physical coordinates of putative nearest markers in Chicken map, assuming synteny between both maps.	Reference giving physical coordinates of putative Flanking Markers in Chicken map, assuming synteny between both maps.
CJA1	HeadNO	132	S+D	BW: [1-3]; AFEgg: [4]	
	WEgg	193	S	BW: [5]	
	AgrP	236	S	BW: [1, 6] ; RCFP: [7, 8]	LCall: [9]
	DistSR	281	S+D	BW: [1, 2, 6, 10]	
	W65	317	S+D	BW: [2, 3, 11-13]	
	W17	319	S	BW: [2, 3, 10-13]	BW: [14, 15]; NO: [16]
	W17	325	S+D	BW: [2, 3, 10-15]; NO: [16]	BW: [15, 17]
CJA2	AgrP	141	S	FP: [18]	
	DistSR	149	S+D	BW: [2, 19]; GentleP: [18]	
	DistSR	155	S	BW: [2, 19]	
	DistIso	319	S	BW: [20]	BW: [1, 4]
CJA3	WEgg	156	S+D	BW: [10, 12, 20, 21]; AFEgg: [4]; WEGG: [5]	
	W65	156	S	BW: [10, 12, 20, 21]; AFEgg: [4]; WEGG: [5]	
	NEgg	225	S	BW: [1, 20]	
	AFEgg	302	S		
CJA4	DistIso	105	S+D	BW: [2]	WEgg: [22-24]
CJA5	W17	54	S	BW: [10, 24]	
	TI	59	S	BW:[10, 11, 20]; WEgg: [23]	
	W65	88	S+D	BW: [10, 12, 25]	
	W17	90	S+D	BW: [10, 20, 25]	BW: [12]
	W65	97	S	BW: [20, 25]	
CJA7	DistIso	96	S+D		
CJA8	W17	41	S+D	BW: [3, 11]	
	W17	56	S	BW: [1, 3]	
	HeadNO	65	S	BW: [1, 3]	
CJA9	DistSR	42	S+D	BW: [26]	
	DistSR	55	S		BW: [26]
CJA10	DistIso	11	S+D		
	W65	21	S+D		
	W17	30	S+D		
CJA11	TI	1	S+D	BW: [1]; FreqDef: [9]	
	TI	5	S	FreqDef: [9]	BW: [1, 27]
	DistSR	47	S		BW: [10]
CJA13	DistSR	4	S	BW-AFEgg: [4]	
CJA15	DistIso	63	S		

CJA18	NEgg	3	S+D	BW: [19]	
	W65	18	S+D		
	W65	42	S		
	WEgg	61	S		
	WEgg	62	S+D		
CJA19	AFEgg	4	S+D		
	DistIso	8	S+D		
	DistIso	14	S		
CJA23	HeadNO	57	S		
CJA26	DistSR	21	S		

Additional Table 1 – QTL physical comparisons between Quail and Chicken.

Legend: **BW**: QTL linked to body-weight traits; **AFEgg**: QTL linked to the Age at First Egg; **WEgg**: QTL linked to egg-weight traits; **FreqDef**: QTL linked to the frequency of defecation during an Open-Field test; **LCall**: Latency to emit calls during an Open-Field test; **LTW**: QTL linked to the latency to walk during an Open-Field Test; **RCFP**: QTL linked to receiving feather pecks; **FP**: QTL linked to the number of feather pecks given; **GentleP**: QTL linked to the number of gentle feather pecks given ; **NO**: QTL linked to the reaction at a novel object.

References

1. Podisi BK, Knott SA, Burt DW, Hocking PM: **Comparative analysis of quantitative trait loci for body weight, growth rate and growth curve parameters from 3 to 72 weeks of age in female chickens of a broiler-layer cross.** *BMC Genet* 2013, **14:**11.
2. Carlborg R, Hocking PM, Burt DW, Haley CS: **Simultaneous mapping of epistatic QTL in chickens reveals clusters of QTL pairs with similar genetic effects on growth.** *Genet Res* 2004, **83:**197-209.
3. SWEggalem A, Morrice DM, Law A, Windsor D, Haley CS, Ikeobi CON, Burt DW, Hocking PM: **Mapping of quantitative trait loci for body weight at three, six, and nine weeks of age in a broiler layer cross.** *Poultry Science* 2002, **81:**1775-1781.
4. Podisi BK, Knott SA, Dunn IC, Law AS, Burt DW, Hocking PM: **Overlap of quantitative trait loci for early growth rate, and for body weight and age at onset of sexual maturity in chickens.** *Reproduction* 2011, **141:**381-389.
5. Tuiskula-Haavisto M, De Koning DJ, Honkatukia M, Schulman NF, Maki-Tanila A, Vilkki J: **Quantitative trait loci with parent-of-origin effects in chicken.** *Genet Res* 2004, **84:**57-66.
6. Rao Y, Shen X, Xia M, Luo C, Nie Q, Zhang D, Zhang X: **SNP mapping of QTL affecting growth and fatness on chicken GGA1.** *Genetics Selection Evolution* 2007, **39:**569-582.
7. Buitenhuis AJ, Rodenburg TB, Siwek M, Cornelissen SJB, Nieuwland MGB, Crooijmans R, Groenen MAM, Koene P, Bovenhuis H, van der Poel JJ: **Identification of quantitative trait loci for receiving pecks in young and adult laying hens.** *Poultry Science* 2003, **82:**1661-1667.
8. Biscarini F, Bovenhuis H, van Arendonk JAM, Parmentier HK, Jungerius AP, van der Poel JJ: **Across-line SNP association study of innate and adaptive immune response in laying hens.** *Animal Genetics* 2010, **41:**26-38.
9. Buitenhuis AJ, Rodenburg TB, Siwek M, Cornelissen SJB, Nieuwland MGB, Crooijmans R, Groenen MAM, Koene P, Bovenhuis H, van der Poel JJ: **Identification of QTLs involved in open-field behavior in young and adult laying hens.** *Behav Genet* 2004, **34:**325-333.
10. Carlborg O, Kerje S, Schutz K, Jacobsson L, Jensen P, Andersson L: **A global search reveals epistatic interaction between QTL for early growth in the chicken.** *Genome Res* 2003, **13:**413-421.
11. Kerje S, Carlborg O, Jacobsson L, Schutz K, Hartmann C, Jensen P, Andersson L: **The twofold difference in adult size between the red junglefowl and White Leghorn chickens is largely explained by a limited number of QTLs.** *Animal Genetics* 2003, **34:**264-274.
12. Jacobsson L, Park HB, Wahlberg P, Fredriksson R, Perez-Enciso M, Siegel PB, Andersson L: **Many QTLs with minor additive effects are associated with a large difference in growth between two selection lines in chickens.** *Genet Res* 2005, **86:**115-125.
13. Liu X, Li H, Wang S, Hu X, Gao Y, Wang Q, Li N, Wang Y, Zhang H: **Mapping quantitative trait loci affecting body weight and abdominal fat weight on chicken chromosome one.** *Poultry Science* 2007, **86:**1084-1089.
14. Wright D, Rubin CJ, Barrio AM, Schutz K, Kerje S, Brandstrom H, Kindmark A, Jensen P, Andersson L: **The genetic architecture of domestication in the chicken: effects of pleiotropy and linkage.** *Mol Ecol* 2010, **19:**5140-5156.

15. Wahlberg P, Carlborg O, Foglio M, Tordoir X, Syvanen AC, Lathrop M, Gut IG, Siegel PB, Andersson L: **Genetic analysis of an F-2 intercross between two chicken lines divergently selected for body-weight.** *Bmc Genomics* 2009, **10:**13.
16. Schütz KE, Kerje S, Jacobsson L, Forkman B, Carlborg O, Andersson L, Jensen P: **Major growth QTLs in fowl are related to fearful behavior: possible genetic links between fear responses and production traits in a red junglefowl x White Leghorn intercross.** *Behav Gen* 2004, **34:**121-130.
17. Zhou H, Deeb N, Evock-Clover CM, Ashwell CM, Lamont SJ: **Genome-wide linkage analysis to identify chromosomal regions affecting phenotypic traits in the chicken. I. Growth and average daily gain.** *Poultry Science* 2006, **85:**1700-1711.
18. Buitenhuis AJ, Rodenburg TB, van Hierden YM, Siwek M, Cornelissen SJB, Nieuwland MGB, Crooijmans R, Groenen MAM, Koene P, Korte SM, et al: **Mapping quantitative trait loci affecting feather pecking behavior and stress response in laying hens.** *Poultry Science* 2003, **82:**1215-1222.
19. Ambo M, Moura A, Ledur MC, Pinto LFB, Baron EE, Ruy DC, Nones K, Campos RLR, Boschiero C, Burt DW, Coutinho LL: **Quantitative trait loci for performance traits in a broiler x layer cross.** *Animal Genetics* 2009, **40:**200-208.
20. Nadaf J, Pitel F, Gilbert H, Duclos MJ, Vignoles F, Beaumont C, Vignal A, Porter TE, Cogburn LA, Aggrey SE, et al: **QTL for several metabolic traits map to loci controlling growth and body composition in an F-2 intercross between high- and low-growth chicken lines.** *Physiol Genomics* 2009, **38:**241-249.
21. Ankra-Badu GA, Le Bihan-Duval E, Mignon-Grasteau S, Pitel F, Beaumont C, Duclos MJ, Simon J, Carre W, Porter TE, Vignal A, et al: **Mapping QTL for growth and shank traits in chickens divergently selected for high or low body weight.** *Animal Genetics* 2010, **41:**400-405.
22. Schreiweis MA, Hester PY, Moody DE: **Identification of quantitative trait loci associated with bone traits and body weight in an F2 resource population of chickens.** *Genetics Selection Evolution* 2005, **37:**677-698.
23. Goraga ZS, Nassar MK, Brockmann GA: **Quantitative trait loci segregating in crosses between NWEgg Hampshire and White Leghorn chicken lines: I. egg production traits.** *Animal Genetics* 2012, **43:**183-189.
24. Sasaki O, Odawara S, Takahashi H, Nirasawa K, Oyamada Y, Yamamoto R, Ishii K, Nagamine Y, Takeda H, Kobayashi E, Furukawa T: **Genetic mapping of quantitative trait loci affecting body weight, egg character and egg production in F2 intercross chickens.** *Animal Genetics* 2004, **35:**188-194.
25. Tercic D, Holcman A, Dovc P, Morrice DR, Burt DW, Hocking PM, Horvat S: **Identification of chromosomal regions associated with growth and carcass traits in an F-3 full sib intercross line originating from a cross of chicken lines divergently selected on body weight.** *Animal Genetics* 2009, 40:743-748.
26. Siwek M, Cornelissen SJB, Buitenhuis AJ, Nieuwland MGB, Bovenhuis H, Crooijmans R, Groenen MAM, Parmentier HK, van der Poel JJ: **Quantitative trait loci for body weight in layers differ from quantitative trait loci specific for antibody responses to sheep red blood cells**. Poultry Science 2004, 83:853-859.
27. Nassar MK, Goraga ZS, Brockmann GA: **Quantitative trait loci segregating in crosses between NWEgg Hampshire and White Leghorn chicken lines: II. Muscle weight and carcass composition**. Animal Genetics 2012, 43:739-745.
