# Supplementary figures and images for: A medium density genetic map and QTL for behavioral and production traits in Japanese quail
Source: BMC Genomics. 2015 Jan 22;16(1):10. doi: 10.1186/s12864-014-1210-9 (PMC4307178; doi:10.1186/s12864-014-1210-9)

**Additional Figure 1:** Chicken Genome coverage (%)

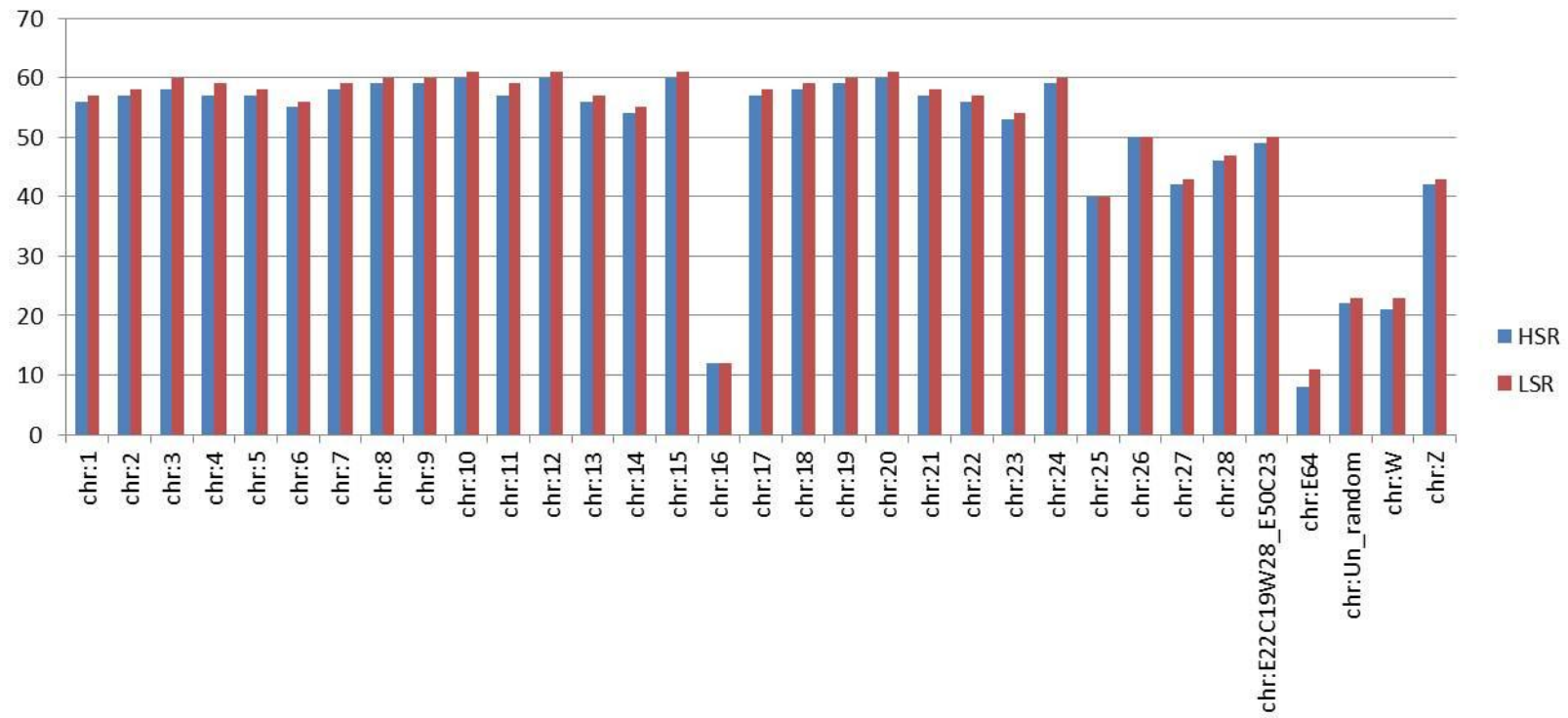

Supplement: Additional file 2: Figure S1. — Chicken genome coverage (%). Coverage by the SNP markers of the chicken genome. [file 12864_2014_1210_MOESM2_ESM.pdf]

**Additional Figure 2:** Chicken genetic map coverage in SNP/cM for each chromosome

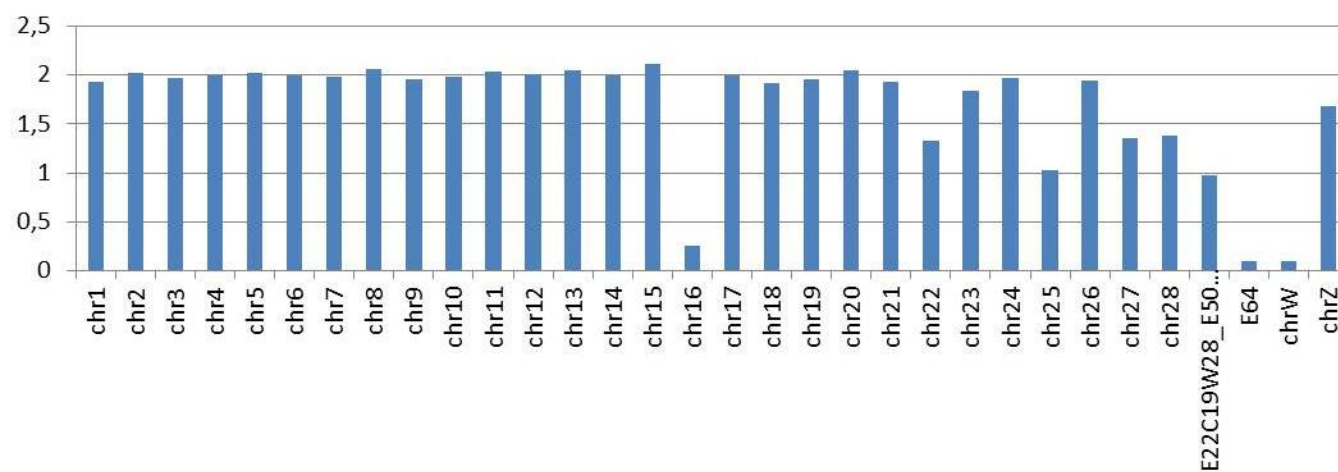

Supplement: Additional file 3: Figure S2. — Chicken genetic map coverage in SNP/cM for each chromosome. Density of the SNP markers for each chromosome in the chicken genetic map. [file 12864_2014_1210_MOESM3_ESM.pdf]
