# Supplementary material for: A medium density genetic map and QTL for behavioral and production traits in Japanese quail
Source: BMC Genomics. 2015 Jan 22;16(1):10. doi: 10.1186/s12864-014-1210-9 (PMC4307178; doi:10.1186/s12864-014-1210-9)
Supplement: Additional file 5: Figure S3. — Comparison between the quail genetic map and the chicken physical map. Comparison between organization of the SNP markers in the genetic map of the quail and in the physical map of the chicken. [file 12864_2014_1210_MOESM5_ESM.pdf]

Chromosome 1

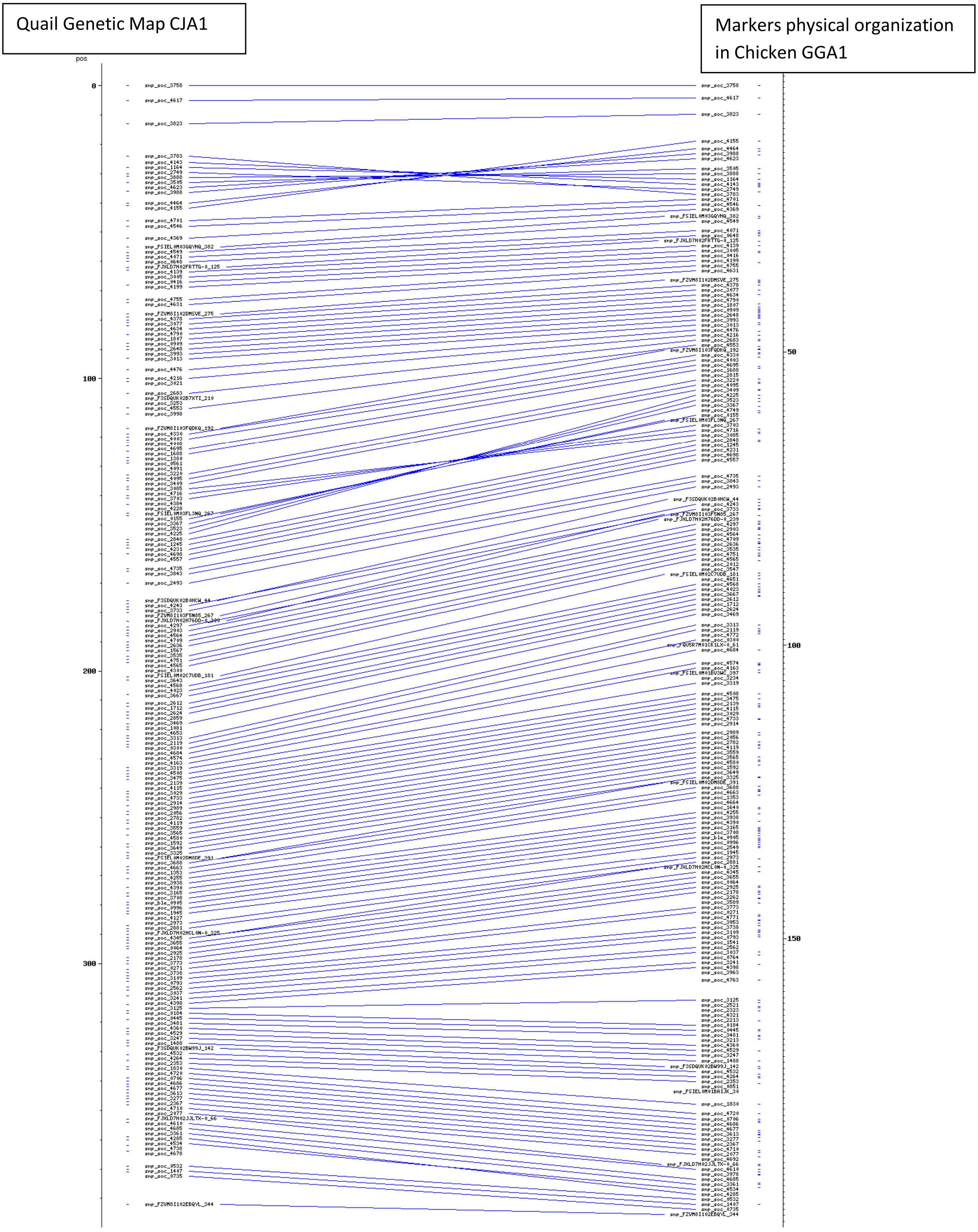

Chromosome 2

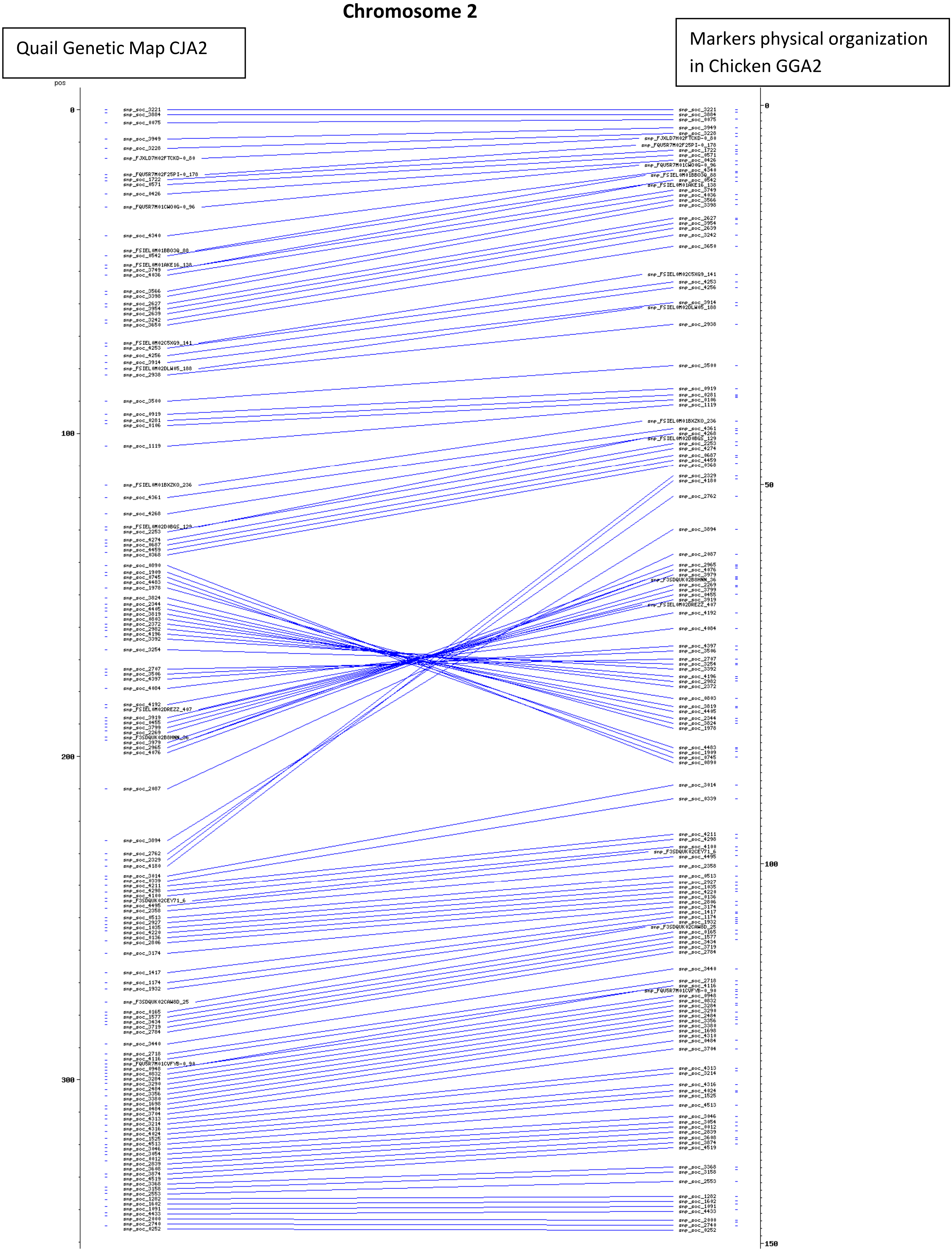

Chromosome 3

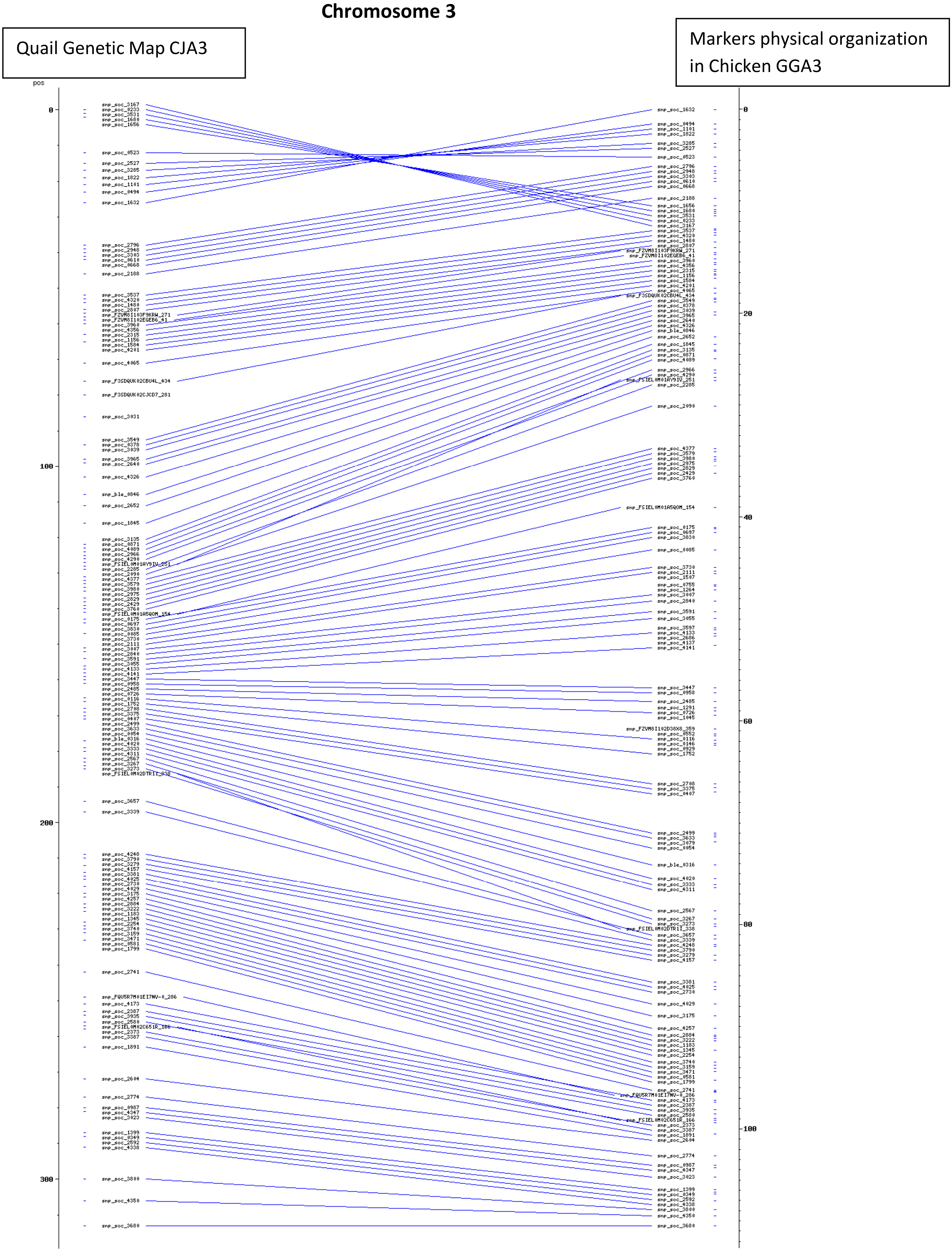

Chromosome 5

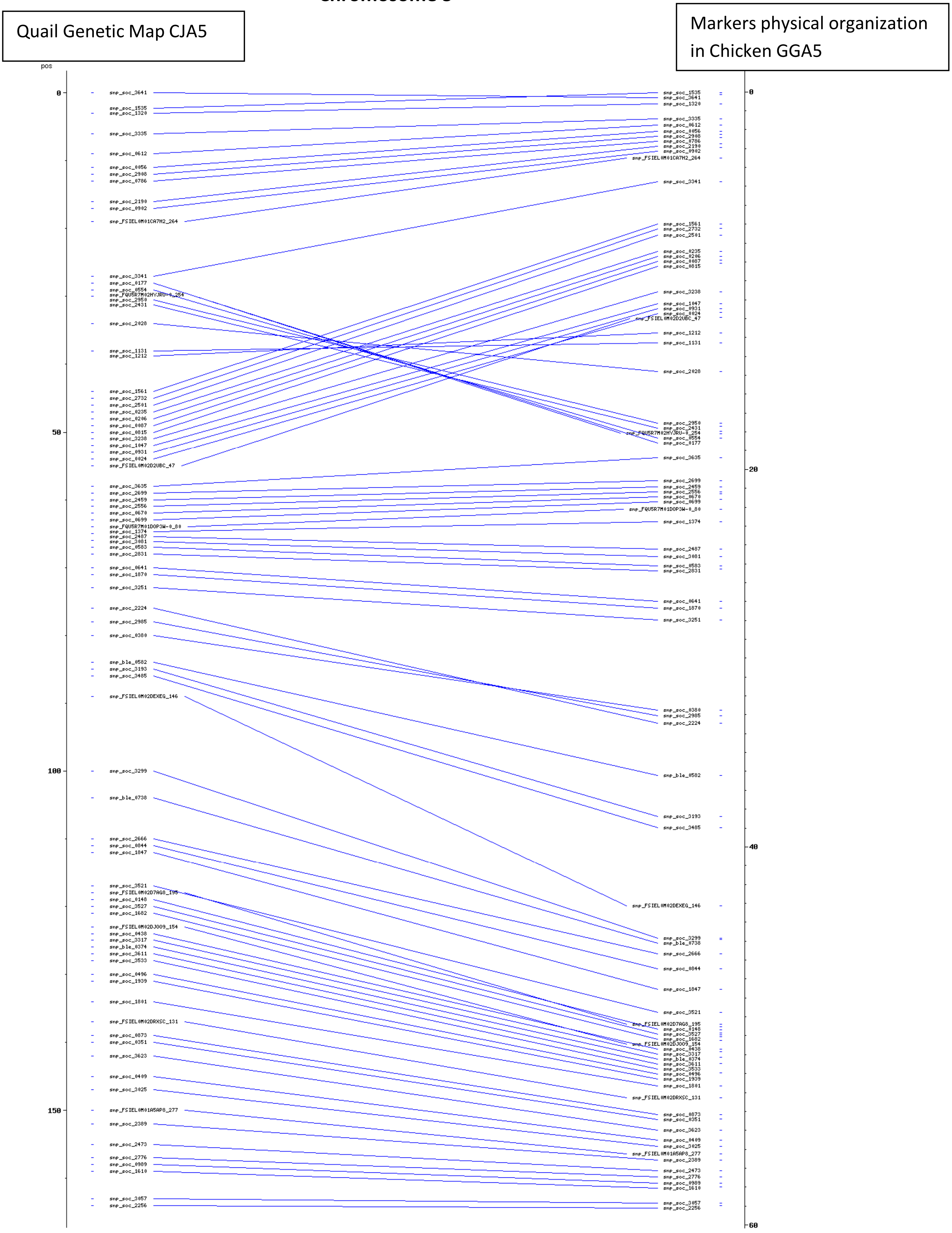

Chromosome 6

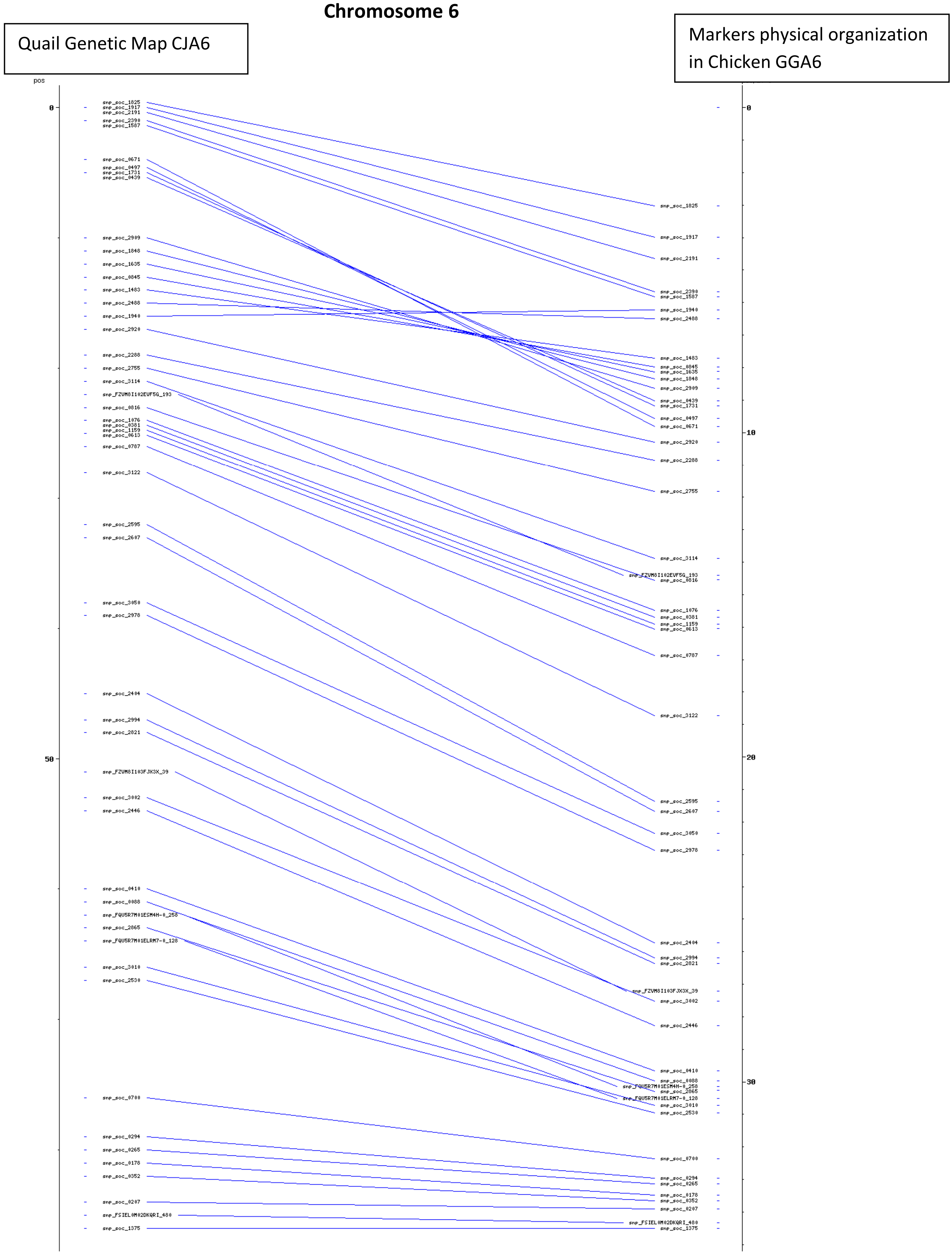

Chromosome 7

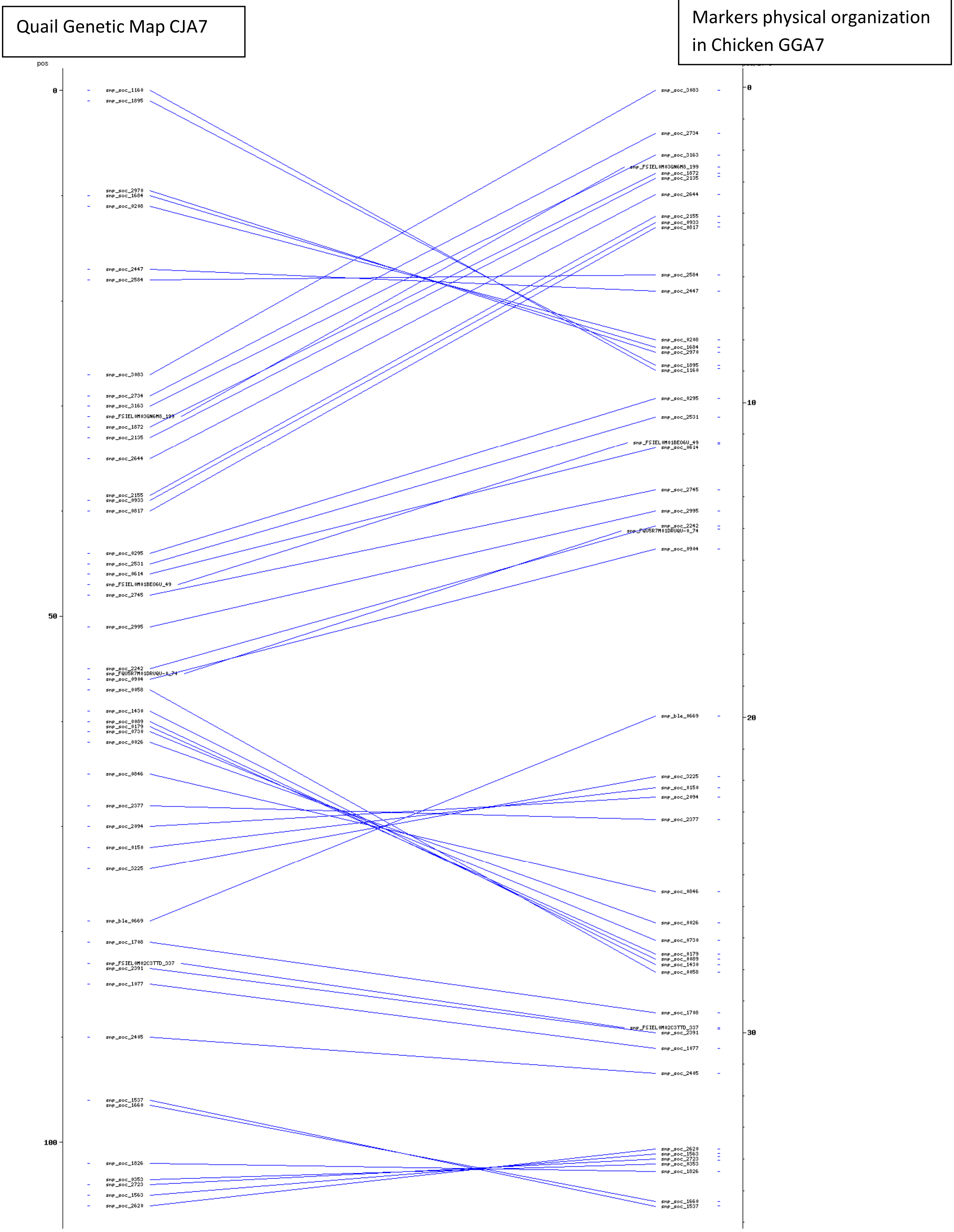

Chromosome 8

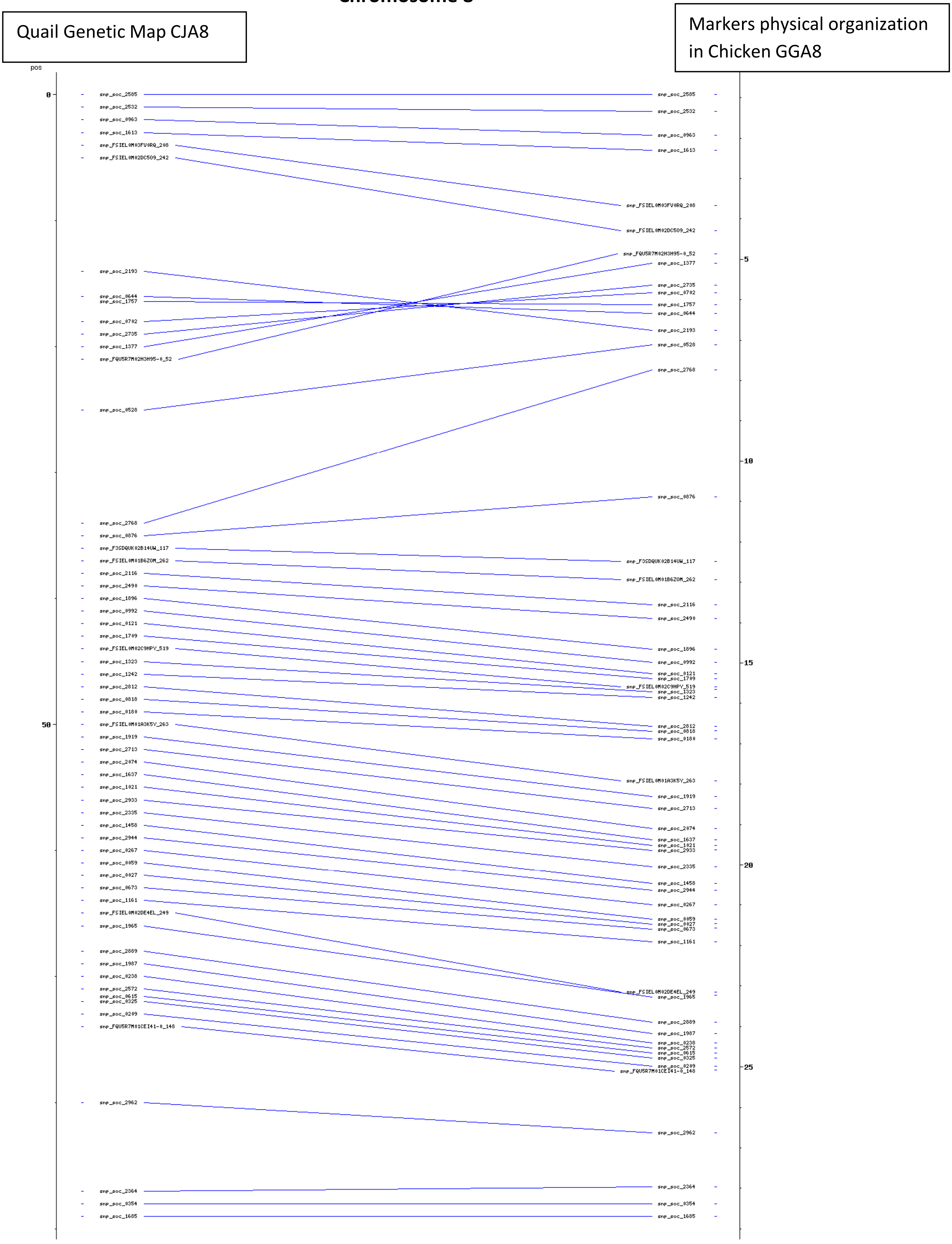

Chromosome 9

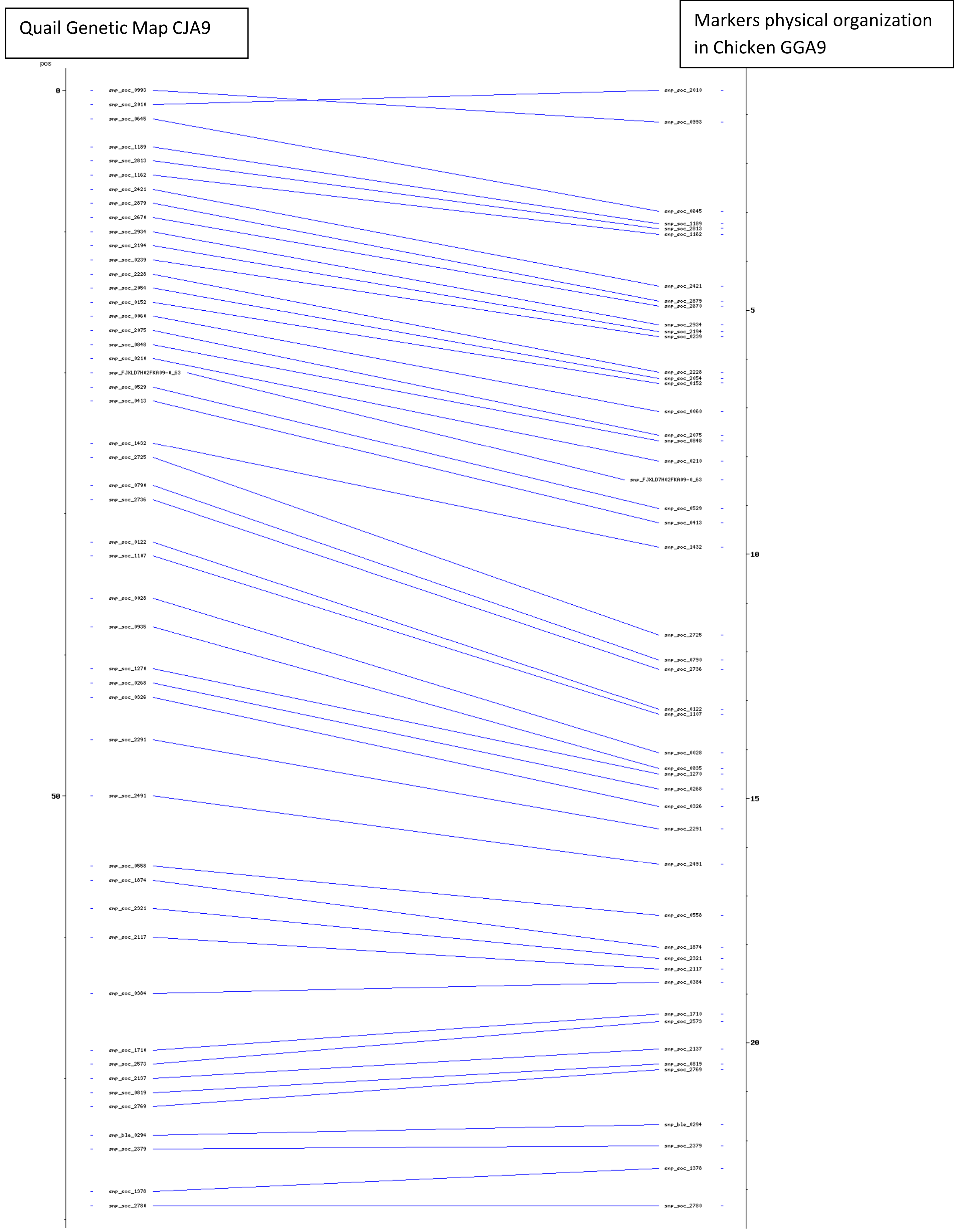

Chromosome 11

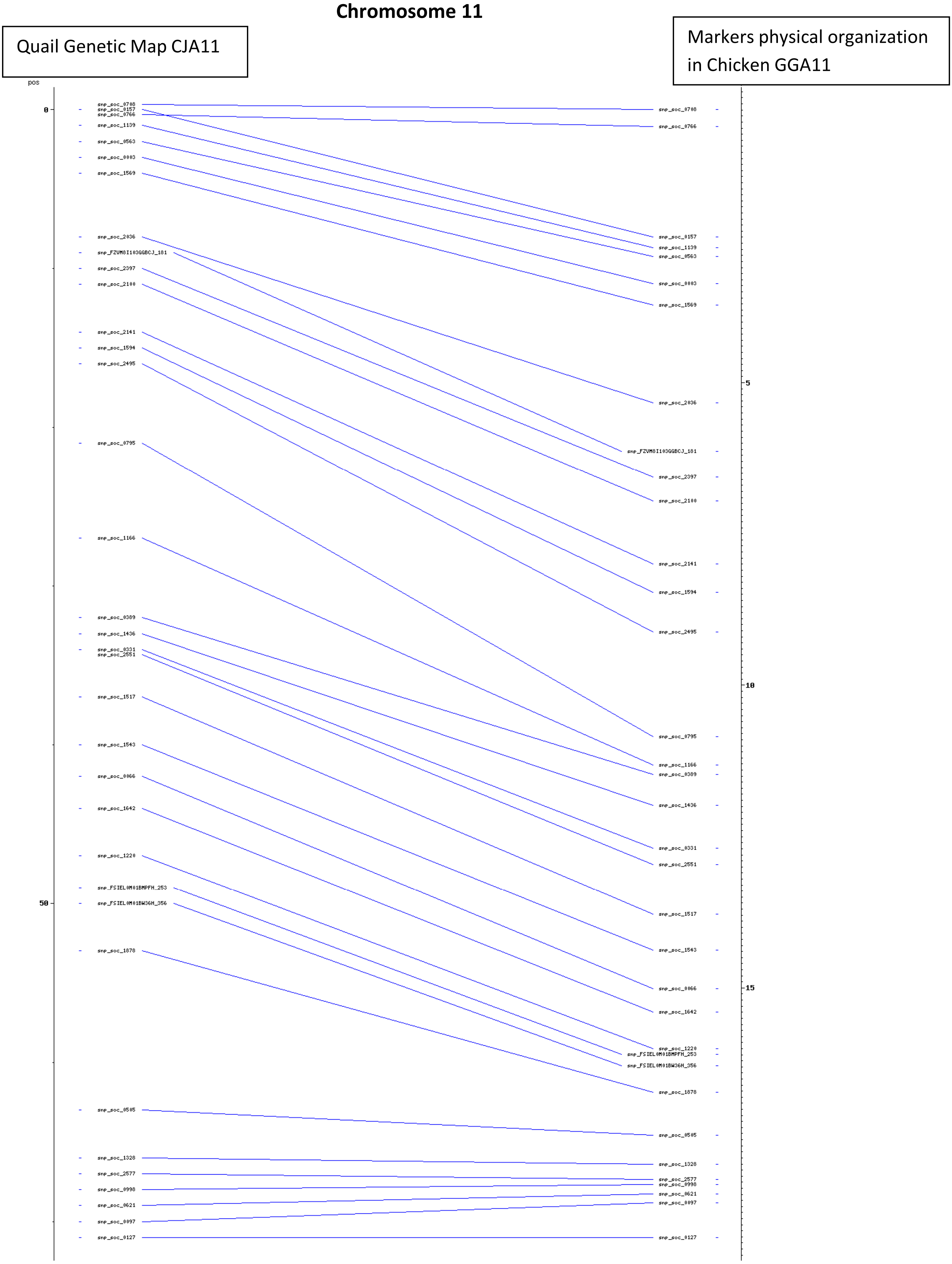

Chromosome 13

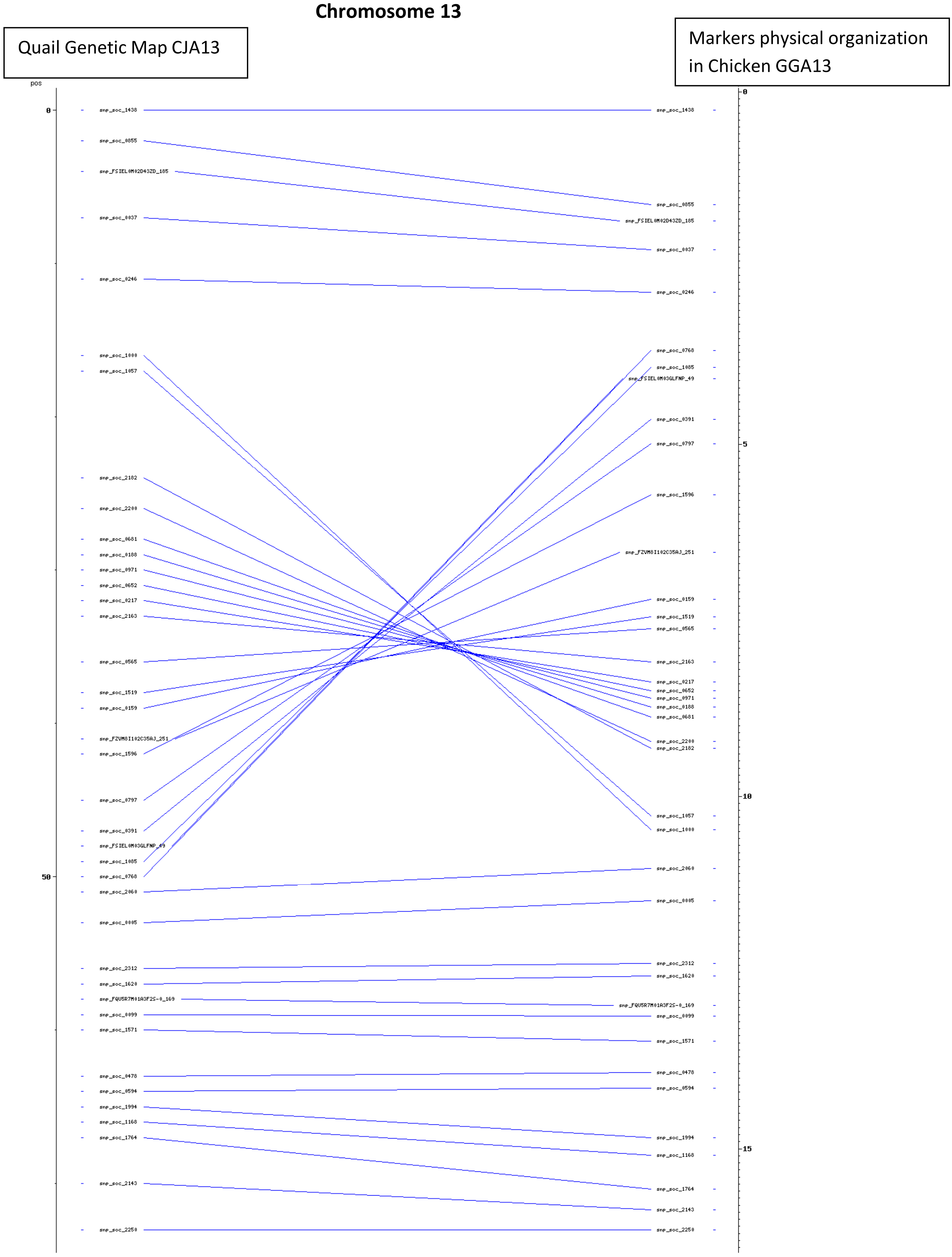

Chromosome 18

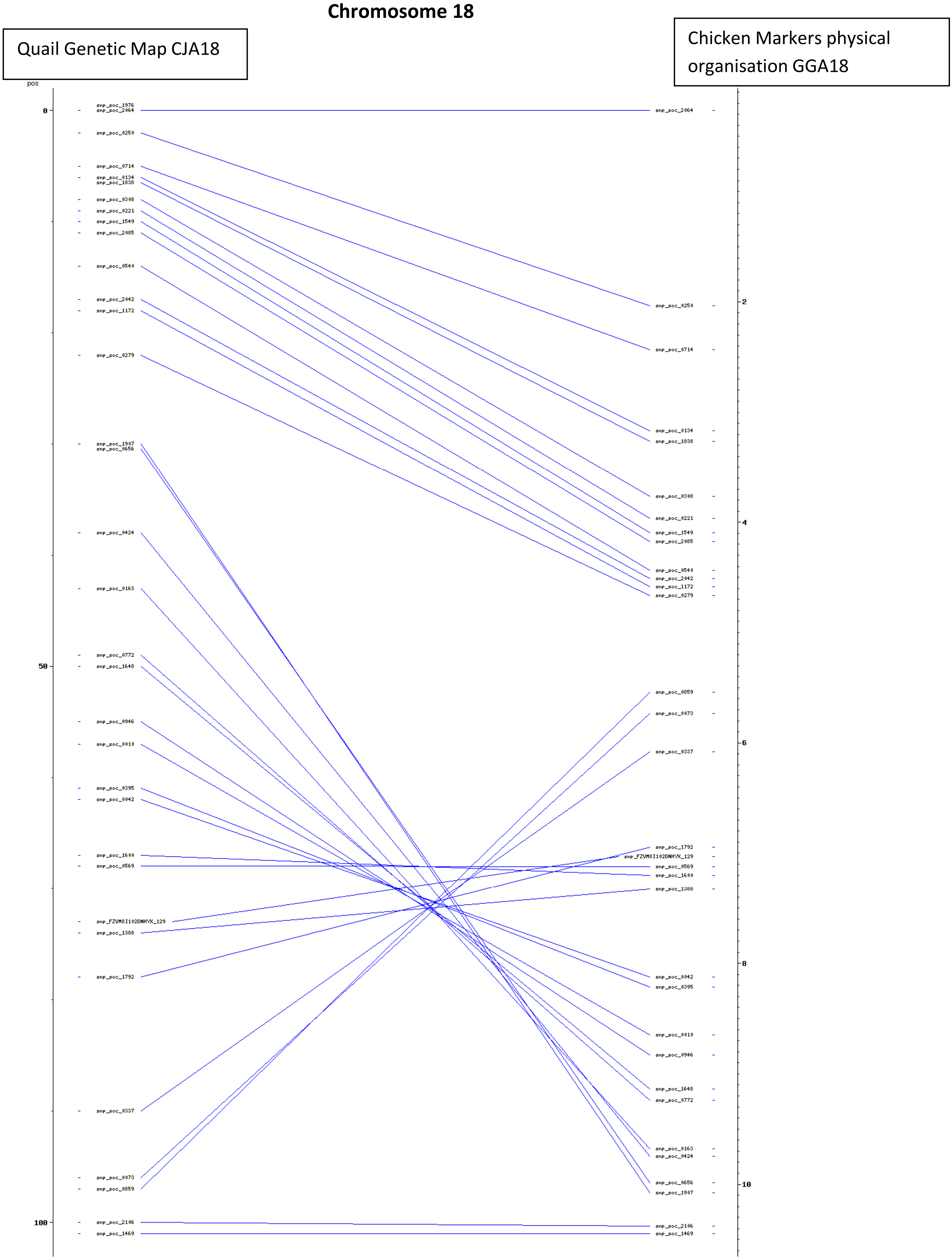

Chromosome 20

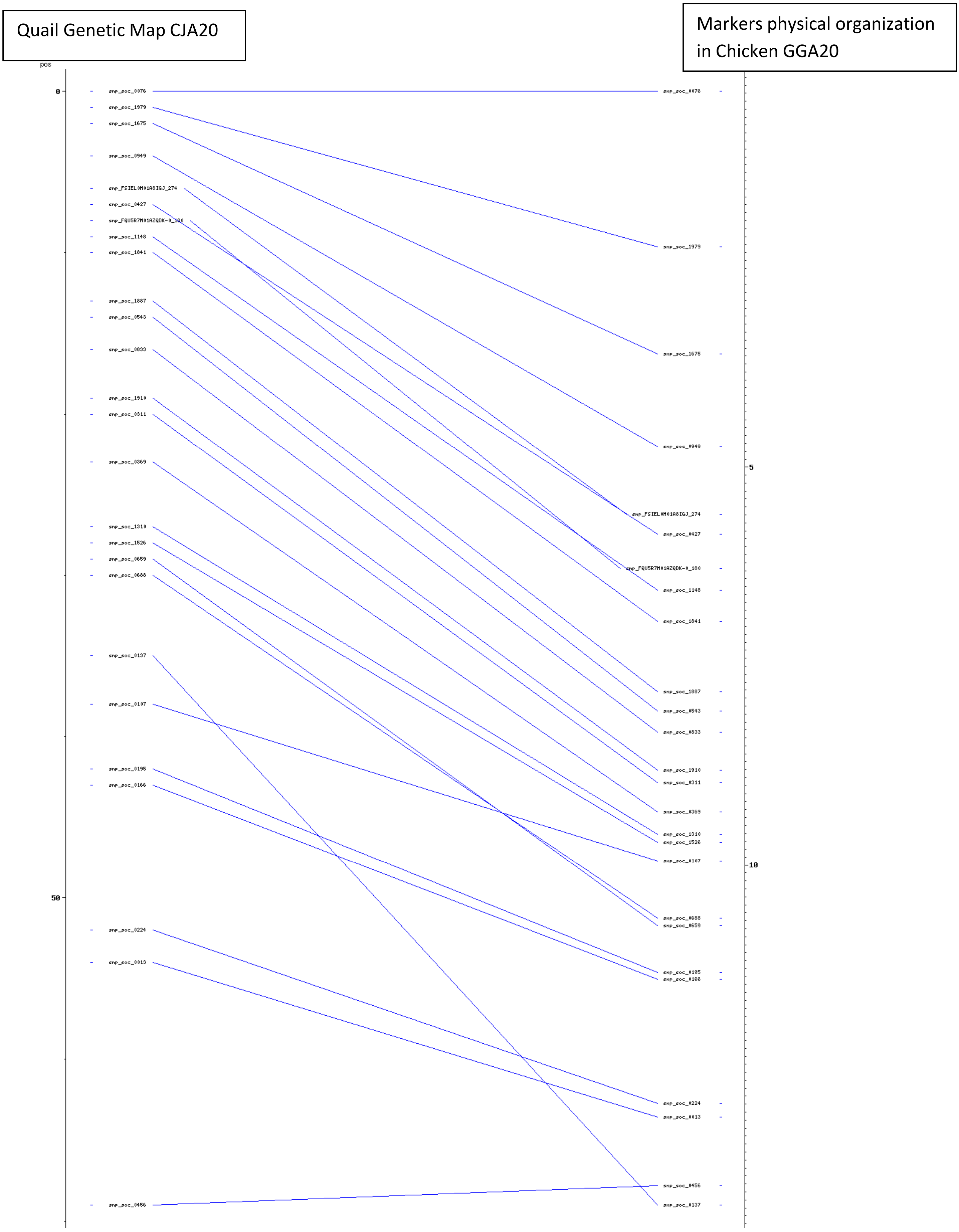

Chromosome 22

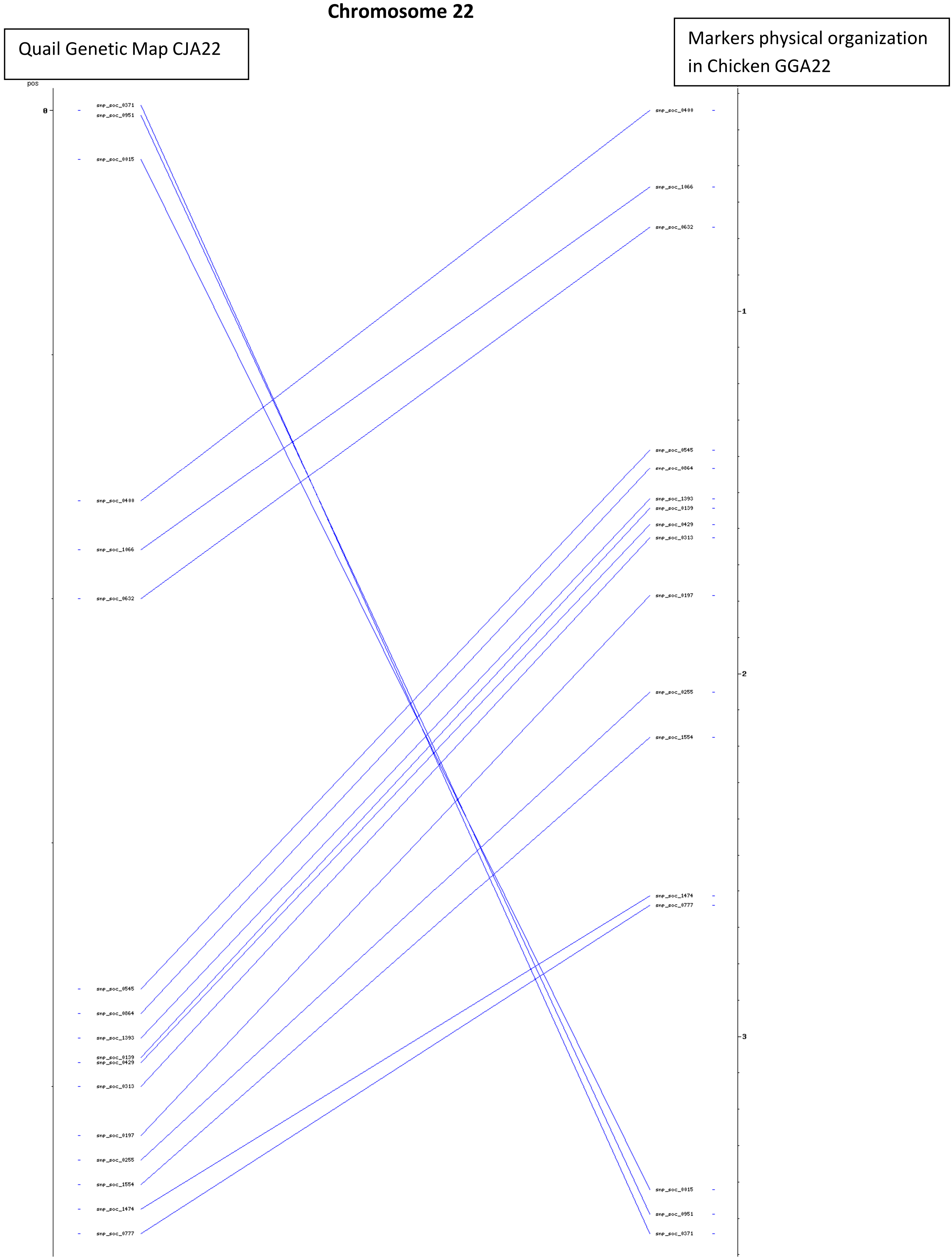

Chromosome 25

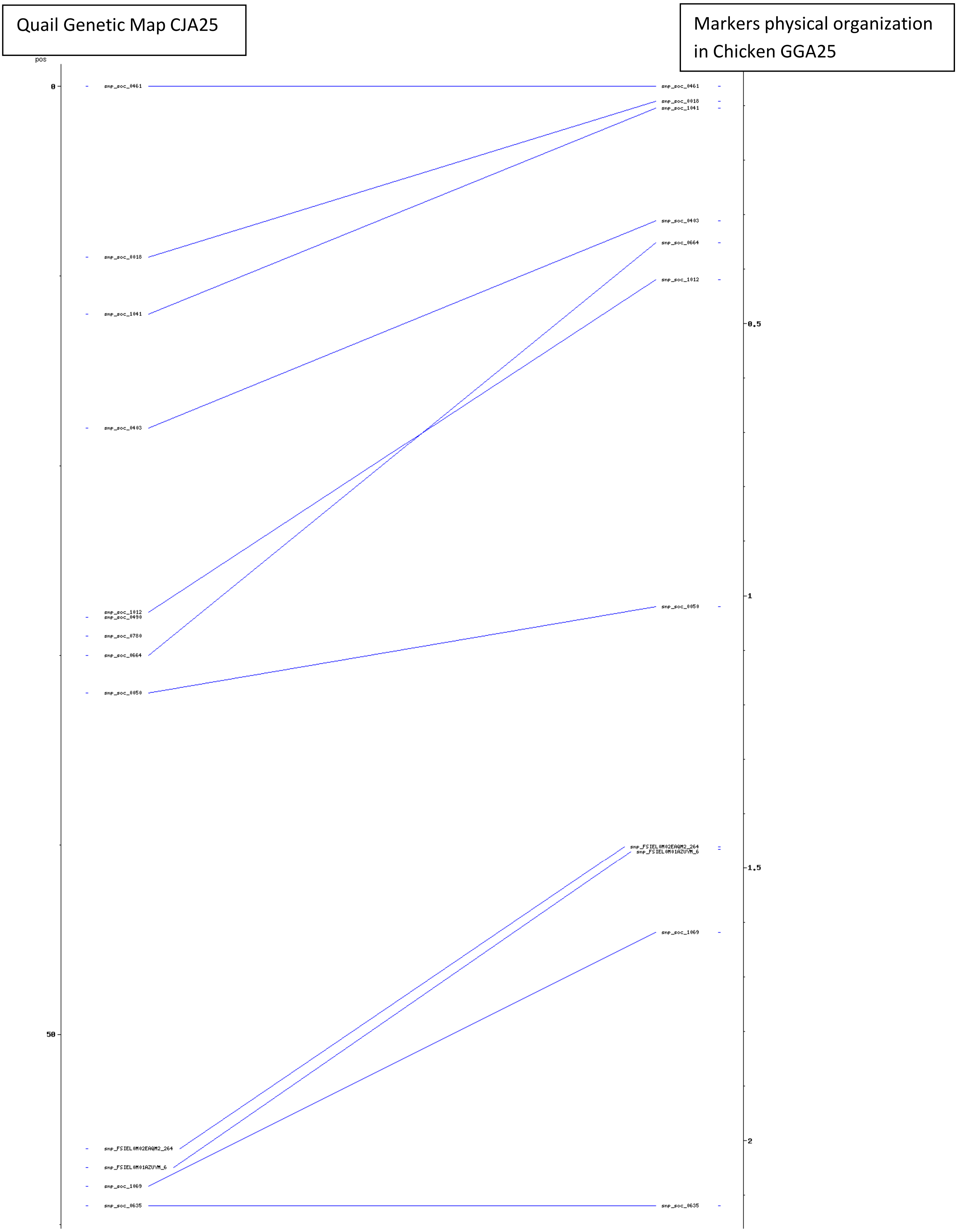

Additional Figure 3 ï Comparison between the Quail genetic map and the Chicken physical map.
Comparative cartography between the Quail genetic map (Left, in cM) and the Chicken physical organization of the markers (Right, in Mb). Crossing lines shows the existence of chromosomal rearrangements.
