# Supplementary material for: A medium density genetic map and QTL for behavioral and production traits in Japanese quail
Source: BMC Genomics. 2015 Jan 22;16(1):10. doi: 10.1186/s12864-014-1210-9 (PMC4307178; doi:10.1186/s12864-014-1210-9)
Supplement: Additional file 6: Figure S4. — Quail genetic map with positioned QTL. Graphic representation of the quail genetic map constructed with SNP markers. Longer chromosomes are shown in different parts; number is given in brackets. QTL identified were positioned on the map in colored boxes, with pink for social motivation-related QTL, red for emotional reactivity QTL, blue for aggressiveness QTL, and green for production QTL. Genome-wide significant QTL are represented in filled boxes and chromosome-wide significant QTL in shaded boxes. [file 12864_2014_1210_MOESM6_ESM.pdf]

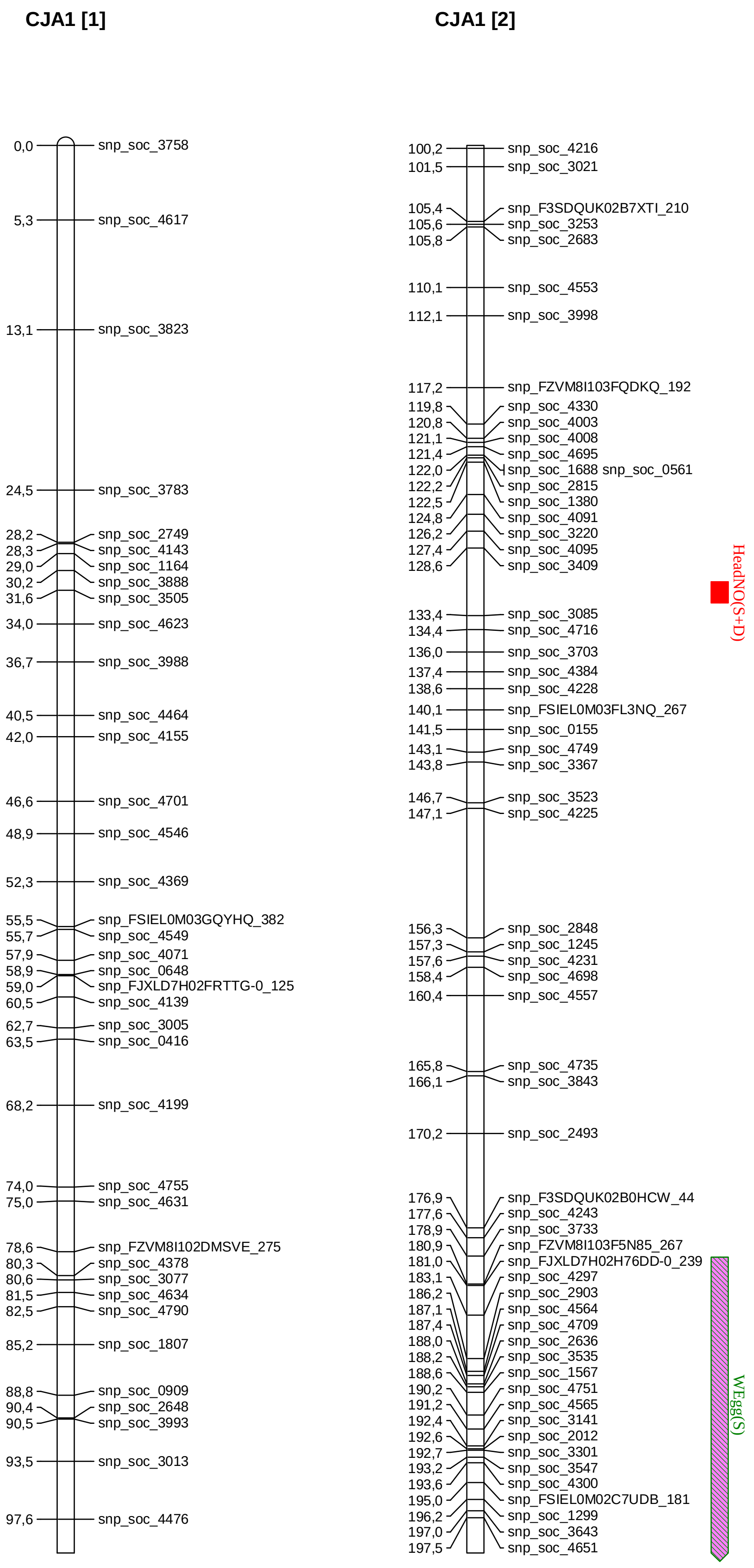
CJA1 [1]
0,0 snp_soc_3758
5,3 snp_soc_4617
13,1 snp_soc_3823
24,5 snp_soc_3783
28,2 snp_soc_2749
28,3 snp_soc_4143
29,0 snp_soc_1164
30,2 snp_soc_3888
31,6 snp_soc_3505
34,0 snp_soc_4623
36,7 snp_soc_3988
40,5 snp_soc_4464
42,0 snp_soc_4155
46,6 snp_soc_4701
48,9 snp_soc_4546
52,3 snp_soc_4369
55,5 snp_FSIEL0M03GQYHQ_382
55,7 snp_soc_4549
57,9 snp_soc_4071
58,9 snp_soc_0648
59,0 snp_FJXLD7H02FRTTG-0_125
60,5 snp_soc_4139
62,7 snp_soc_3005
63,5 snp_soc_0416
68,2 snp_soc_4199
74,0 snp_soc_4755
75,0 snp_soc_4631
78,6 snp_FZVM8I102DMSVE_275
80,3 snp_soc_4378
80,6 snp_soc_3077
81,5 snp_soc_4634
82,5 snp_soc_4790
85,2 snp_soc_1807
88,8 snp_soc_0909
90,4 snp_soc_2648
90,5 snp_soc_3993
93,5 snp_soc_3013
97,6 snp_soc_4476
CJA1 [2]
100,2 snp_soc_4216
101,5 snp_soc_3021
105,4 snp_F3SDQUK02B7XTI_210
105,6 snp_soc_3253
105,8 snp_soc_2683
110,1 snp_soc_4553
112,1 snp_soc_3998
117,2 snp_FZVM8I103FQDKQ_192
119,8 snp_soc_4330
120,8 snp_soc_4003
121,1 snp_soc_4008
121,4 snp_soc_4695
122,0 snp_soc_1688 snp_soc_0561
122,2 snp_soc_2815
122,5 snp_soc_1380
124,8 snp_soc_4091
126,2 snp_soc_3220
127,4 snp_soc_4095
128,6 snp_soc_3409
133,4 snp_soc_3085
134,4 snp_soc_4716
136,0 snp_soc_3703
137,4 snp_soc_4384
138,6 snp_soc_4228
140,1 snp_FSIEL0M03FL3NQ_267
141,5 snp_soc_0155
143,1 snp_soc_4749
143,8 snp_soc_3367
146,7 snp_soc_3523
147,1 snp_soc_4225
156,3 snp_soc_2848
157,3 snp_soc_1245
157,6 snp_soc_4231
158,4 snp_soc_4698
160,4 snp_soc_4557
165,8 snp_soc_4735
166,1 snp_soc_3843
170,2 snp_soc_2493
176,9 snp_F3SDQUK02B0HCW_44
177,6 snp_soc_4243
178,9 snp_soc_3733
180,9 snp_FZVM8I103F5N85_267
181,0 snp_FJXLD7H02H76DD-0_239
183,1 snp_soc_4297
186,2 snp_soc_2903
187,1 snp_soc_4564
187,4 snp_soc_4709
188,0 snp_soc_2636
188,2 snp_soc_3535
188,6 snp_soc_1567
190,2 snp_soc_4751
191,2 snp_soc_4565
192,4 snp_soc_3141
192,6 snp_soc_2012
192,7 snp_soc_3301
193,2 snp_soc_3547
193,6 snp_soc_4300
195,0 snp_FSIEL0M02C7UDB_181
196,2 snp_soc_1299
197,0 snp_soc_3643
197,5 snp_soc_4651
HeadNO(S+D)
WEgg(S)

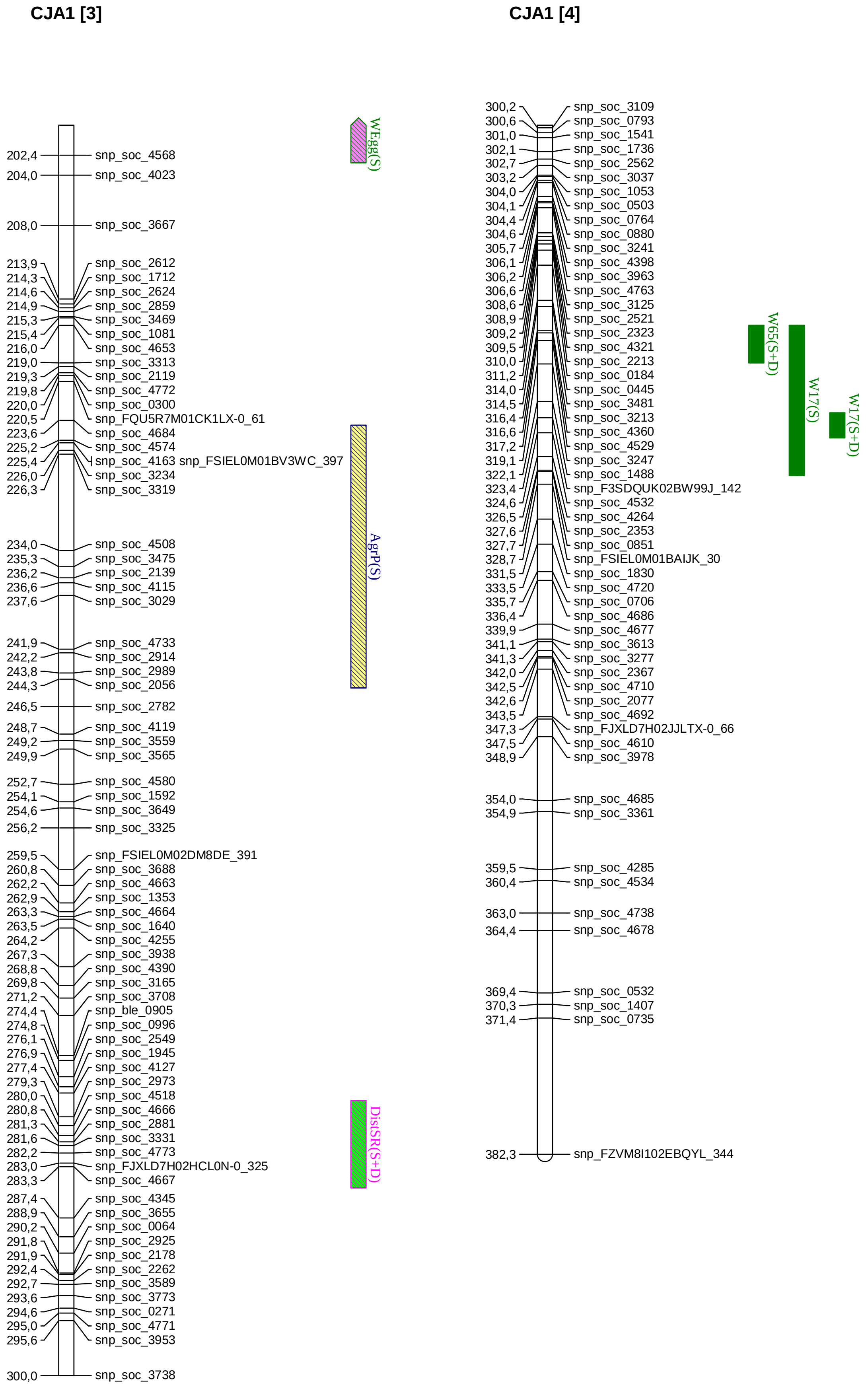
CJA1 [3]
202,4 snp_soc_4568
204,0 snp_soc_4023
208,0 snp_soc_3667
213,9 snp_soc_2612
214,3 snp_soc_1712
214,6 snp_soc_2624
214,9 snp_soc_2859
215,3 snp_soc_3469
215,4 snp_soc_1081
216,0 snp_soc_4653
219,0 snp_soc_3313
219,3 snp_soc_2119
219,8 snp_soc_4772
220,0 snp_soc_0300
220,5 snp_FQU5R7M01CK1LX-0_61
223,6 snp_soc_4684
225,2 snp_soc_4574
225,4 snp_soc_4163 snp_FSIEL0M01BV3WC_397
226,0 snp_soc_3234
226,3 snp_soc_3319
234,0 snp_soc_4508
235,3 snp_soc_3475
236,2 snp_soc_2139
236,6 snp_soc_4115
237,6 snp_soc_3029
241,9 snp_soc_4733
242,2 snp_soc_2914
243,8 snp_soc_2989
244,3 snp_soc_2056
246,5 snp_soc_2782
248,7 snp_soc_4119
249,2 snp_soc_3559
249,9 snp_soc_3565
252,7 snp_soc_4580
254,1 snp_soc_1592
254,6 snp_soc_3649
256,2 snp_soc_3325
259,5 snp_FSIEL0M02DM8DE_391
260,8 snp_soc_3688
262,2 snp_soc_4663
262,9 snp_soc_1353
263,3 snp_soc_4664
263,5 snp_soc_1640
264,2 snp_soc_4255
267,3 snp_soc_3938
268,8 snp_soc_4390
269,8 snp_soc_3165
271,2 snp_soc_3708
274,4 snp_ble_0905
274,8 snp_soc_0996
276,1 snp_soc_2549
276,9 snp_soc_1945
277,4 snp_soc_4127
279,3 snp_soc_2973
280,0 snp_soc_4518
280,8 snp_soc_4666
281,3 snp_soc_2881
281,6 snp_soc_3331
282,2 snp_soc_4773
283,0 snp_FJXLD7H02HCL0N-0_325
283,3 snp_soc_4667
287,4 snp_soc_4345
288,9 snp_soc_3655
290,2 snp_soc_0064
291,8 snp_soc_2925
291,9 snp_soc_2178
292,4 snp_soc_2262
292,7 snp_soc_3589
293,6 snp_soc_3773
294,6 snp_soc_0271
295,0 snp_soc_4771
295,6 snp_soc_3953
300,0 snp_soc_3738
WEgg(S)
AgrP(S)
DistSR(S+D)
CJA1 [4]
300,2 snp_soc_3109
300,6 snp_soc_0793
301,0 snp_soc_1541
302,1 snp_soc_1736
302,7 snp_soc_2562
303,2 snp_soc_3037
304,0 snp_soc_1053
304,1 snp_soc_0503
304,4 snp_soc_0764
304,6 snp_soc_0880
305,7 snp_soc_3241
306,1 snp_soc_4398
306,2 snp_soc_3963
306,6 snp_soc_4763
308,6 snp_soc_3125
308,9 snp_soc_2521
309,2 snp_soc_2323
309,5 snp_soc_4321
310,0 snp_soc_2213
311,2 snp_soc_0184
314,0 snp_soc_0445
314,5 snp_soc_3481
316,4 snp_soc_3213
316,6 snp_soc_4360
317,2 snp_soc_4529
319,1 snp_soc_3247
322,1 snp_soc_1488
323,4 snp_F3SDQUK02BW99J_142
324,6 snp_soc_4532
326,5 snp_soc_4264
327,6 snp_soc_2353
327,7 snp_soc_0851
328,7 snp_FSIEL0M01BAIJK_30
331,5 snp_soc_1830
333,5 snp_soc_4720
335,7 snp_soc_0706
336,4 snp_soc_4686
339,9 snp_soc_4677
341,1 snp_soc_3613
341,3 snp_soc_3277
342,0 snp_soc_2367
342,5 snp_soc_4710
342,6 snp_soc_2077
343,5 snp_soc_4692
347,3 snp_FJXLD7H02JJLTX-0_66
347,5 snp_soc_4610
348,9 snp_soc_3978
354,0 snp_soc_4685
354,9 snp_soc_3361
359,5 snp_soc_4285
360,4 snp_soc_4534
363,0 snp_soc_4738
364,4 snp_soc_4678
369,4 snp_soc_0532
370,3 snp_soc_1407
371,4 snp_soc_0735
382,3 snp_FZVM8I102EBQYL_344
W65(S+D)
W17(S)
W17(S+D)

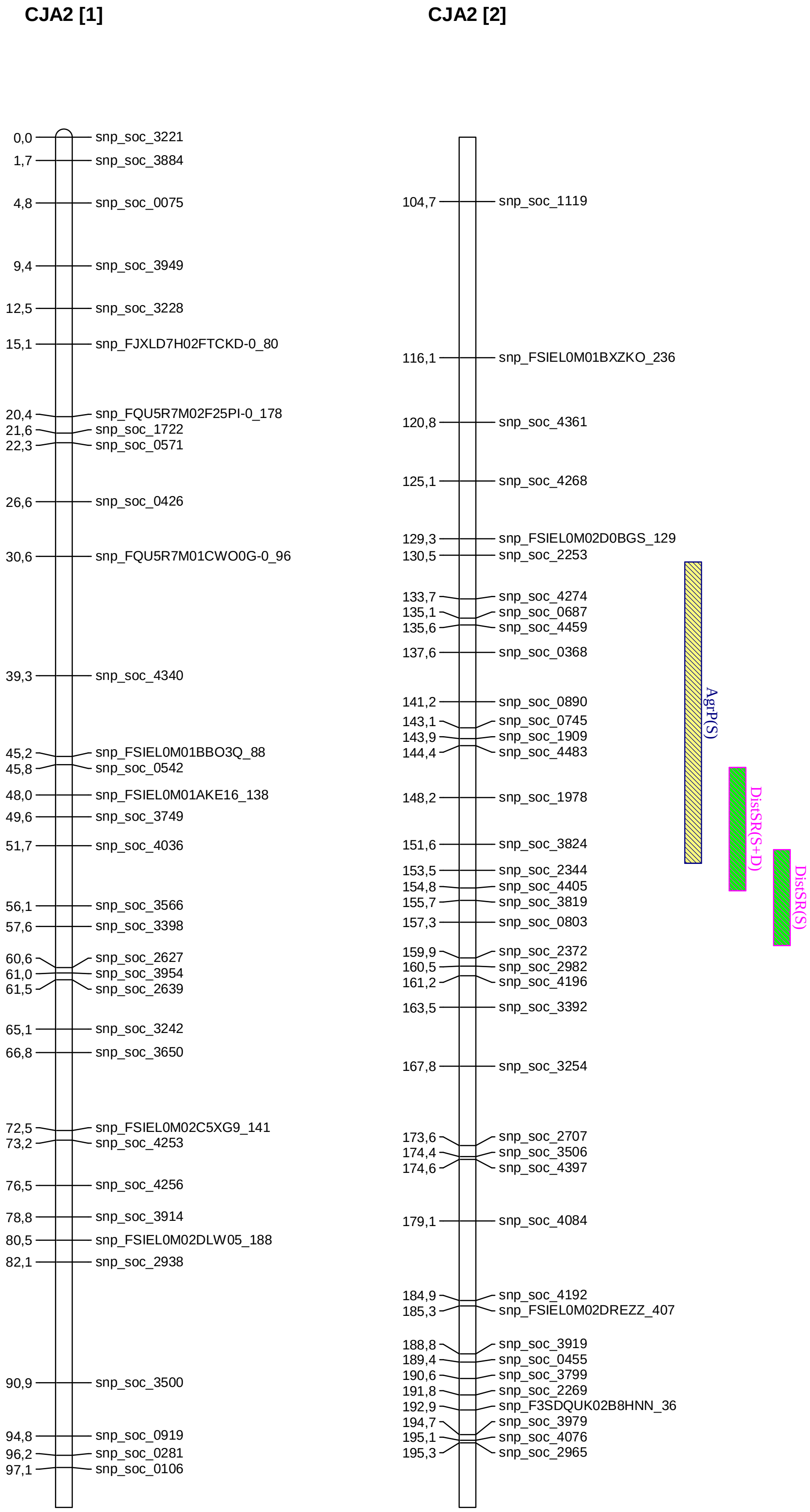
CJA2 [1]
0,0 snp_soc_3221
1,7 snp_soc_3884
4,8 snp_soc_0075
9,4 snp_soc_3949
12,5 snp_soc_3228
15,1 snp_FJXLD7H02FTCKD-0_80
20,4 snp_FQU5R7M02F25PI-0_178
21,6 snp_soc_1722
22,3 snp_soc_0571
26,6 snp_soc_0426
30,6 snp_FQU5R7M01CWO0G-0_96
39,3 snp_soc_4340
45,2 snp_FSIEL0M01BBO3Q_88
45,8 snp_soc_0542
48,0 snp_FSIEL0M01AKE16_138
49,6 snp_soc_3749
51,7 snp_soc_4036
56,1 snp_soc_3566
57,6 snp_soc_3398
60,6 snp_soc_2627
61,0 snp_soc_3954
61,5 snp_soc_2639
65,1 snp_soc_3242
66,8 snp_soc_3650
72,5 snp_FSIEL0M02C5XG9_141
73,2 snp_soc_4253
76,5 snp_soc_4256
78,8 snp_soc_3914
80,5 snp_FSIEL0M02DLW05_188
82,1 snp_soc_2938
90,9 snp_soc_3500
94,8 snp_soc_0919
96,2 snp_soc_0281
97,1 snp_soc_0106
CJA2 [2]
104,7 snp_soc_1119
116,1 snp_FSIEL0M01BXZKO_236
120,8 snp_soc_4361
125,1 snp_soc_4268
129,3 snp_FSIEL0M02D0BGS_129
130,5 snp_soc_2253
133,7 snp_soc_4274
135,1 snp_soc_0687
135,6 snp_soc_4459
137,6 snp_soc_0368
141,2 snp_soc_0890
143,1 snp_soc_0745
143,9 snp_soc_1909
144,4 snp_soc_4483
148,2 snp_soc_1978
151,6 snp_soc_3824
153,5 snp_soc_2344
154,8 snp_soc_4405
155,7 snp_soc_3819
157,3 snp_soc_0803
159,9 snp_soc_2372
160,5 snp_soc_2982
161,2 snp_soc_4196
163,5 snp_soc_3392
167,8 snp_soc_3254
173,6 snp_soc_2707
174,4 snp_soc_3506
174,6 snp_soc_4397
179,1 snp_soc_4084
184,9 snp_soc_4192
185,3 snp_FSIEL0M02DREZZ_407
188,8 snp_soc_3919
189,4 snp_soc_0455
190,6 snp_soc_3799
191,8 snp_soc_2269
192,9 snp_F3SDQUK02B8HNN_36
194,7 snp_soc_3979
195,1 snp_soc_4076
195,3 snp_soc_2965
AgrP(S)
DistSR(S+D)
DistSR(S)

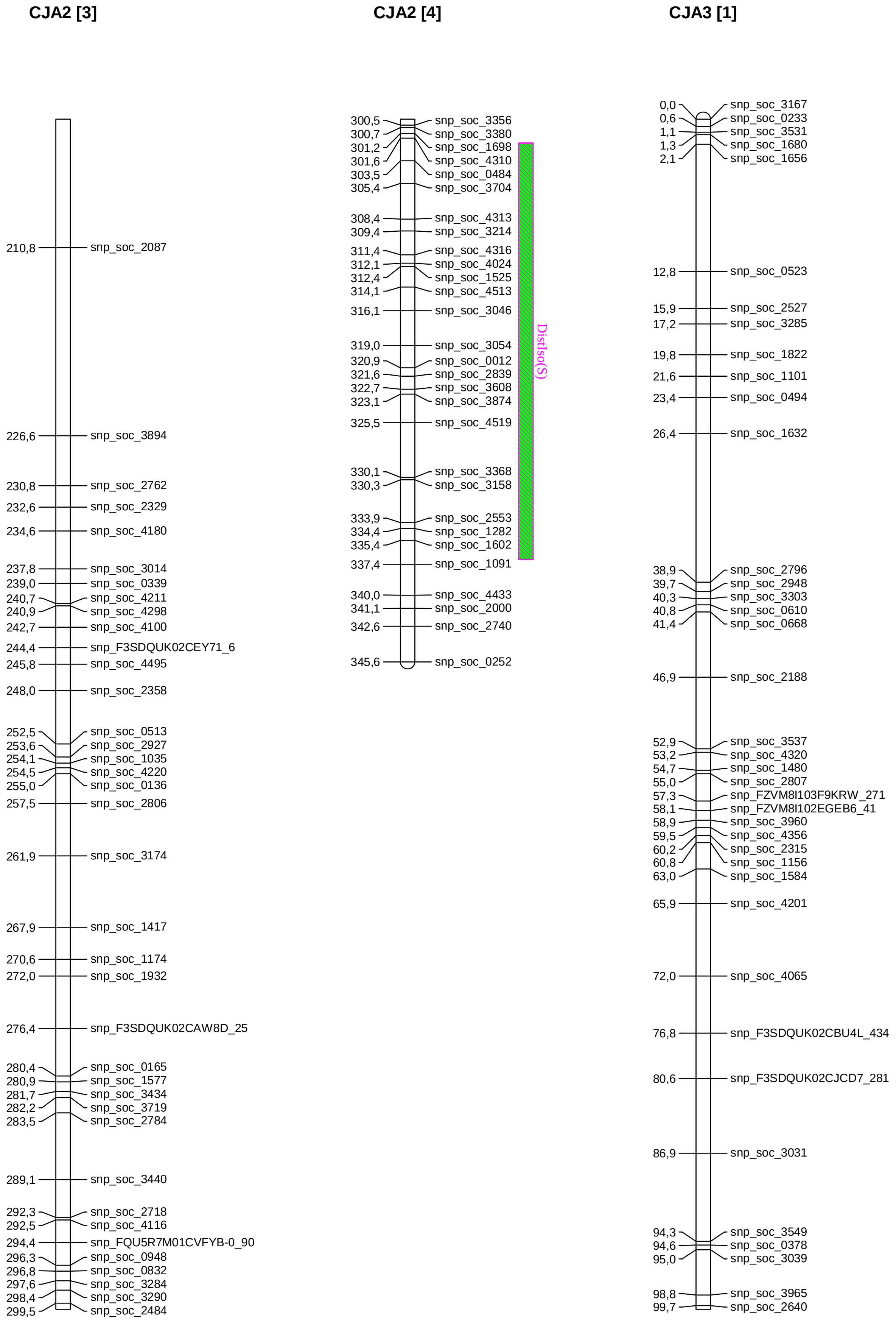
CJA2 [3]
210,8 snp_soc_2087
226,6 snp_soc_3894
230,8 snp_soc_2762
232,6 snp_soc_2329
234,6 snp_soc_4180
237,8 snp_soc_3014
239,0 snp_soc_0339
240,7 snp_soc_4211
240,9 snp_soc_4298
242,7 snp_soc_4100
244,4 snp_F3SDQUK02CEY71_6
245,8 snp_soc_4495
248,0 snp_soc_2358
252,5 snp_soc_0513
253,6 snp_soc_2927
254,1 snp_soc_1035
254,5 snp_soc_4220
255,0 snp_soc_0136
257,5 snp_soc_2806
261,9 snp_soc_3174
267,9 snp_soc_1417
270,6 snp_soc_1174
272,0 snp_soc_1932
276,4 snp_F3SDQUK02CAW8D_25
280,4 snp_soc_0165
280,9 snp_soc_1577
281,7 snp_soc_3434
282,2 snp_soc_3719
283,5 snp_soc_2784
289,1 snp_soc_3440
292,3 snp_soc_2718
292,5 snp_soc_4116
294,4 snp_FQU5R7M01CVFYB-0_90
296,3 snp_soc_0948
296,8 snp_soc_0832
297,6 snp_soc_3284
298,4 snp_soc_3290
299,5 snp_soc_2484
CJA2 [4]
300,5 snp_soc_3356
300,7 snp_soc_3380
301,2 snp_soc_1698
301,6 snp_soc_4310
303,5 snp_soc_0484
305,4 snp_soc_3704
308,4 snp_soc_4313
309,4 snp_soc_3214
311,4 snp_soc_4316
312,1 snp_soc_4024
312,4 snp_soc_1525
314,1 snp_soc_4513
316,1 snp_soc_3046
319,0 snp_soc_3054
320,9 snp_soc_0012
321,6 snp_soc_2839
322,7 snp_soc_3608
323,1 snp_soc_3874
325,5 snp_soc_4519
330,1 snp_soc_3368
330,3 snp_soc_3158
333,9 snp_soc_2553
334,4 snp_soc_1282
335,4 snp_soc_1602
337,4 snp_soc_1091
340,0 snp_soc_4433
341,1 snp_soc_2000
342,6 snp_soc_2740
345,6 snp_soc_0252
DistIso(S)
CJA3 [1]
0,0 snp_soc_3167
0,6 snp_soc_0233
1,1 snp_soc_3531
1,3 snp_soc_1680
2,1 snp_soc_1656
12,8 snp_soc_0523
15,9 snp_soc_2527
17,2 snp_soc_3285
19,8 snp_soc_1822
21,6 snp_soc_1101
23,4 snp_soc_0494
26,4 snp_soc_1632
38,9 snp_soc_2796
39,7 snp_soc_2948
40,3 snp_soc_3303
40,8 snp_soc_0610
41,4 snp_soc_0668
46,9 snp_soc_2188
52,9 snp_soc_3537
53,2 snp_soc_4320
54,7 snp_soc_1480
55,0 snp_soc_2807
57,3 snp_FZVM8I103F9KRW_271
58,1 snp_FZVM8I102EGEB6_41
58,9 snp_soc_3960
59,5 snp_soc_4356
60,2 snp_soc_2315
60,8 snp_soc_1156
63,0 snp_soc_1584
65,9 snp_soc_4201
72,0 snp_soc_4065
76,8 snp_F3SDQUK02CBU4L_434
80,6 snp_F3SDQUK02CJCD7_281
86,9 snp_soc_3031
94,3 snp_soc_3549
94,6 snp_soc_0378
95,0 snp_soc_3039
98,8 snp_soc_3965
99,7 snp_soc_2640

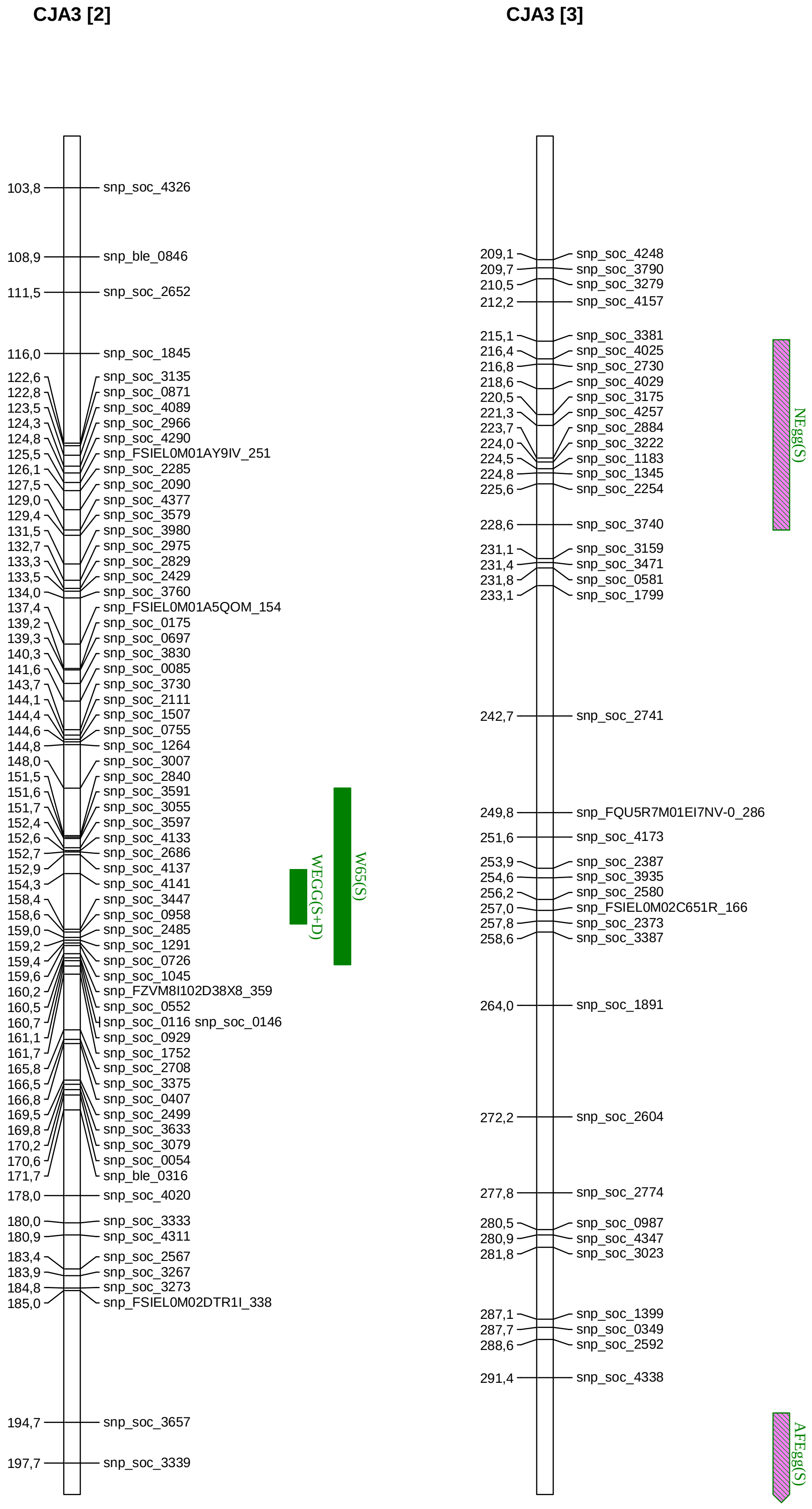
CJA3 [2]
103,8 snp_soc_4326
108,9 snp_ble_0846
111,5 snp_soc_2652
116,0 snp_soc_1845
122,6 snp_soc_3135
122,8 snp_soc_0871
123,5 snp_soc_4089
124,3 snp_soc_2966
124,8 snp_soc_4290
125,5 snp_FSIEL0M01AY9IV_251
126,1 snp_soc_2285
127,5 snp_soc_2090
129,0 snp_soc_4377
129,4 snp_soc_3579
131,5 snp_soc_3980
132,7 snp_soc_2975
133,3 snp_soc_2829
133,5 snp_soc_2429
134,0 snp_soc_3760
137,4 snp_FSIEL0M01A5QOM_154
139,2 snp_soc_0175
139,3 snp_soc_0697
140,3 snp_soc_3830
141,6 snp_soc_0085
143,7 snp_soc_3730
144,1 snp_soc_2111
144,4 snp_soc_1507
144,6 snp_soc_0755
144,8 snp_soc_1264
148,0 snp_soc_3007
151,5 snp_soc_2840
151,6 snp_soc_3591
151,7 snp_soc_3055
152,4 snp_soc_3597
152,6 snp_soc_4133
152,7 snp_soc_2686
152,9 snp_soc_4137
154,3 snp_soc_4141
158,4 snp_soc_3447
158,6 snp_soc_0958
159,0 snp_soc_2485
159,2 snp_soc_1291
159,4 snp_soc_0726
159,6 snp_soc_1045
160,2 snp_FZVM8I102D38X8_359
160,5 snp_soc_0552
160,7 snp_soc_0116 snp_soc_0146
161,1 snp_soc_0929
161,7 snp_soc_1752
165,8 snp_soc_2708
166,5 snp_soc_3375
166,8 snp_soc_0407
169,5 snp_soc_2499
169,8 snp_soc_3633
170,2 snp_soc_3079
170,6 snp_soc_0054
171,7 snp_ble_0316
178,0 snp_soc_4020
180,0 snp_soc_3333
180,9 snp_soc_4311
183,4 snp_soc_2567
183,9 snp_soc_3267
184,8 snp_soc_3273
185,0 snp_FSIEL0M02DTR1I_338
194,7 snp_soc_3657
197,7 snp_soc_3339
W65(S)
WEGG(S+D)
CJA3 [3]
209,1 snp_soc_4248
209,7 snp_soc_3790
210,5 snp_soc_3279
212,2 snp_soc_4157
215,1 snp_soc_3381
216,4 snp_soc_4025
216,8 snp_soc_2730
218,6 snp_soc_4029
220,5 snp_soc_3175
221,3 snp_soc_4257
223,7 snp_soc_2884
224,0 snp_soc_3222
224,5 snp_soc_1183
224,8 snp_soc_1345
225,6 snp_soc_2254
228,6 snp_soc_3740
231,1 snp_soc_3159
231,4 snp_soc_3471
231,8 snp_soc_0581
233,1 snp_soc_1799
242,7 snp_soc_2741
249,8 snp_FQU5R7M01EI7NV-0_286
251,6 snp_soc_4173
253,9 snp_soc_2387
254,6 snp_soc_3935
256,2 snp_soc_2580
257,0 snp_FSIEL0M02C651R_166
257,8 snp_soc_2373
258,6 snp_soc_3387
264,0 snp_soc_1891
272,2 snp_soc_2604
277,8 snp_soc_2774
280,5 snp_soc_0987
280,9 snp_soc_4347
281,8 snp_soc_3023
287,1 snp_soc_1399
287,7 snp_soc_0349
288,6 snp_soc_2592
291,4 snp_soc_4338
NEgg(S)
AFEgg(S)

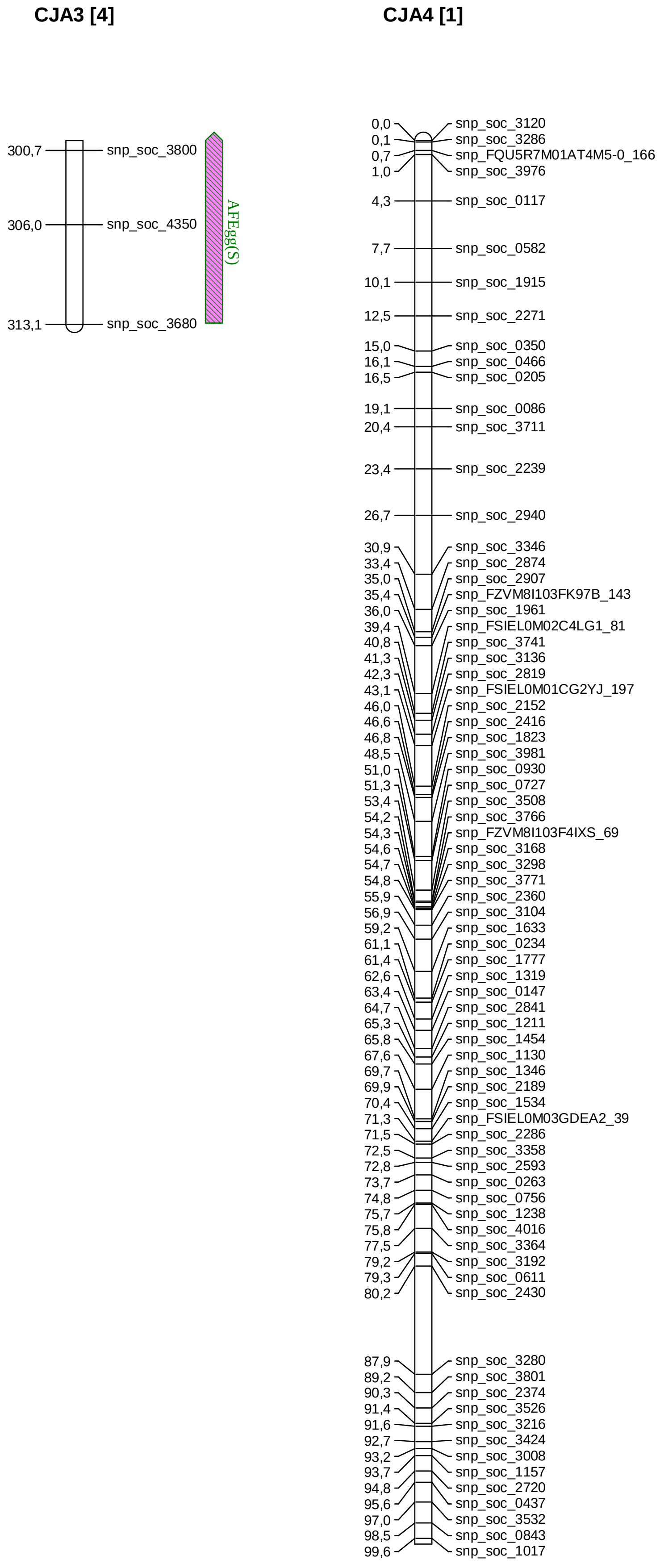

CJA3 [4]
300,7 snp_soc_3800
306,0 snp_soc_4350
313,1 snp_soc_3680
AFEgg(S)
CJA4 [1]
0,0 snp_soc_3120
0,1 snp_soc_3286
0,7 snp_FQU5R7M01AT4M5-0_166
1,0 snp_soc_3976
4,3 snp_soc_0117
7,7 snp_soc_0582
10,1 snp_soc_1915
12,5 snp_soc_2271
15,0 snp_soc_0350
16,1 snp_soc_0466
16,5 snp_soc_0205
19,1 snp_soc_0086
20,4 snp_soc_3711
23,4 snp_soc_2239
26,7 snp_soc_2940
30,9 snp_soc_3346
33,4 snp_soc_2874
35,0 snp_soc_2907
35,4 snp_FZVM8I103FK97B_143
36,0 snp_soc_1961
39,4 snp_FSIEL0M02C4LG1_81
40,8 snp_soc_3741
41,3 snp_soc_3136
42,3 snp_soc_2819
43,1 snp_FSIEL0M01CG2YJ_197
46,0 snp_soc_2152
46,6 snp_soc_2416
46,8 snp_soc_1823
48,5 snp_soc_3981
51,0 snp_soc_0930
51,3 snp_soc_0727
53,4 snp_soc_3508
54,2 snp_soc_3766
54,3 snp_FZVM8I103F4IXS_69
54,6 snp_soc_3168
54,7 snp_soc_3298
54,8 snp_soc_3771
55,9 snp_soc_2360
56,9 snp_soc_3104
59,2 snp_soc_1633
61,1 snp_soc_0234
61,4 snp_soc_1777
62,6 snp_soc_1319
63,4 snp_soc_0147
64,7 snp_soc_2841
65,3 snp_soc_1211
65,8 snp_soc_1454
67,6 snp_soc_1130
69,7 snp_soc_1346
69,9 snp_soc_2189
70,4 snp_soc_1534
71,3 snp_FSIEL0M03GDEA2_39
71,5 snp_soc_2286
72,5 snp_soc_3358
72,8 snp_soc_2593
73,7 snp_soc_0263
74,8 snp_soc_0756
75,7 snp_soc_1238
75,8 snp_soc_4016
77,5 snp_soc_3364
79,2 snp_soc_3192
79,3 snp_soc_0611
80,2 snp_soc_2430
87,9 snp_soc_3280
89,2 snp_soc_3801
90,3 snp_soc_2374
91,4 snp_soc_3526
91,6 snp_soc_3216
92,7 snp_soc_3424
93,2 snp_soc_3008
93,7 snp_soc_1157
94,8 snp_soc_2720
95,6 snp_soc_0437
97,0 snp_soc_3532
98,5 snp_soc_0843
99,6 snp_soc_1017

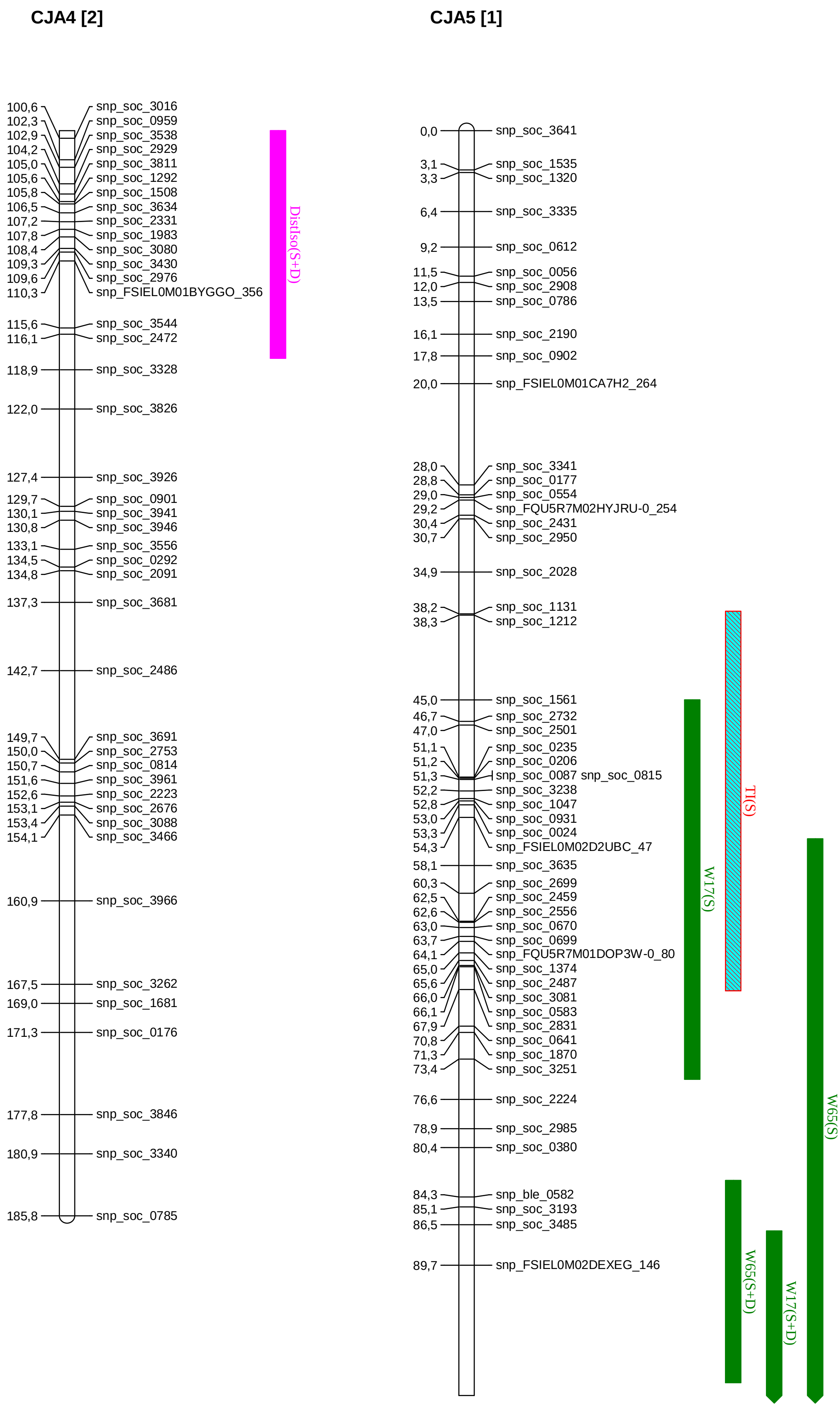

CJA4 [2]
CJA5 [1]
100,6 snp_soc_3016
102,3 snp_soc_0959
102,9 snp_soc_3538
104,2 snp_soc_2929
105,0 snp_soc_3811
105,6 snp_soc_1292
105,8 snp_soc_1508
106,5 snp_soc_3634
107,2 snp_soc_2331
107,8 snp_soc_1983
108,4 snp_soc_3080
109,3 snp_soc_3430
109,6 snp_soc_2976
110,3 snp_FSIEL0M01BYGGO_356
115,6 snp_soc_3544
116,1 snp_soc_2472
118,9 snp_soc_3328
122,0 snp_soc_3826
127,4 snp_soc_3926
129,7 snp_soc_0901
130,1 snp_soc_3941
130,8 snp_soc_3946
133,1 snp_soc_3556
134,5 snp_soc_0292
134,8 snp_soc_2091
137,3 snp_soc_3681
142,7 snp_soc_2486
149,7 snp_soc_3691
150,0 snp_soc_2753
150,7 snp_soc_0814
151,6 snp_soc_3961
152,6 snp_soc_2223
153,1 snp_soc_2676
153,4 snp_soc_3088
154,1 snp_soc_3466
160,9 snp_soc_3966
167,5 snp_soc_3262
169,0 snp_soc_1681
171,3 snp_soc_0176
177,8 snp_soc_3846
180,9 snp_soc_3340
185,8 snp_soc_0785
DistIso(S+D)
0,0 snp_soc_3641
3,1 snp_soc_1535
3,3 snp_soc_1320
6,4 snp_soc_3335
9,2 snp_soc_0612
11,5 snp_soc_0056
12,0 snp_soc_2908
13,5 snp_soc_0786
16,1 snp_soc_2190
17,8 snp_soc_0902
20,0 snp_FSIEL0M01CA7H2_264
28,0 snp_soc_3341
28,8 snp_soc_0177
29,0 snp_soc_0554
29,2 snp_FQU5R7M02HYJRU-0_254
30,4 snp_soc_2431
30,7 snp_soc_2950
34,9 snp_soc_2028
38,2 snp_soc_1131
38,3 snp_soc_1212
45,0 snp_soc_1561
46,7 snp_soc_2732
47,0 snp_soc_2501
51,1 snp_soc_0235
51,2 snp_soc_0206
51,3 snp_soc_0087 snp_soc_0815
52,2 snp_soc_3238
52,8 snp_soc_1047
53,0 snp_soc_0931
53,3 snp_soc_0024
54,3 snp_FSIEL0M02D2UBC_47
58,1 snp_soc_3635
60,3 snp_soc_2699
62,5 snp_soc_2459
62,6 snp_soc_2556
63,0 snp_soc_0670
63,7 snp_soc_0699
64,1 snp_FQU5R7M01DOP3W-0_80
65,0 snp_soc_1374
65,6 snp_soc_2487
66,0 snp_soc_3081
66,1 snp_soc_0583
67,9 snp_soc_2831
70,8 snp_soc_0641
71,3 snp_soc_1870
73,4 snp_soc_3251
76,6 snp_soc_2224
78,9 snp_soc_2985
80,4 snp_soc_0380
84,3 snp_ble_0582
85,1 snp_soc_3193
86,5 snp_soc_3485
89,7 snp_FSIEL0M02DEXEG_146
TI(S)
W17(S)
W65(S)
W65(S+D)
W17(S+D)

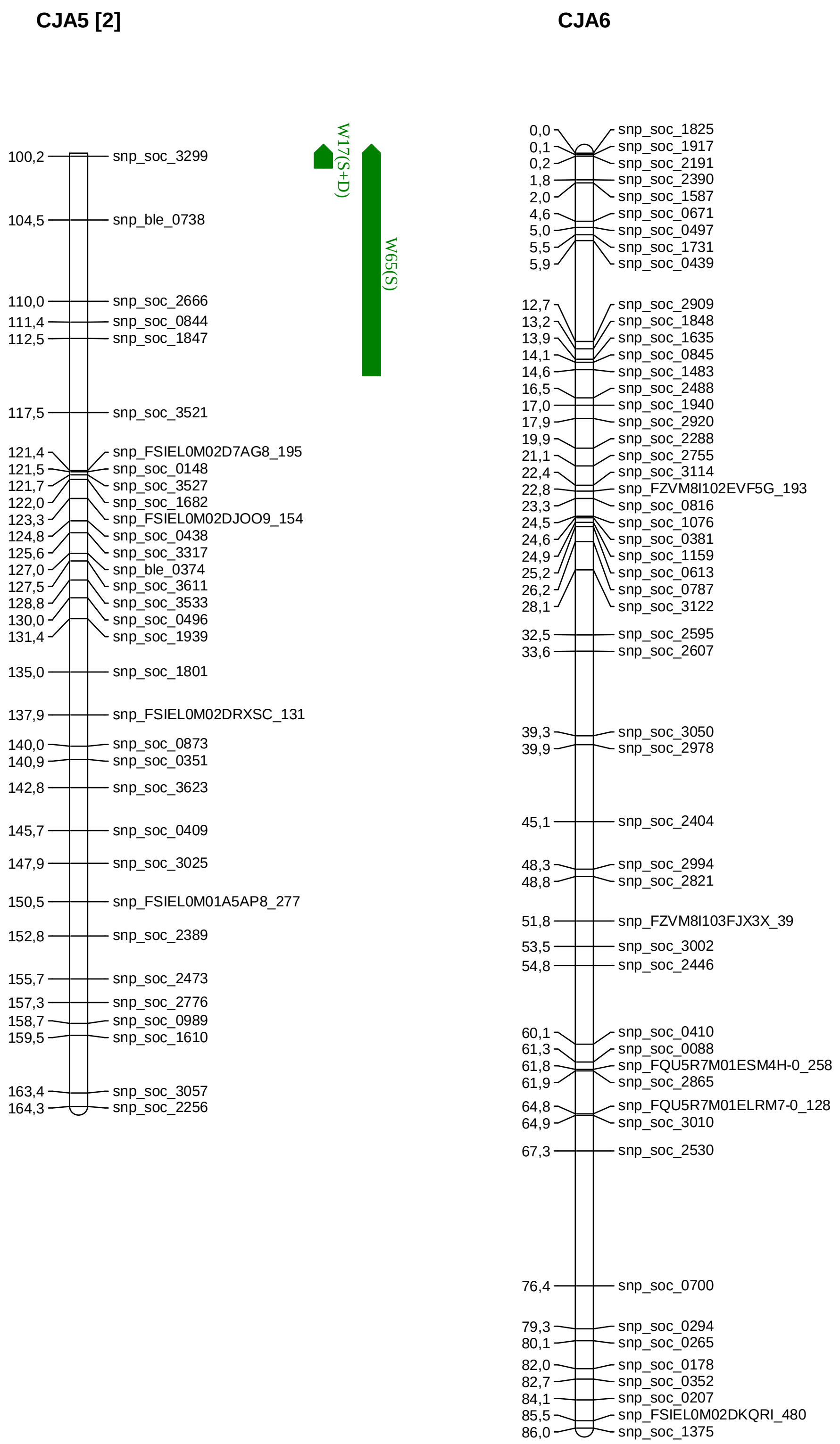
CJA5 [2]
CJA6
W17(S+D)
W65(S)
100,2 snp_soc_3299
104,5 snp_ble_0738
110,0 snp_soc_2666
111,4 snp_soc_0844
112,5 snp_soc_1847
117,5 snp_soc_3521
121,4 snp_FSIEL0M02D7AG8_195
121,5 snp_soc_0148
121,7 snp_soc_3527
122,0 snp_soc_1682
123,3 snp_FSIEL0M02DJOO9_154
124,8 snp_soc_0438
125,6 snp_soc_3317
127,0 snp_ble_0374
127,5 snp_soc_3611
128,8 snp_soc_3533
130,0 snp_soc_0496
131,4 snp_soc_1939
135,0 snp_soc_1801
137,9 snp_FSIEL0M02DRXSC_131
140,0 snp_soc_0873
140,9 snp_soc_0351
142,8 snp_soc_3623
145,7 snp_soc_0409
147,9 snp_soc_3025
150,5 snp_FSIEL0M01A5AP8_277
152,8 snp_soc_2389
155,7 snp_soc_2473
157,3 snp_soc_2776
158,7 snp_soc_0989
159,5 snp_soc_1610
163,4 snp_soc_3057
164,3 snp_soc_2256
0,0 snp_soc_1825
0,1 snp_soc_1917
0,2 snp_soc_2191
1,8 snp_soc_2390
2,0 snp_soc_1587
4,6 snp_soc_0671
5,0 snp_soc_0497
5,5 snp_soc_1731
5,9 snp_soc_0439
12,7 snp_soc_2909
13,2 snp_soc_1848
13,9 snp_soc_1635
14,1 snp_soc_0845
14,6 snp_soc_1483
16,5 snp_soc_2488
17,0 snp_soc_1940
17,9 snp_soc_2920
19,9 snp_soc_2288
21,1 snp_soc_2755
22,4 snp_soc_3114
22,8 snp_FZVM8I102EVF5G_193
23,3 snp_soc_0816
24,5 snp_soc_1076
24,6 snp_soc_0381
24,9 snp_soc_1159
25,2 snp_soc_0613
26,2 snp_soc_0787
28,1 snp_soc_3122
32,5 snp_soc_2595
33,6 snp_soc_2607
39,3 snp_soc_3050
39,9 snp_soc_2978
45,1 snp_soc_2404
48,3 snp_soc_2994
48,8 snp_soc_2821
51,8 snp_FZVM8I103FJX3X_39
53,5 snp_soc_3002
54,8 snp_soc_2446
60,1 snp_soc_0410
61,3 snp_soc_0088
61,8 snp_FQU5R7M01ESM4H-0_258
61,9 snp_soc_2865
64,8 snp_FQU5R7M01ELRM7-0_128
64,9 snp_soc_3010
67,3 snp_soc_2530
76,4 snp_soc_0700
79,3 snp_soc_0294
80,1 snp_soc_0265
82,0 snp_soc_0178
82,7 snp_soc_0352
84,1 snp_soc_0207
85,5 snp_FSIEL0M02DKQRI_480
86,0 snp_soc_1375

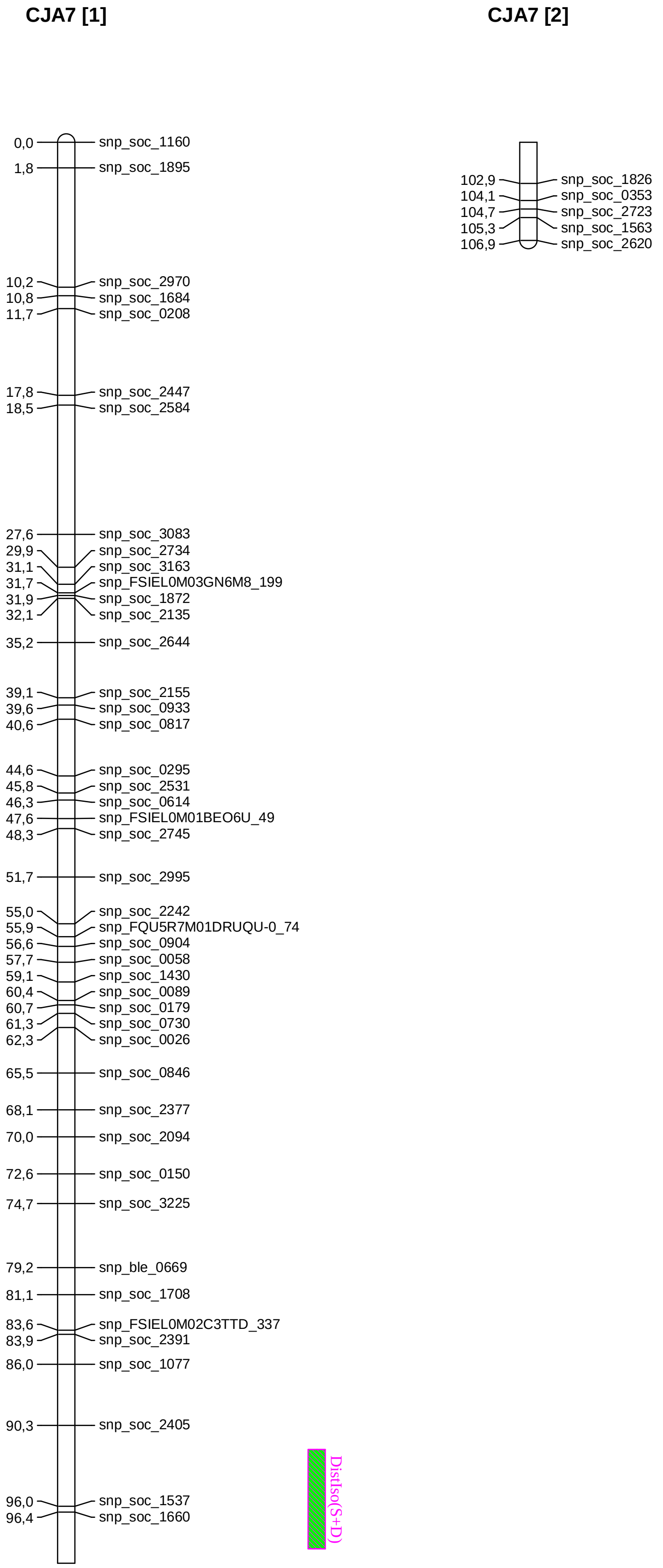
CJA7 [1]
0,0 snp_soc_1160
1,8 snp_soc_1895
10,2 snp_soc_2970
10,8 snp_soc_1684
11,7 snp_soc_0208
17,8 snp_soc_2447
18,5 snp_soc_2584
27,6 snp_soc_3083
29,9 snp_soc_2734
31,1 snp_soc_3163
31,7 snp_FSIEL0M03GN6M8_199
31,9 snp_soc_1872
32,1 snp_soc_2135
35,2 snp_soc_2644
39,1 snp_soc_2155
39,6 snp_soc_0933
40,6 snp_soc_0817
44,6 snp_soc_0295
45,8 snp_soc_2531
46,3 snp_soc_0614
47,6 snp_FSIEL0M01BEO6U_49
48,3 snp_soc_2745
51,7 snp_soc_2995
55,0 snp_soc_2242
55,9 snp_FQU5R7M01DRUQU-0_74
56,6 snp_soc_0904
57,7 snp_soc_0058
59,1 snp_soc_1430
60,4 snp_soc_0089
60,7 snp_soc_0179
61,3 snp_soc_0730
62,3 snp_soc_0026
65,5 snp_soc_0846
68,1 snp_soc_2377
70,0 snp_soc_2094
72,6 snp_soc_0150
74,7 snp_soc_3225
79,2 snp_ble_0669
81,1 snp_soc_1708
83,6 snp_FSIEL0M02C3TTD_337
83,9 snp_soc_2391
86,0 snp_soc_1077
90,3 snp_soc_2405
96,0 snp_soc_1537
96,4 snp_soc_1660
DistIso(S+D)
CJA7 [2]
102,9 snp_soc_1826
104,1 snp_soc_0353
104,7 snp_soc_2723
105,3 snp_soc_1563
106,9 snp_soc_2620

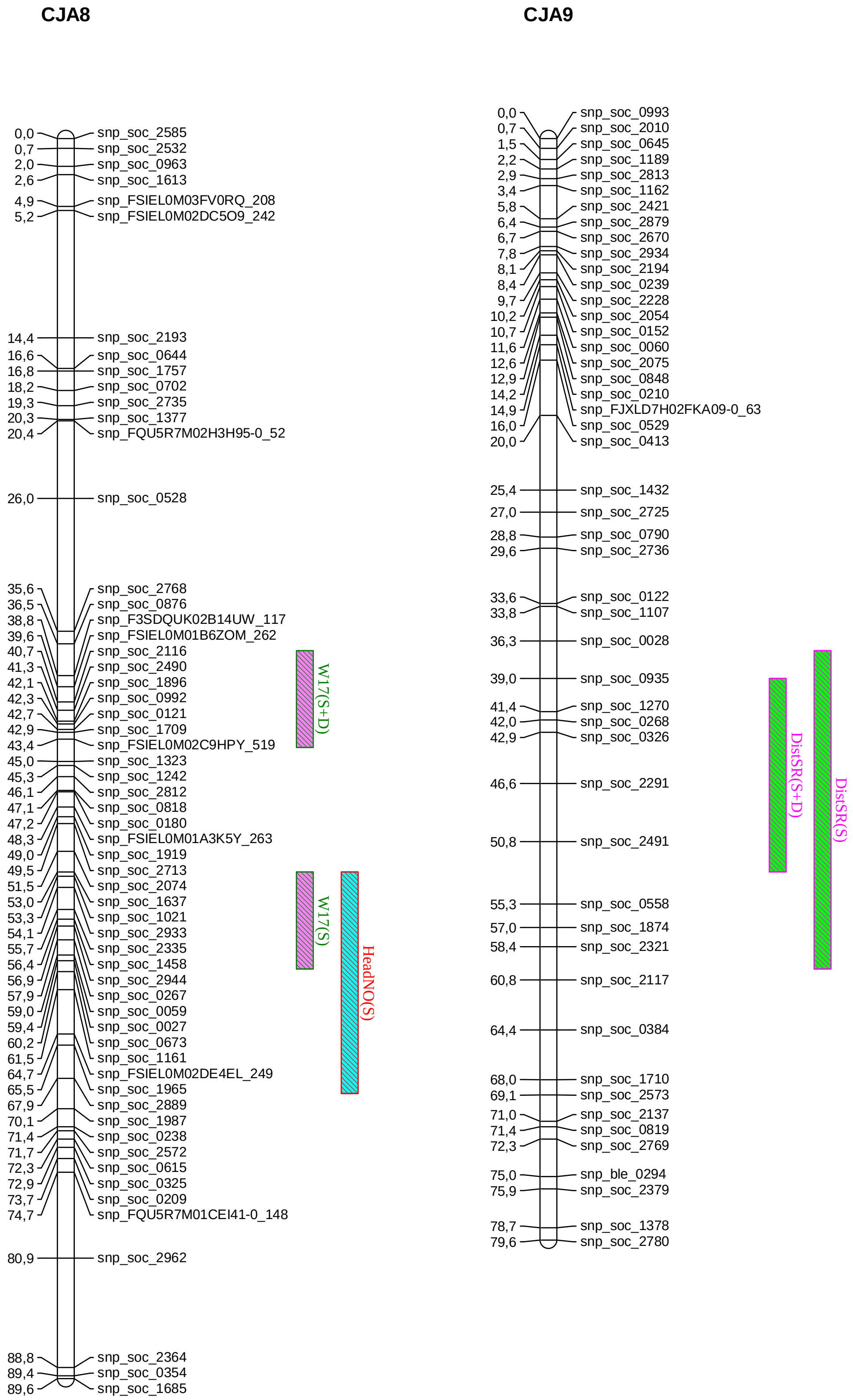
CJA8
0,0 snp_soc_2585
0,7 snp_soc_2532
2,0 snp_soc_0963
2,6 snp_soc_1613
4,9 snp_FSIEL0M03FV0RQ_208
5,2 snp_FSIEL0M02DC5O9_242
14,4 snp_soc_2193
16,6 snp_soc_0644
16,8 snp_soc_1757
18,2 snp_soc_0702
19,3 snp_soc_2735
20,3 snp_soc_1377
20,4 snp_FQU5R7M02H3H95-0_52
26,0 snp_soc_0528
35,6 snp_soc_2768
36,5 snp_soc_0876
38,8 snp_F3SDQUK02B14UW_117
39,6 snp_FSIEL0M01B6ZOM_262
40,7 snp_soc_2116
41,3 snp_soc_2490
42,1 snp_soc_1896
42,3 snp_soc_0992
42,7 snp_soc_0121
42,9 snp_soc_1709
43,4 snp_FSIEL0M02C9HPY_519
45,0 snp_soc_1323
45,3 snp_soc_1242
46,1 snp_soc_2812
47,1 snp_soc_0818
47,2 snp_soc_0180
48,3 snp_FSIEL0M01A3K5Y_263
49,0 snp_soc_1919
49,5 snp_soc_2713
51,5 snp_soc_2074
53,0 snp_soc_1637
53,3 snp_soc_1021
54,1 snp_soc_2933
55,7 snp_soc_2335
56,4 snp_soc_1458
56,9 snp_soc_2944
57,9 snp_soc_0267
59,0 snp_soc_0059
59,4 snp_soc_0027
60,2 snp_soc_0673
61,5 snp_soc_1161
64,7 snp_FSIEL0M02DE4EL_249
65,5 snp_soc_1965
67,9 snp_soc_2889
70,1 snp_soc_1987
71,4 snp_soc_0238
71,7 snp_soc_2572
72,3 snp_soc_0615
72,9 snp_soc_0325
73,7 snp_soc_0209
74,7 snp_FQU5R7M01CEI41-0_148
80,9 snp_soc_2962
88,8 snp_soc_2364
89,4 snp_soc_0354
89,6 snp_soc_1685
W17(S+D)
W17(S)
HeadNO(S)
CJA9
0,0 snp_soc_0993
0,7 snp_soc_2010
1,5 snp_soc_0645
2,2 snp_soc_1189
2,9 snp_soc_2813
3,4 snp_soc_1162
5,8 snp_soc_2421
6,4 snp_soc_2879
6,7 snp_soc_2670
7,8 snp_soc_2934
8,1 snp_soc_2194
8,4 snp_soc_0239
9,7 snp_soc_2228
10,2 snp_soc_2054
10,7 snp_soc_0152
11,6 snp_soc_0060
12,6 snp_soc_2075
12,9 snp_soc_0848
14,2 snp_soc_0210
14,9 snp_FJXLD7H02FKA09-0_63
16,0 snp_soc_0529
20,0 snp_soc_0413
25,4 snp_soc_1432
27,0 snp_soc_2725
28,8 snp_soc_0790
29,6 snp_soc_2736
33,6 snp_soc_0122
33,8 snp_soc_1107
36,3 snp_soc_0028
39,0 snp_soc_0935
41,4 snp_soc_1270
42,0 snp_soc_0268
42,9 snp_soc_0326
46,6 snp_soc_2291
50,8 snp_soc_2491
55,3 snp_soc_0558
57,0 snp_soc_1874
58,4 snp_soc_2321
60,8 snp_soc_2117
64,4 snp_soc_0384
68,0 snp_soc_1710
69,1 snp_soc_2573
71,0 snp_soc_2137
71,4 snp_soc_0819
72,3 snp_soc_2769
75,0 snp_ble_0294
75,9 snp_soc_2379
78,7 snp_soc_1378
79,6 snp_soc_2780
DistSR(S+D)
DistSR(S)

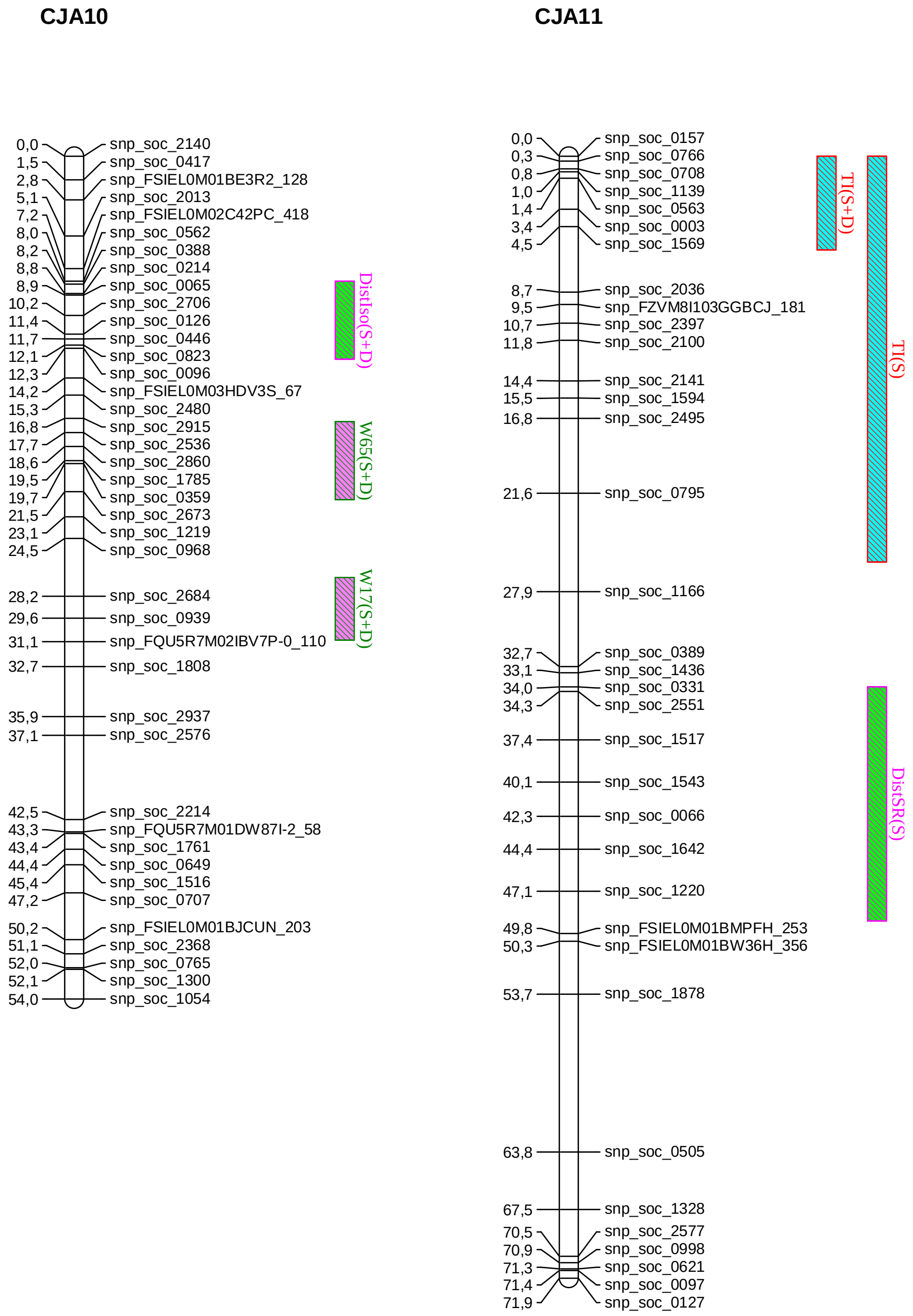

CJA10
0,0 snp_soc_2140
1,5 snp_soc_0417
2,8 snp_FSIEL0M01BE3R2_128
5,1 snp_soc_2013
7,2 snp_FSIEL0M02C42PC_418
8,0 snp_soc_0562
8,2 snp_soc_0388
8,8 snp_soc_0214
8,9 snp_soc_0065
10,2 snp_soc_2706
11,4 snp_soc_0126
11,7 snp_soc_0446
12,1 snp_soc_0823
12,3 snp_soc_0096
14,2 snp_FSIEL0M03HDV3S_67
15,3 snp_soc_2480
16,8 snp_soc_2915
17,7 snp_soc_2536
18,6 snp_soc_2860
19,5 snp_soc_1785
19,7 snp_soc_0359
21,5 snp_soc_2673
23,1 snp_soc_1219
24,5 snp_soc_0968
28,2 snp_soc_2684
29,6 snp_soc_0939
31,1 snp_FQU5R7M02IBV7P-0_110
32,7 snp_soc_1808
35,9 snp_soc_2937
37,1 snp_soc_2576
42,5 snp_soc_2214
43,3 snp_FQU5R7M01DW87I-2_58
43,4 snp_soc_1761
44,4 snp_soc_0649
45,4 snp_soc_1516
47,2 snp_soc_0707
50,2 snp_FSIEL0M01BJCUN_203
51,1 snp_soc_2368
52,0 snp_soc_0765
52,1 snp_soc_1300
54,0 snp_soc_1054
DistIso(S+D)
W65(S+D)
W17(S+D)
CJA11
0,0 snp_soc_0157
0,3 snp_soc_0766
0,8 snp_soc_0708
1,0 snp_soc_1139
1,4 snp_soc_0563
3,4 snp_soc_0003
4,5 snp_soc_1569
8,7 snp_soc_2036
9,5 snp_FZVM8I103GGBCJ_181
10,7 snp_soc_2397
11,8 snp_soc_2100
14,4 snp_soc_2141
15,5 snp_soc_1594
16,8 snp_soc_2495
21,6 snp_soc_0795
27,9 snp_soc_1166
32,7 snp_soc_0389
33,1 snp_soc_1436
34,0 snp_soc_0331
34,3 snp_soc_2551
37,4 snp_soc_1517
40,1 snp_soc_1543
42,3 snp_soc_0066
44,4 snp_soc_1642
47,1 snp_soc_1220
49,8 snp_FSIEL0M01BMPFH_253
50,3 snp_FSIEL0M01BW36H_356
53,7 snp_soc_1878
63,8 snp_soc_0505
67,5 snp_soc_1328
70,5 snp_soc_2577
70,9 snp_soc_0998
71,3 snp_soc_0621
71,4 snp_soc_0097
71,9 snp_soc_0127
TI(S+D)
TI(S)
DistSR(S)

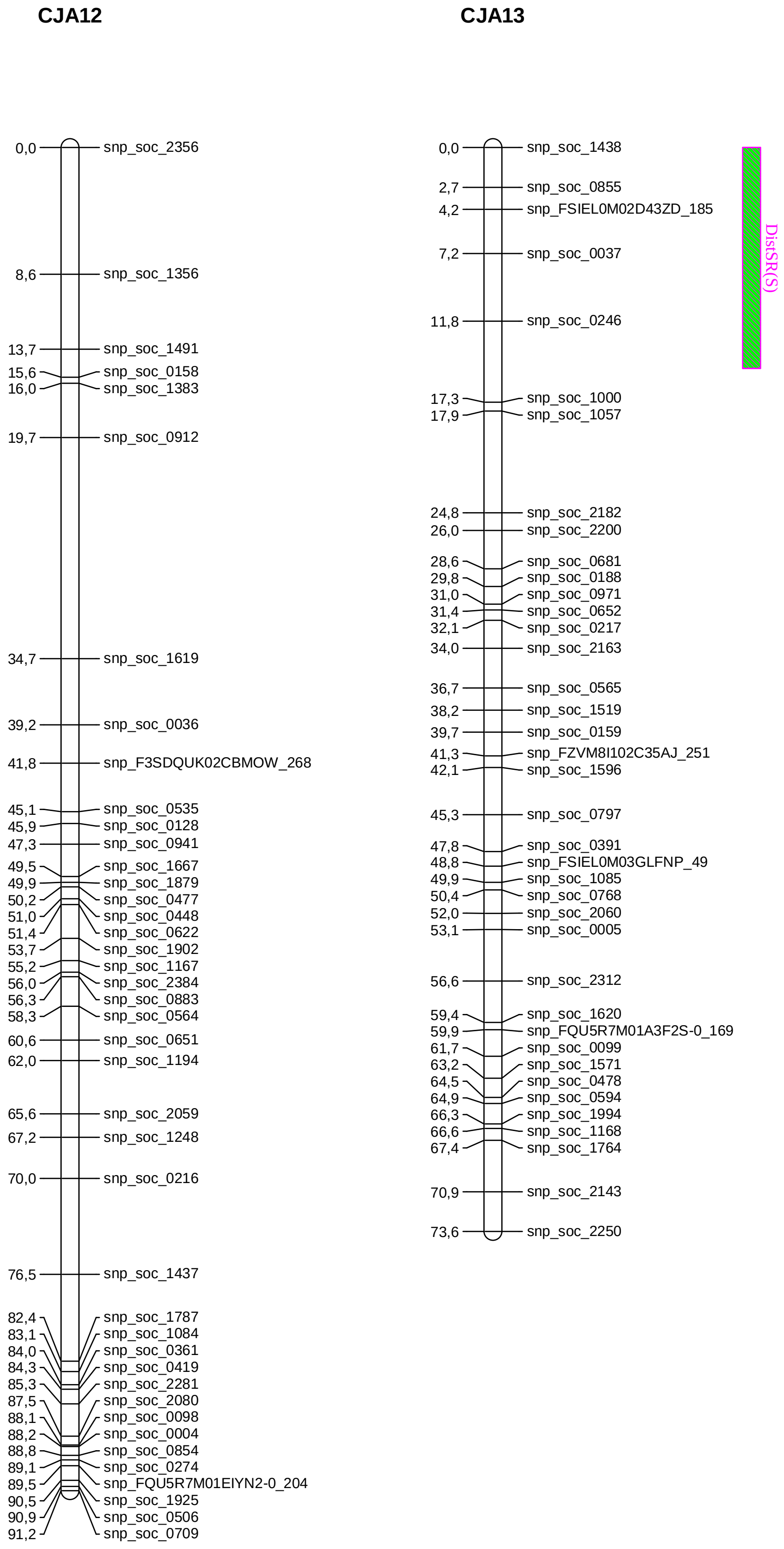
CJA12
0,0 snp_soc_2356
8,6 snp_soc_1356
13,7 snp_soc_1491
15,6 snp_soc_0158
16,0 snp_soc_1383
19,7 snp_soc_0912
34,7 snp_soc_1619
39,2 snp_soc_0036
41,8 snp_F3SDQUK02CBMOW_268
45,1 snp_soc_0535
45,9 snp_soc_0128
47,3 snp_soc_0941
49,5 snp_soc_1667
49,9 snp_soc_1879
50,2 snp_soc_0477
51,0 snp_soc_0448
51,4 snp_soc_0622
53,7 snp_soc_1902
55,2 snp_soc_1167
56,0 snp_soc_2384
56,3 snp_soc_0883
58,3 snp_soc_0564
60,6 snp_soc_0651
62,0 snp_soc_1194
65,6 snp_soc_2059
67,2 snp_soc_1248
70,0 snp_soc_0216
76,5 snp_soc_1437
82,4 snp_soc_1787
83,1 snp_soc_1084
84,0 snp_soc_0361
84,3 snp_soc_0419
85,3 snp_soc_2281
87,5 snp_soc_2080
88,1 snp_soc_0098
88,2 snp_soc_0004
88,8 snp_soc_0854
89,1 snp_soc_0274
89,5 snp_FQU5R7M01EIYN2-0_204
90,5 snp_soc_1925
90,9 snp_soc_0506
91,2 snp_soc_0709
CJA13
0,0 snp_soc_1438
2,7 snp_soc_0855
4,2 snp_FSIEL0M02D43ZD_185
7,2 snp_soc_0037
11,8 snp_soc_0246
17,3 snp_soc_1000
17,9 snp_soc_1057
24,8 snp_soc_2182
26,0 snp_soc_2200
28,6 snp_soc_0681
29,8 snp_soc_0188
31,0 snp_soc_0971
31,4 snp_soc_0652
32,1 snp_soc_0217
34,0 snp_soc_2163
36,7 snp_soc_0565
38,2 snp_soc_1519
39,7 snp_soc_0159
41,3 snp_FZVM8I102C35AJ_251
42,1 snp_soc_1596
45,3 snp_soc_0797
47,8 snp_soc_0391
48,8 snp_FSIEL0M03GLFNP_49
49,9 snp_soc_1085
50,4 snp_soc_0768
52,0 snp_soc_2060
53,1 snp_soc_0005
56,6 snp_soc_2312
59,4 snp_soc_1620
59,9 snp_FQU5R7M01A3F2S-0_169
61,7 snp_soc_0099
63,2 snp_soc_1571
64,5 snp_soc_0478
64,9 snp_soc_0594
66,3 snp_soc_1994
66,6 snp_soc_1168
67,4 snp_soc_1764
70,9 snp_soc_2143
73,6 snp_soc_2250
DistSR(S)

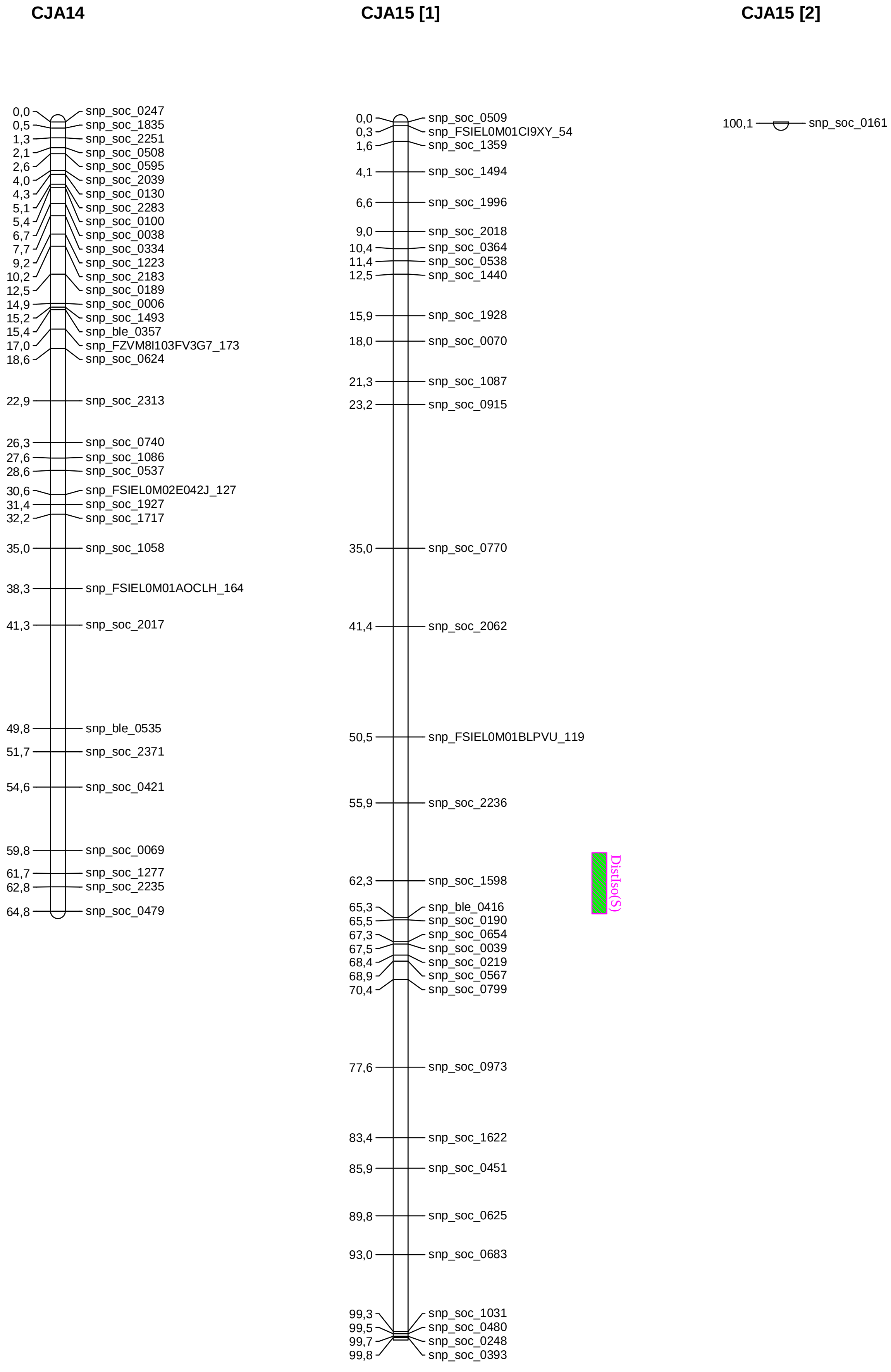

CJA14
0,0 snp_soc_0247
0,5 snp_soc_1835
1,3 snp_soc_2251
2,1 snp_soc_0508
2,6 snp_soc_0595
4,0 snp_soc_2039
4,3 snp_soc_0130
5,1 snp_soc_2283
5,4 snp_soc_0100
6,7 snp_soc_0038
7,7 snp_soc_0334
9,2 snp_soc_1223
10,2 snp_soc_2183
12,5 snp_soc_0189
14,9 snp_soc_0006
15,2 snp_soc_1493
15,4 snp_ble_0357
17,0 snp_FZVM8I103FV3G7_173
18,6 snp_soc_0624
22,9 snp_soc_2313
26,3 snp_soc_0740
27,6 snp_soc_1086
28,6 snp_soc_0537
30,6 snp_FSIEL0M02E042J_127
31,4 snp_soc_1927
32,2 snp_soc_1717
35,0 snp_soc_1058
38,3 snp_FSIEL0M01AOCLH_164
41,3 snp_soc_2017
49,8 snp_ble_0535
51,7 snp_soc_2371
54,6 snp_soc_0421
59,8 snp_soc_0069
61,7 snp_soc_1277
62,8 snp_soc_2235
64,8 snp_soc_0479
CJA15 [1]
0,0 snp_soc_0509
0,3 snp_FSIEL0M01CI9XY_54
1,6 snp_soc_1359
4,1 snp_soc_1494
6,6 snp_soc_1996
9,0 snp_soc_2018
10,4 snp_soc_0364
11,4 snp_soc_0538
12,5 snp_soc_1440
15,9 snp_soc_1928
18,0 snp_soc_0070
21,3 snp_soc_1087
23,2 snp_soc_0915
35,0 snp_soc_0770
41,4 snp_soc_2062
50,5 snp_FSIEL0M01BLPVU_119
55,9 snp_soc_2236
62,3 snp_soc_1598
65,3 snp_ble_0416
65,5 snp_soc_0190
67,3 snp_soc_0654
67,5 snp_soc_0039
68,4 snp_soc_0219
68,9 snp_soc_0567
70,4 snp_soc_0799
77,6 snp_soc_0973
83,4 snp_soc_1622
85,9 snp_soc_0451
89,8 snp_soc_0625
93,0 snp_soc_0683
99,3 snp_soc_1031
99,5 snp_soc_0480
99,7 snp_soc_0248
99,8 snp_soc_0393
DistIso(S)
CJA15 [2]
100,1 snp_soc_0161

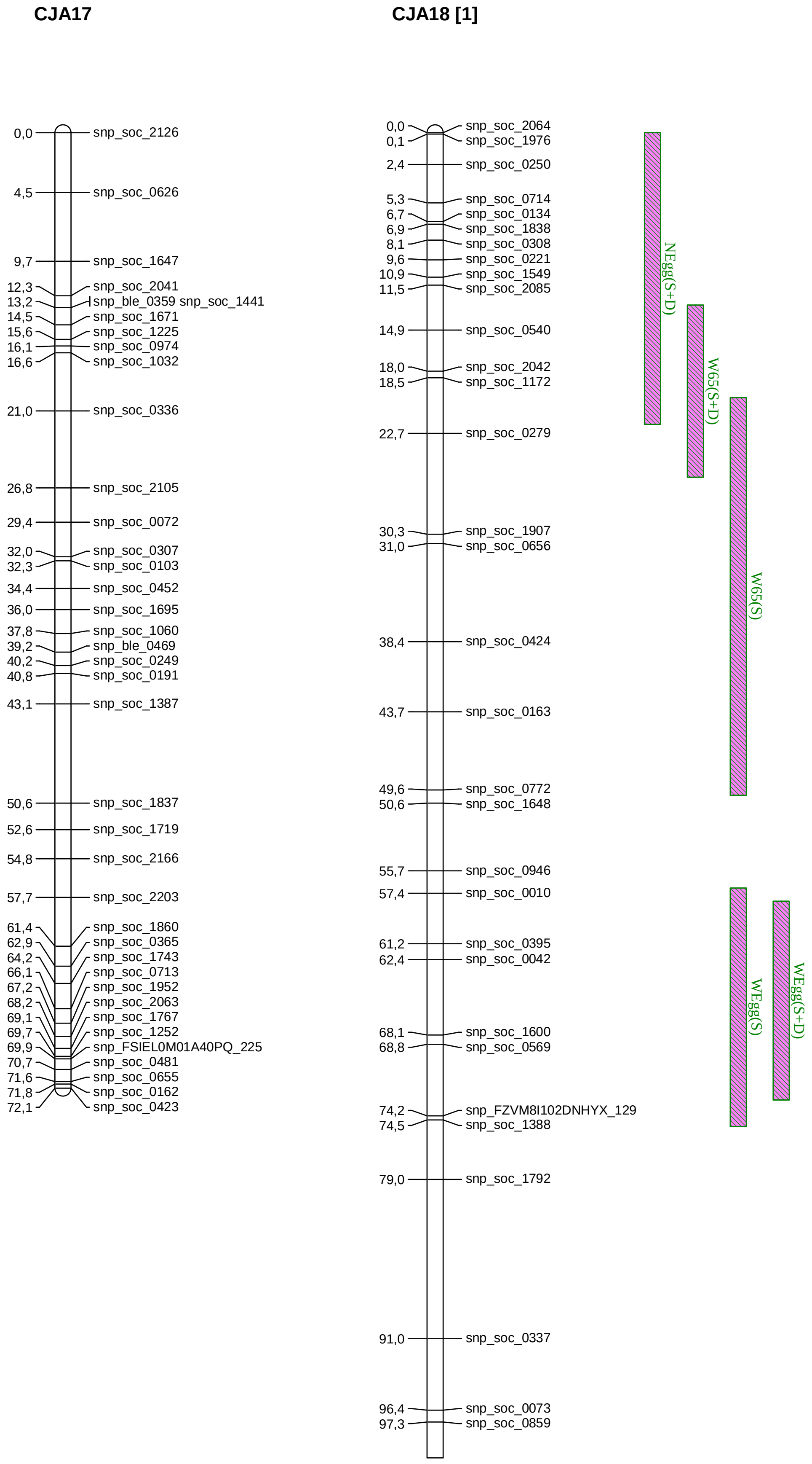

CJA17
CJA18 [1]
0,0 snp_soc_2126
4,5 snp_soc_0626
9,7 snp_soc_1647
12,3 snp_soc_2041
13,2 snp_ble_0359 snp_soc_1441
14,5 snp_soc_1671
15,6 snp_soc_1225
16,1 snp_soc_0974
16,6 snp_soc_1032
21,0 snp_soc_0336
26,8 snp_soc_2105
29,4 snp_soc_0072
32,0 snp_soc_0307
32,3 snp_soc_0103
34,4 snp_soc_0452
36,0 snp_soc_1695
37,8 snp_soc_1060
39,2 snp_ble_0469
40,2 snp_soc_0249
40,8 snp_soc_0191
43,1 snp_soc_1387
50,6 snp_soc_1837
52,6 snp_soc_1719
54,8 snp_soc_2166
57,7 snp_soc_2203
61,4 snp_soc_1860
62,9 snp_soc_0365
64,2 snp_soc_1743
66,1 snp_soc_0713
67,2 snp_soc_1952
68,2 snp_soc_2063
69,1 snp_soc_1767
69,7 snp_soc_1252
69,9 snp_FSIEL0M01A40PQ_225
70,7 snp_soc_0481
71,6 snp_soc_0655
71,8 snp_soc_0162
72,1 snp_soc_0423
0,0 snp_soc_2064
0,1 snp_soc_1976
2,4 snp_soc_0250
5,3 snp_soc_0714
6,7 snp_soc_0134
6,9 snp_soc_1838
8,1 snp_soc_0308
9,6 snp_soc_0221
10,9 snp_soc_1549
11,5 snp_soc_2085
14,9 snp_soc_0540
18,0 snp_soc_2042
18,5 snp_soc_1172
22,7 snp_soc_0279
30,3 snp_soc_1907
31,0 snp_soc_0656
38,4 snp_soc_0424
43,7 snp_soc_0163
49,6 snp_soc_0772
50,6 snp_soc_1648
55,7 snp_soc_0946
57,4 snp_soc_0010
61,2 snp_soc_0395
62,4 snp_soc_0042
68,1 snp_soc_1600
68,8 snp_soc_0569
74,2 snp_FZVM8I102DNHYX_129
74,5 snp_soc_1388
79,0 snp_soc_1792
91,0 snp_soc_0337
96,4 snp_soc_0073
97,3 snp_soc_0859
NEgg(S+D)
W65(S+D)
W65(S)
WEgg(S)
WEgg(S+D)

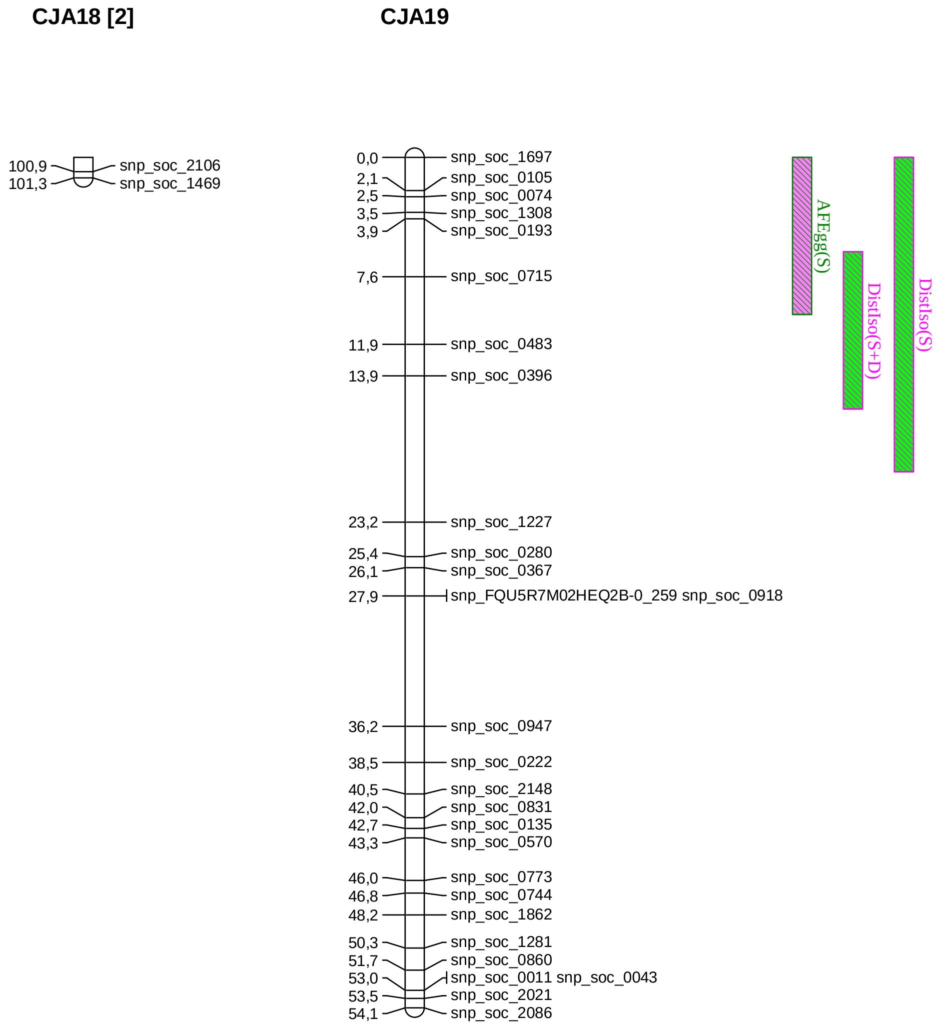

CJA18 [2]
CJA19
100,9
snp_soc_2106
101,3
snp_soc_1469
0,0
snp_soc_1697
2,1
snp_soc_0105
2,5
snp_soc_0074
3,5
snp_soc_1308
3,9
snp_soc_0193
7,6
snp_soc_0715
11,9
snp_soc_0483
13,9
snp_soc_0396
23,2
snp_soc_1227
25,4
snp_soc_0280
26,1
snp_soc_0367
27,9
snp_FQU5R7M02HEQ2B-0_259 snp_soc_0918
36,2
snp_soc_0947
38,5
snp_soc_0222
40,5
snp_soc_2148
42,0
snp_soc_0831
42,7
snp_soc_0135
43,3
snp_soc_0570
46,0
snp_soc_0773
46,8
snp_soc_0744
48,2
snp_soc_1862
50,3
snp_soc_1281
51,7
snp_soc_0860
53,0
snp_soc_0011 snp_soc_0043
53,5
snp_soc_2021
54,1
snp_soc_2086
AFEgg(S)
DistIso(S+D)
DistIso(S)

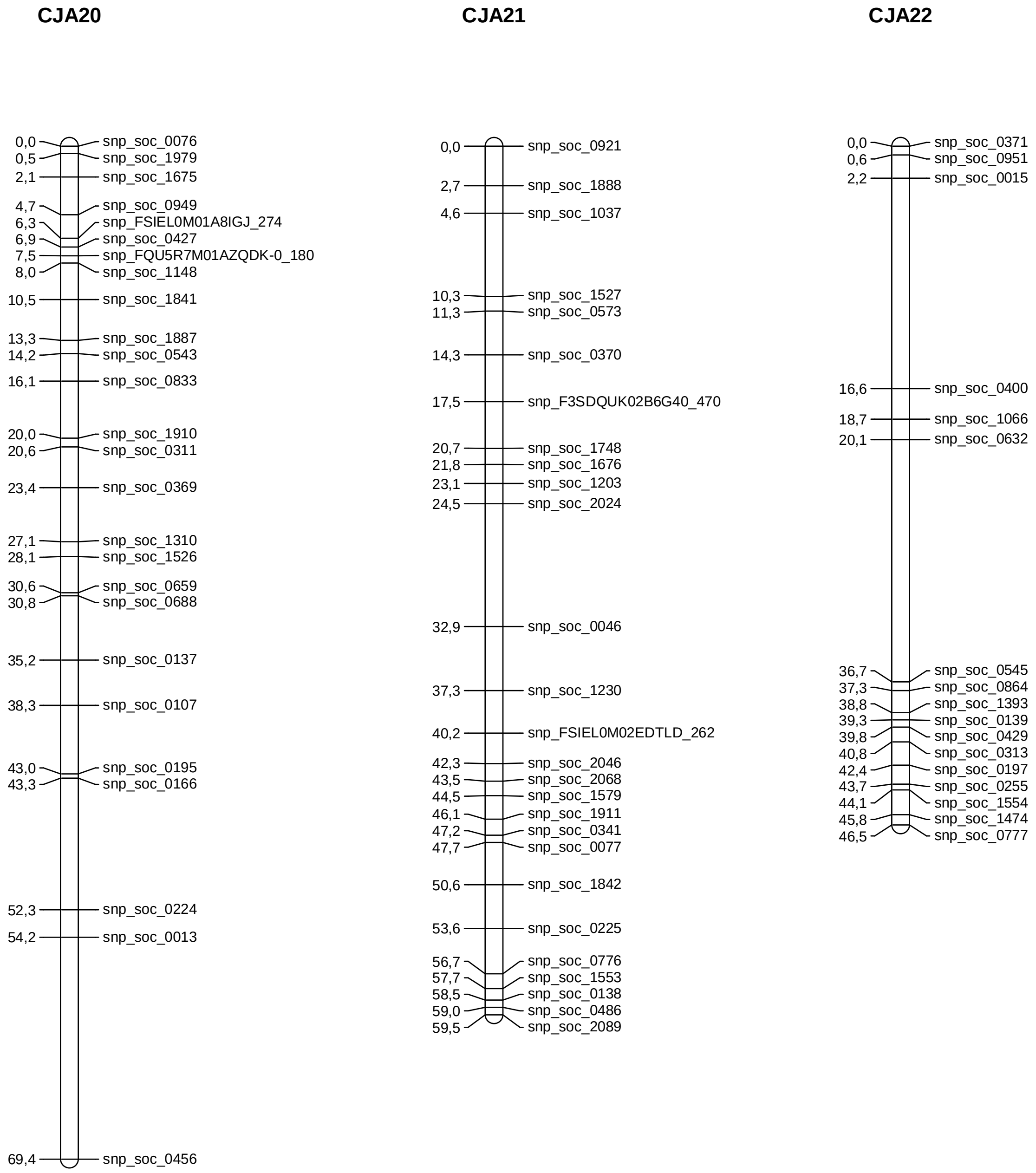
CJA20
0,0 snp_soc_0076
0,5 snp_soc_1979
2,1 snp_soc_1675
4,7 snp_soc_0949
6,3 snp_FSIEL0M01A8IGJ_274
6,9 snp_soc_0427
7,5 snp_FQU5R7M01AZQDK-0_180
8,0 snp_soc_1148
10,5 snp_soc_1841
13,3 snp_soc_1887
14,2 snp_soc_0543
16,1 snp_soc_0833
20,0 snp_soc_1910
20,6 snp_soc_0311
23,4 snp_soc_0369
27,1 snp_soc_1310
28,1 snp_soc_1526
30,6 snp_soc_0659
30,8 snp_soc_0688
35,2 snp_soc_0137
38,3 snp_soc_0107
43,0 snp_soc_0195
43,3 snp_soc_0166
52,3 snp_soc_0224
54,2 snp_soc_0013
69,4 snp_soc_0456
CJA21
0,0 snp_soc_0921
2,7 snp_soc_1888
4,6 snp_soc_1037
10,3 snp_soc_1527
11,3 snp_soc_0573
14,3 snp_soc_0370
17,5 snp_F3SDQUK02B6G40_470
20,7 snp_soc_1748
21,8 snp_soc_1676
23,1 snp_soc_1203
24,5 snp_soc_2024
32,9 snp_soc_0046
37,3 snp_soc_1230
40,2 snp_FSIEL0M02EDTLD_262
42,3 snp_soc_2046
43,5 snp_soc_2068
44,5 snp_soc_1579
46,1 snp_soc_1911
47,2 snp_soc_0341
47,7 snp_soc_0077
50,6 snp_soc_1842
53,6 snp_soc_0225
56,7 snp_soc_0776
57,7 snp_soc_1553
58,5 snp_soc_0138
59,0 snp_soc_0486
59,5 snp_soc_2089
CJA22
0,0 snp_soc_0371
0,6 snp_soc_0951
2,2 snp_soc_0015
16,6 snp_soc_0400
18,7 snp_soc_1066
20,1 snp_soc_0632
36,7 snp_soc_0545
37,3 snp_soc_0864
38,8 snp_soc_1393
39,3 snp_soc_0139
39,8 snp_soc_0429
40,8 snp_soc_0313
42,4 snp_soc_0197
43,7 snp_soc_0255
44,1 snp_soc_1554
45,8 snp_soc_1474
46,5 snp_soc_0777

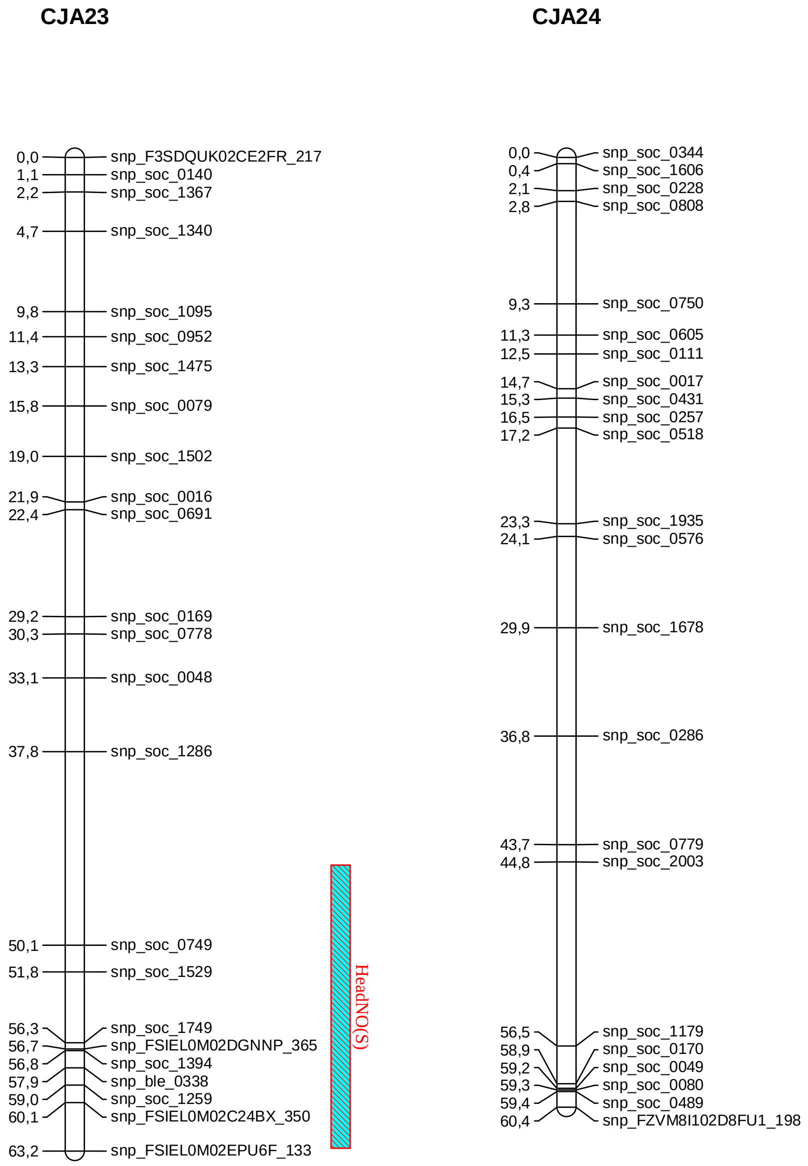
CJA23
0,0 snp_F3SDQUK02CE2FR_217
1,1 snp_soc_0140
2,2 snp_soc_1367
4,7 snp_soc_1340
9,8 snp_soc_1095
11,4 snp_soc_0952
13,3 snp_soc_1475
15,8 snp_soc_0079
19,0 snp_soc_1502
21,9 snp_soc_0016
22,4 snp_soc_0691
29,2 snp_soc_0169
30,3 snp_soc_0778
33,1 snp_soc_0048
37,8 snp_soc_1286
50,1 snp_soc_0749
51,8 snp_soc_1529
56,3 snp_soc_1749
56,7 snp_FSIEL0M02DGNNP_365
56,8 snp_soc_1394
57,9 snp_ble_0338
59,0 snp_soc_1259
60,1 snp_FSIEL0M02C24BX_350
63,2 snp_FSIEL0M02EPU6F_133
HeadNO(S)
CJA24
0,0 snp_soc_0344
0,4 snp_soc_1606
2,1 snp_soc_0228
2,8 snp_soc_0808
9,3 snp_soc_0750
11,3 snp_soc_0605
12,5 snp_soc_0111
14,7 snp_soc_0017
15,3 snp_soc_0431
16,5 snp_soc_0257
17,2 snp_soc_0518
23,3 snp_soc_1935
24,1 snp_soc_0576
29,9 snp_soc_1678
36,8 snp_soc_0286
43,7 snp_soc_0779
44,8 snp_soc_2003
56,5 snp_soc_1179
58,9 snp_soc_0170
59,2 snp_soc_0049
59,3 snp_soc_0080
59,4 snp_soc_0489
60,4 snp_FZVM8I102D8FU1_198

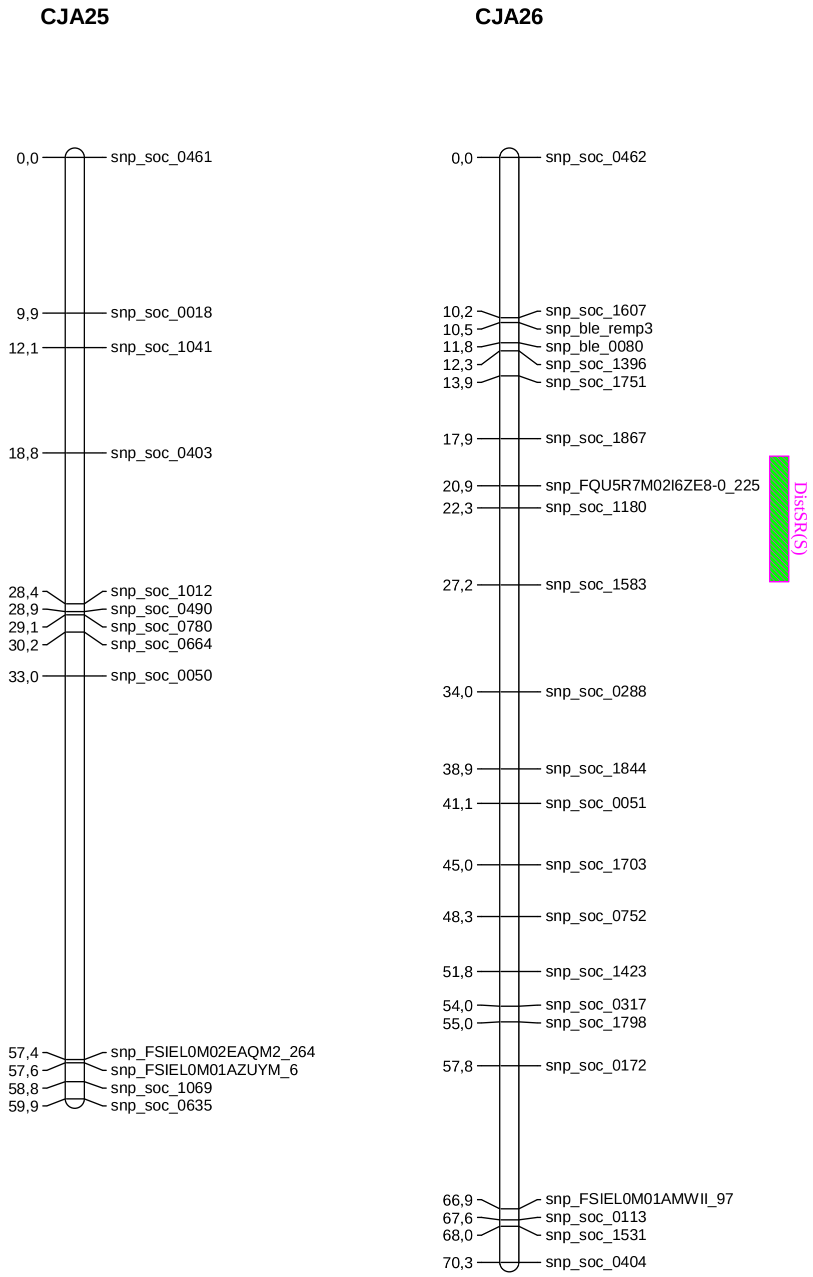
CJA25
CJA26
0,0 snp_soc_0461
9,9 snp_soc_0018
12,1 snp_soc_1041
18,8 snp_soc_0403
28,4 snp_soc_1012
28,9 snp_soc_0490
29,1 snp_soc_0780
30,2 snp_soc_0664
33,0 snp_soc_0050
57,4 snp_FSIEL0M02EAQM2_264
57,6 snp_FSIEL0M01AZUYM_6
58,8 snp_soc_1069
59,9 snp_soc_0635
0,0 snp_soc_0462
10,2 snp_soc_1607
10,5 snp_ble_remp3
11,8 snp_ble_0080
12,3 snp_soc_1396
13,9 snp_soc_1751
17,9 snp_soc_1867
20,9 snp_FQU5R7M02I6ZE8-0_225
22,3 snp_soc_1180
27,2 snp_soc_1583
34,0 snp_soc_0288
38,9 snp_soc_1844
41,1 snp_soc_0051
45,0 snp_soc_1703
48,3 snp_soc_0752
51,8 snp_soc_1423
54,0 snp_soc_0317
55,0 snp_soc_1798
57,8 snp_soc_0172
66,9 snp_FSIEL0M01AMWII_97
67,6 snp_soc_0113
68,0 snp_soc_1531
70,3 snp_soc_0404
DistSR(S)

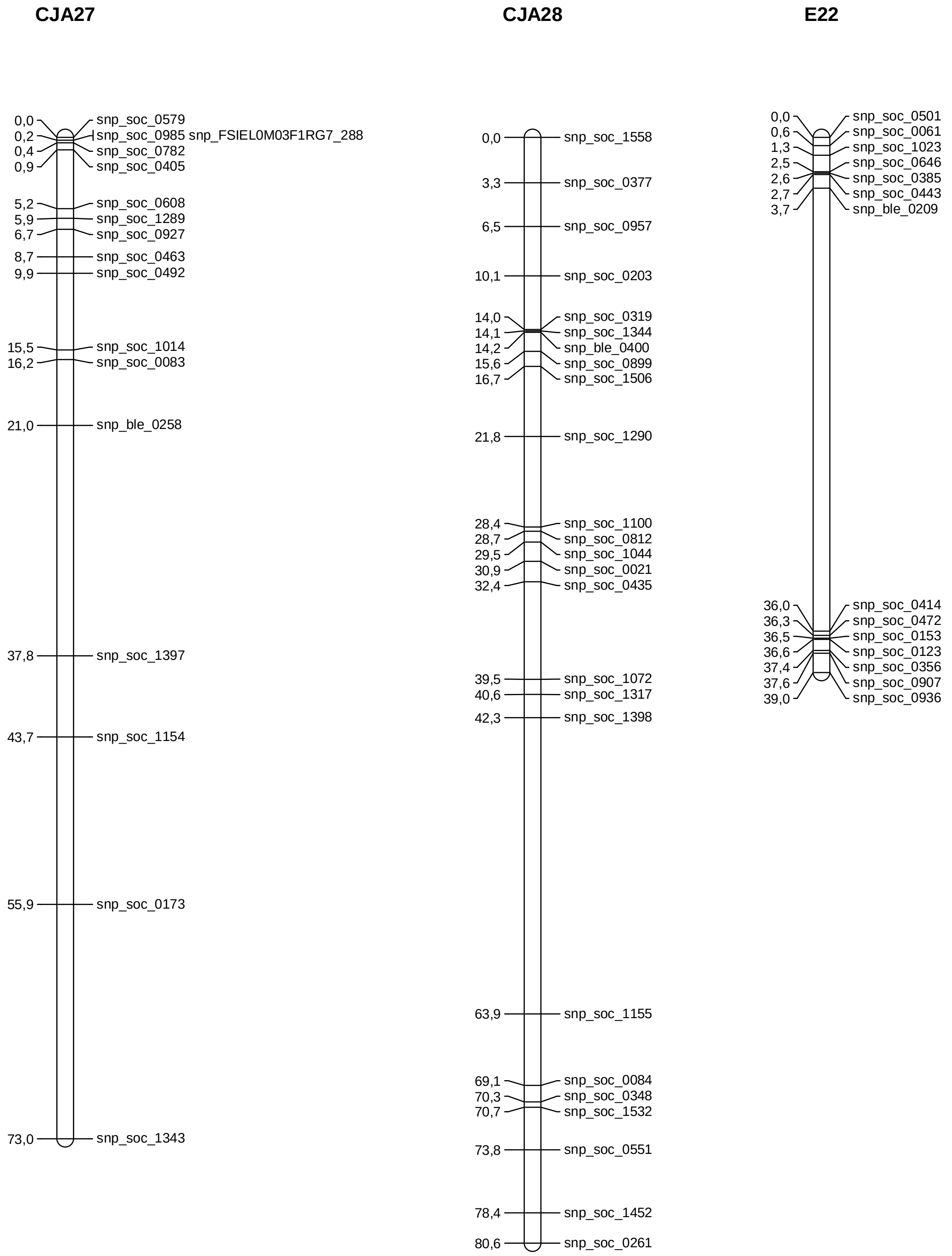
CJA27
0,0 snp_soc_0579
0,2 snp_soc_0985 snp_FSIEL0M03F1RG7_288
0,4 snp_soc_0782
0,9 snp_soc_0405
5,2 snp_soc_0608
5,9 snp_soc_1289
6,7 snp_soc_0927
8,7 snp_soc_0463
9,9 snp_soc_0492
15,5 snp_soc_1014
16,2 snp_soc_0083
21,0 snp_ble_0258
37,8 snp_soc_1397
43,7 snp_soc_1154
55,9 snp_soc_0173
73,0 snp_soc_1343
CJA28
0,0 snp_soc_1558
3,3 snp_soc_0377
6,5 snp_soc_0957
10,1 snp_soc_0203
14,0 snp_soc_0319
14,1 snp_soc_1344
14,2 snp_ble_0400
15,6 snp_soc_0899
16,7 snp_soc_1506
21,8 snp_soc_1290
28,4 snp_soc_1100
28,7 snp_soc_0812
29,5 snp_soc_1044
30,9 snp_soc_0021
32,4 snp_soc_0435
39,5 snp_soc_1072
40,6 snp_soc_1317
42,3 snp_soc_1398
63,9 snp_soc_1155
69,1 snp_soc_0084
70,3 snp_soc_0348
70,7 snp_soc_1532
73,8 snp_soc_0551
78,4 snp_soc_1452
80,6 snp_soc_0261
E22
0,0 snp_soc_0501
0,6 snp_soc_0061
1,3 snp_soc_1023
2,5 snp_soc_0646
2,6 snp_soc_0385
2,7 snp_soc_0443
3,7 snp_ble_0209
36,0 snp_soc_0414
36,3 snp_soc_0472
36,5 snp_soc_0153
36,6 snp_soc_0123
37,4 snp_soc_0356
37,6 snp_soc_0907
39,0 snp_soc_0936

Additional Figure 4 ï Quail genetic map with positioned QTL.

Graphic representation of the genetic map of the Quail with distance expressed in centiMorgan (cM). QTL are positioned using the coordinates of their confidence interval and illustrated by colored boxes with with pink for social motivation related QTL, red for emotional reactivity QTL, blue for aggressiveness QTL, and in green for production QTL. Genome-wide significant QTL are represented in filled boxes and chromosome-wide significant QTL in shaded boxes.

DistSR: Distance travelled on the treadmill in the social reinstatement behavior test; **DistIso**: Distance travelled in periphery in the social isolation test; **TI**: Time spent immobile in the tonic immobility test; **HeadNO**: Number of scans when the Quail passed its head through the wire of the front of the cage in the novel object test; **AgrP**: Number of aggressive pecks in the aggressive behavior test; **W17**: weight at 17 days; **W65**: weight at 65 days; **AFEgg**: Age at first egg; **NEgg**: Number of eggs laid; **WEgg**: Mean egg weight.
